# Role of telomere dysfunction and immune infiltration in idiopathic pulmonary fibrosis: new insights from bioinformatics analysis

**DOI:** 10.3389/fgene.2024.1447296

**Published:** 2024-09-13

**Authors:** Chenkun Fu, Xin Tian, Shuang Wu, Xiaojuan Chu, Yiju Cheng, Xiao Wu, Wengting Yang

**Affiliations:** ^1^ Department of Respiratory and Critical Care Medicine, The Affiliated Hospital of Guizhou Medical University, Guiyang, China; ^2^ Department of Respiratory and Critical Care Medicine, The Fourth People’s Hospital of Guiyang, Guiyang, China; ^3^ Department of Critical Care Medicine, The Second People’s Hospital of Guiyang, Guiyang, China

**Keywords:** idiopathic pulmonary fibrosis, telomere, immune infiltration, WGCNA, GEO, summary data-based Mendelian randomization analysis, GPA33

## Abstract

**Background:**

Idiopathic pulmonary fibrosis (IPF) is a chronic progressive interstitial lung disease characterized by unexplained irreversible pulmonary fibrosis. Although the etiology of IPF is unclear, studies have shown that it is related to telomere length shortening. However, the prognostic value of telomere-related genes in IPF has not been investigated.

**Methods:**

We utilized the GSE10667 and GSE110147 datasets as the training set, employing differential expression analysis and weighted gene co-expression network analysis (WGCNA) to screen for disease candidate genes. Then, we used consensus clustering analysis to identify different telomere patterns. Next, we used summary data-based mendelian randomization (SMR) analysis to screen core genes. We further evaluated the relationship between core genes and overall survival and lung function in IPF patients. Finally, we performed immune infiltration analysis to reveal the changes in the immune microenvironment of IPF.

**Results:**

Through differential expression analysis and WGCNA, we identified 35 significant telomere regulatory factors. Consensus clustering analysis revealed two distinct telomere patterns, consisting of cluster A (n = 26) and cluster B (n = 19). Immune infiltration analysis revealed that cluster B had a more active immune microenvironment, suggesting its potential association with IPF. Using GTEx eQTL data, our SMR analysis identified two genes with potential causal associations with IPF, including GPA33 (P_SMR_ = 0.0013; P_HEIDI_ = 0.0741) and MICA (P_SMR_ = 0.0112; P_HEIDI_ = 0.9712). We further revealed that the expression of core genes is associated with survival time and lung function in IPF patients. Finally, immune infiltration analysis revealed that NK cells were downregulated and plasma cells and memory B cells were upregulated in IPF. Further correlation analysis showed that GPA33 expression was positively correlated with NK cells and negatively correlated with plasma cells and memory B cells.

**Conclusion:**

Our study provides a new perspective for the role of telomere dysfunction and immune infiltration in IPF and identifies potential therapeutic targets. Further research may reveal how core genes affect cell function and disease progression, providing new insights into the complex mechanisms of IPF.

## 1 Introduction

Idiopathic pulmonary fibrosis (IPF) is a chronic progressive interstitial lung disease characterized by unexplained irreversible pulmonary fibrosis. Its pathological feature is abnormal deposition of extracellular matrix, leading to the destruction of normal lung structure and progressive decline of lung function (Glass et al., 2020). Currently, alveolar epithelial damage and aberrant wound repair are increasingly recognized as important contributors to its pathogenesis (Moss et al., 2022). Studies have shown that persistent microdamage of senescent alveolar epithelial cells leads to abnormal signals between epithelial cells and fibroblasts, which eventually leads to fibroblast activation and myofibroblast differentiation to create extracellular matrix rich in collagen (Mei et al., 2021; Yao et al., 2021; Heukels et al., 2019). The clinical manifestations of IPF are progressive dyspnea and significantly reduced lung compliance (Schäfer et al., 2020). The diagnosis of IPF is mainly based on the clinical characteristics, imaging features and lung biopsy (Lynch et al., 2018). However, early diagnosis of IPF is difficult because the initial symptoms are atypical and overlap with many other common symptoms. Due to the lack of early accurate diagnostic markers and effective treatment measures, most IPF patients have a progressive deterioration and poor prognosis. The average life expectancy of untreated patients after diagnosis is 2–3 years (Barratt et al., 2018). At present, there is no cure for IPF, and the treatment is still mainly to slow down the progression of fibrosis. Two anti-fibrotic drugs (including nintedanib and pirfenidone) have been used in the treatment of IPF patients, but these two drugs have limited efficacy in preventing disease progression and improving quality of life, and there are tolerance-related problems (Fournier et al., 2022; Spagnolo et al., 2021). So far, lung transplantation is still the only effective treatment for IPF patients, but due to various factors, it is only a feasible choice for a few of them (Glass et al., 2022; George et al., 2019). IPF patients eventually die of respiratory failure, usually during acute episodes or due to other complications, such as lung cancer or thromboembolism (Caminati et al., 2019; King and Nathan, 2017). Therefore, exploring new biomarkers for early diagnosis of IPF patients is crucial for improving the survival time of IPF patients.

Although the etiology of the disease is not clear, it has been found to be closely related to shorter telomere length (Duckworth et al., 2021). It is estimated that about 8%–15% of familial IPF patients have mutations in telomerase or telomere protection proteins (Petrovski et al., 2017). Telomeres are protective structures consisting of DNA repeats (TTAGGG) at the end of chromosomes in eukaryotes, which are crucial for preserving the integrity and stability of chromosome structure and function (Smith et al., 2020). When telomere shortening is below the critical threshold, chromosomal instability and DNA damage response (DDR) pathways are activated, leading to cell senescence and apoptosis (d'Adda di Fagagna, 2008). The protective structure of telomeres (Shelterin and telomerase) protects telomeres by inhibiting telomere DDR and entering senescence (Xie et al., 2015; Rivera et al., 2017). Shelterin is a multiprotein complex composed of six telomere proteins (TRF1, TRF2, RAP1, TIN2, TPP1 and POT1) that bind to telomere DNA sequences and are essential for maintaining telomere length and integrity (de Lange, 2018). In animal models, the loss of TRF1 in alveolar monolayer squamous epithelium type 2 (AEC2) cells leads to AEC2 senescence and increased TGF-β1 levels in the lungs (Naikawadi et al., 2016). Similarly, degradation of TPP1 can aggravate stress-induced cell senescence and pulmonary fibrosis by promoting AEC2 telomere uncapping (Wang et al., 2020). A study on IPF with or without TERT mutation shows that AEC2 has significantly shorter telomeres, while other cells around it do not (Snetselaar et al., 2017). These studies suggest that telomere dysfunction may accelerate the occurrence of pulmonary fibrosis by affecting AEC2. Interestingly, Le Saux et al. proved that activation of telomerase with small molecules telomerase activator GRN510 can reduce bleomycin-induced pulmonary fibrosis in mice (Le Saux et al., 2013). Overall, these studies have determined that telomere dysfunction is the main driver of IPF.

**FIGURE 1:**
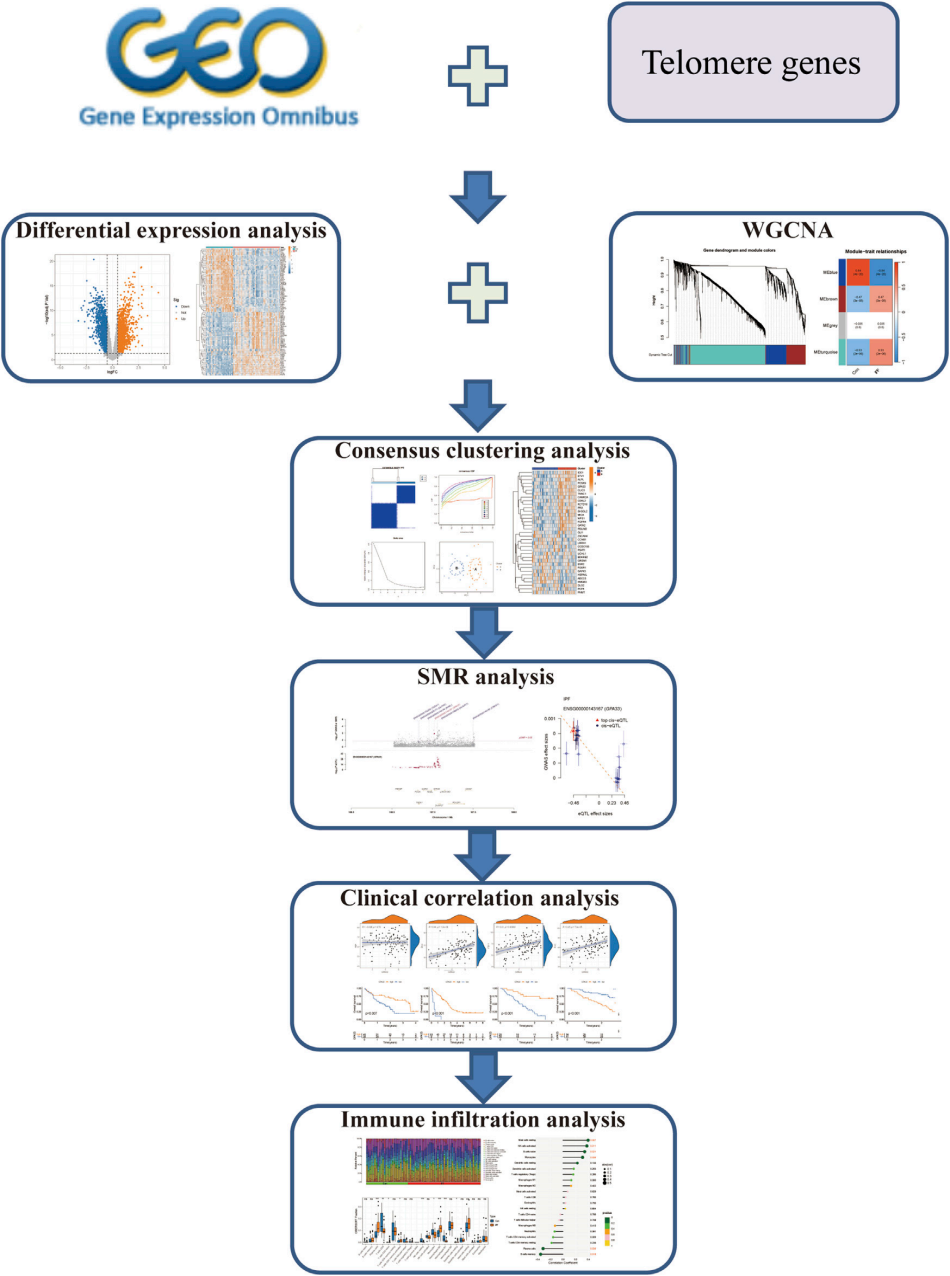
The work flow chart of our research.

Previous investigations have mainly concentrated on the length of telomere in IPF and its role in the prognosis of IPF. At present, there is no research to explore the role of telomere-related genes in IPF. This study comprehensively evaluated the role of telomere-related genes in the subtype classification and prognosis of IPF based on the GEO database. The abnormal regulation of telomere-related genes may become a potential therapeutic target for IPF. Our study provides a theoretical basis for the development of new therapeutic strategies and interventions. The work flow chart is shown in Figure 1.

## 2 Article types

Original Research Article.

## 3 Materials and methods

### 3.1 Data download and preprocessing

We retrieved eight IPF sample datasets from the GEO database (https://www.ncbi.nlm.nih.gov/geo/), including GSE10667 (Konishi et al., 2009), GSE110147 (Cecchini et al., 2018), GSE47460 (Tan et al., 2016), GSE32537 (Yang et al., 2013), GSE38958 (Huang et al., 2014), GSE28042 (Herazo-Maya et al., 2013), GSE93606 (Molyneaux et al., 2017), and GSE70866 (Prasse et al., 2019) (Table 1). We used the GSE10667 and GSE110147 datasets as the training set to screen for core genes. For survival analysis, we utilized the GSE28042, GSE93606, and GSE70866 datasets, which contain survival data of IPF patients. The GSE47460 dataset, which includes age and lung function data, was employed for clinical correlation analysis. After transforming the dataset’s ID, the batch effect between the two merged datasets was eliminated using the “sva” package. Finally, a total of 2093 telomere genes were retrieved from the TelNet database (http://www.cancertelsys.org/telnet/) (Supplementary Table S1).

**TABLE 1:** | Details of the data set used in this study.

Dataset	Plateform	Control	Case	Source	Publication years	Used for
GSE10667	GPL4133	15	23	lung tissue	2009	Training set
GSE110147	GPL6244	11	22	lung tissue	2018	Training set
GSE47460	GPL6480	17	38	lung tissue	2013	Validation set
GSE47460	GPL14550	91	122	lung tissue	2013	Validation set
GSE32537	GPL6244	50	119	lung tissue	2013	Validation set
GSE38958	GPL5175	45	70	PBMC	2014	Validation set
GSE28042	GPL6480	19	75	PBMC	2013	Survival analysis
GSE93606	GPL11532	20	154	PBMC	2017	Survival analysis
GSE70866	GPL14550	20	112	BALF	2018	Survival analysis

### 3.2 Differential expression and enrichment analysis

Using the “limma” package of R software, differentially expressed genes (DEGs) were screened with |log_2_FC| > 0.5 and adjusted *p*-value <0.05 as filtering conditions. The R software’s “pheatmap” and “ggplot2” packages were used to create the heatmap and volcanic map. The telomere-related DEGs were obtained by intersecting the DEGs with the telomere genes, and enrichment analysis was performed using the “clusterProfiler” package of R software.

### 3.3 Weighted gene co-expression network analysis (WGCNA)

To ensure the accuracy of the results, we utilized the “WGCNA” package of R software to filter out the co-expression modules and chose the top 25% of the genes with the highest variance for further study. After clustering the samples, the sample clustering tree diagram was obtained, and the soft threshold correlation scatter plot of the scale independence and mean connectivity was drawn. The soft threshold was then used to construct the weighted adjacency matrix, and it was subsequently transformed into a topological overlap matrix (TOM). When the minimal number of genes was 100, we utilized the TOM dissimilarity degree (1-TOM) of the hierarchical clustering tree approach to get the module. Then we obtained a heatmap of the module genes, with each module randomly assigned a color. Lastly, we determined the values for module membership and gene significance, and we created a scatter plot for each module using these data.

### 3.4 Consensus clustering analysis

Based on the selected 35 disease candidate genes, 45 IPF samples were divided into different clusters by consensus clustering analysis using the “k-means” algorithm in the “ConsensusClusterPlus” package of R software. In order to thoroughly assess the ideal number of clusters, we created the consensus matrix, cumulative distribution function(CDF) graph and delta area plot with the maximum number of clusters k = 9 as the limit. The “pheatmap” and “ggplot2” packages were used to generate heatmap and PCA maps to describe the expression of different clusters and determine the fitness of the clusters.

### 3.5 Immune infiltration analysis

Single sample gene set enrichment analysis (ssGSEA) is a variant of the GSEA method, which is used to determine the enrichment fraction of each sample and gene set pair. We used the “gsva” function in the “GSVA” package for ssGSEA, and set the parameters method = “ssgsea” and kcdf = “Gaussian”. Then we compared the immune cell abundance between different clusters in the IPF group, and the results were displayed using heatmaps and boxplots. We further used the CIBERSORT algorithm to estimate the relative abundance of immune cell types in the sample. The “CIBERSORT” function was used for analysis, with 1,000 permutations and quantile normalization enabled. Then we screened the results and only those with *P* values less than 0.05 were included. The box plot was drawn using the “ggpubr” package to show the immune infiltration of different samples. In order to further evaluate the relationship between core genes and immune cells, we used the “cor.test” function for spearman correlation analysis.

### 3.6 Summary data-based Mendelian randomization analysis

To explore the causal relationship between telomere candidate genes and IPF, we conducted a summary data-based Mendelian randomization (SMR) analysis. This method integrates summary data from genome-wide association studies (GWAS) and expression quantitative trait loci (eQTL) studies to determine the pleiotropic effects of genetic variants on the expression of telomere candidate genes and IPF. We obtained GWAS summary statistics for IPF from the GWAS Catalog database (https://www.ebi.ac.uk/gwas/home). Additionally, we used eQTL summary statistics from GTEx_V8 (https://yanglab.westlake.edu.cn/software/smr/#eQTLsummarydata), which provides information on the association between genetic variants and gene expression levels. SNPs significantly associated with gene expression at a genome-wide significance level (*p* < 5 × 10^-8) were selected as instrumental variables. These SNPs serve as proxies to test for the causal effect of gene expression on the trait. The SMR analysis was performed using the SMR software tool (https://yanglab.westlake.edu.cn/software/smr/#Download), and the HEIDI test was conducted to distinguish between pleiotropy and linkage. Genes with significant SMR *p*-values (*p* < 0.05) and non-significant HEIDI *p*-values (*p* > 0.05) were prioritized for further analysis. These findings contribute to understanding the genetic architecture of IPF and identifying potential therapeutic targets.

### 3.7 Clinical correlation analysis

To explore the expression of core genes, we created violin plots using the “ggviolin” function in the “ggpubr” package to display the differences in expression between groups. To evaluate the predictive ability of the core genes for IPF, we used the “roc” function from the “pROC” package to calculate the AUC values and plot ROC curves. To investigate whether the expression of core genes was linked with the survival time and survival status of IPF patients, we carried out cox proportional hazards regression analysis and survival analysis using the R software’s “survival” and “survminer” packages. We further investigated the relationships between the core genes and age, predicted FVC%, predicted FEV1%, and predicted DLco% using correlation analysis.

### 3.8 Statistical analysis

All statistical analyses were performed using R (version 4.3.1). Wilcoxon test was utilized to compare group differences. Spearman or Pearson correlation analysis was used to explore the relationships between genes, immune cells, and lung function. Cox proportional hazards regression analysis was used to explore the relationship between gene expression and patient survival. Significant is defined to be *P* < 0.05 (“***”, “**”, “*”, “ns” are “*P* < 0.001”“*P* < 0.01”“*P* < 0.05”“no significance”).

## 4 Results

### 4.1 Data processing and telomere-related DEGs screening

**FIGURE 2:**
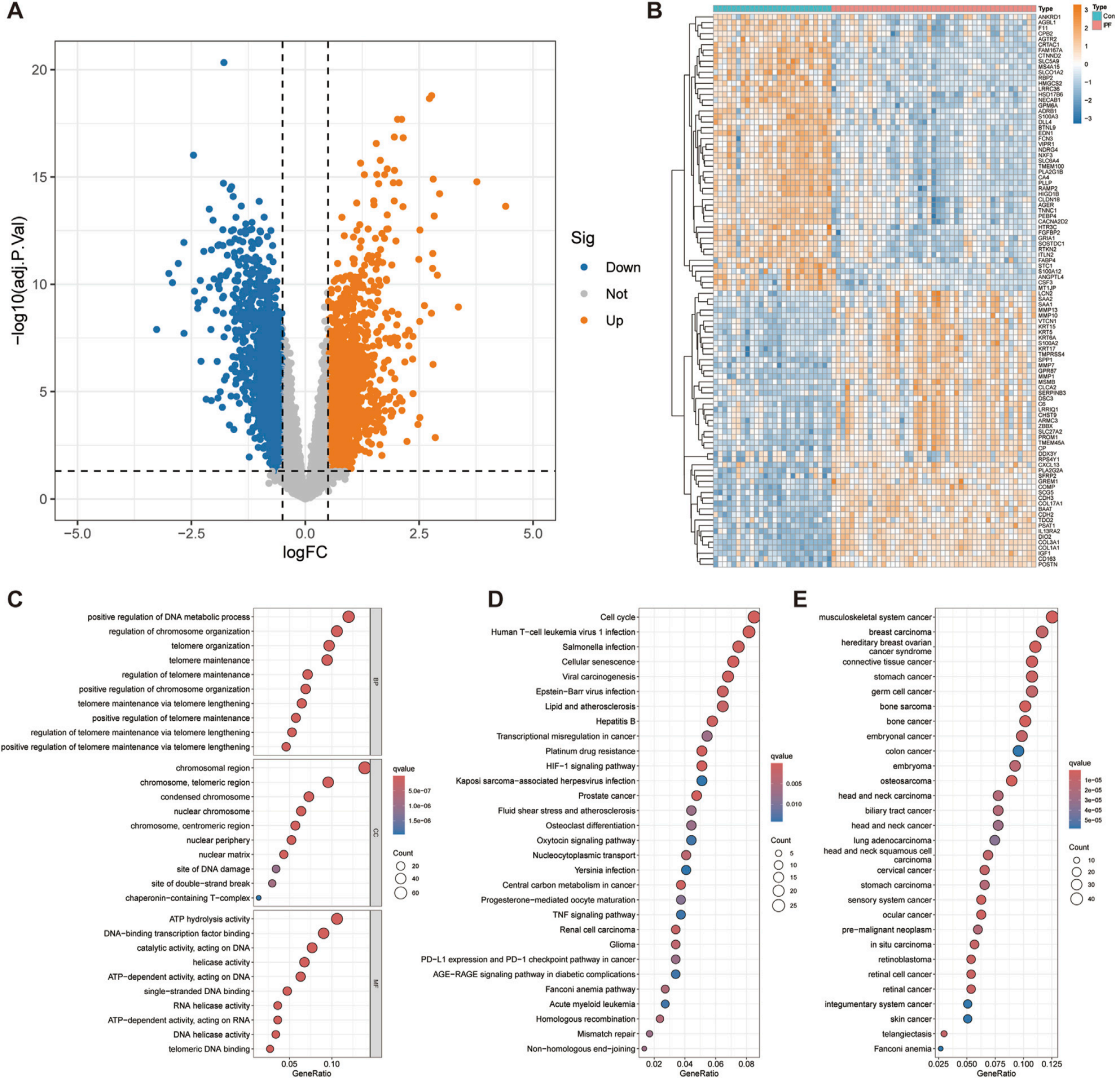
Differential expression analysis and functional enrichment analysis. **(A)** Volcano plot displays 3,844 genes that are differentially expressed. **(B)** The top 50 significantly up- and downregulated genes were displayed on the heatmap. The bubble diagram shows the results of GO **(C)**, KEGG **(D)** and DO **(E)** enrichment analysis.

We combined the GSE10667 and GSE110147 datasets and corrected the batch effect between the datasets for subsequent analysis (Supplementary Figure S1A, B). The volcano plot showed 3,844 DEGs identified through differential analysis, of which 2,097 genes were overexpressed and 1,747 genes were underexpressed (Figure 2A). The heatmap showed the top 50 significantly up and downregulated genes (Figure 2B). After the intersection of DEGs and telomere-related genes, 471 telomere-related DEGs were obtained, of which 308 genes were overexpressed and 163 genes were underexpressed (Supplementary Table S2).

### 4.2 Functional enrichment analysis

To explore the functional roles of telomere-related DEGs, we conducted GO, KEGG, and DO enrichment analyses. GO enrichment analysis revealed several biological processes significantly associated with telomere related DEGs, such as positive regulation of DNA metabolic processes, positive regulation of chromosome organization and positive regulation of telomere maintenance via telomere lengthening (Figure 2C). Cellular component analysis revealed significant enrichment of chromosomal region, nuclear chromosome, nuclear periphery, nuclear matrix, site of DNA damage and site of double − strand break, emphasizing the role of DEGs in DNA repair and nuclear structure. Molecular functional analysis revealed significant enrichment of ATP hydrolysis activity, helicase activity, RNA helicase activity, DNA helicase activity and telomeric DNA binding, indicating that they play a crucial role in DNA and RNA processing. KEGG enrichment analysis further revealed significant enrichment of telomere related DEGs in Cell cycle, Cellular senescence, HIF-1 signaling pathway, Mismatch repair and Non−homologous end−joining (Figure 2D). Other significant enrichment pathways include involvement in various cancers (glioma, renal cell carcinoma, prostate cancer) and infection-related pathways (Kaposi sarcoma−associated herpesvirus infection, Epstein−Barr virus infection, viral carcinogenesis). These indicate that these telomere-related DEGs have a wide range of effects on cell function and disease processes. The DO enrichment analysis revealed significant enrichment of telomere-related DEGs in Fanconi anemia and various malignancies (Figure 2E). This highlights the critical role of telomere-related DEGs in cancer and genetic disorders, emphasizing their potential importance in understanding and targeting these diseases.

### 4.3 Gene module identification and establishment of co-expression network

**FIGURE 3:**
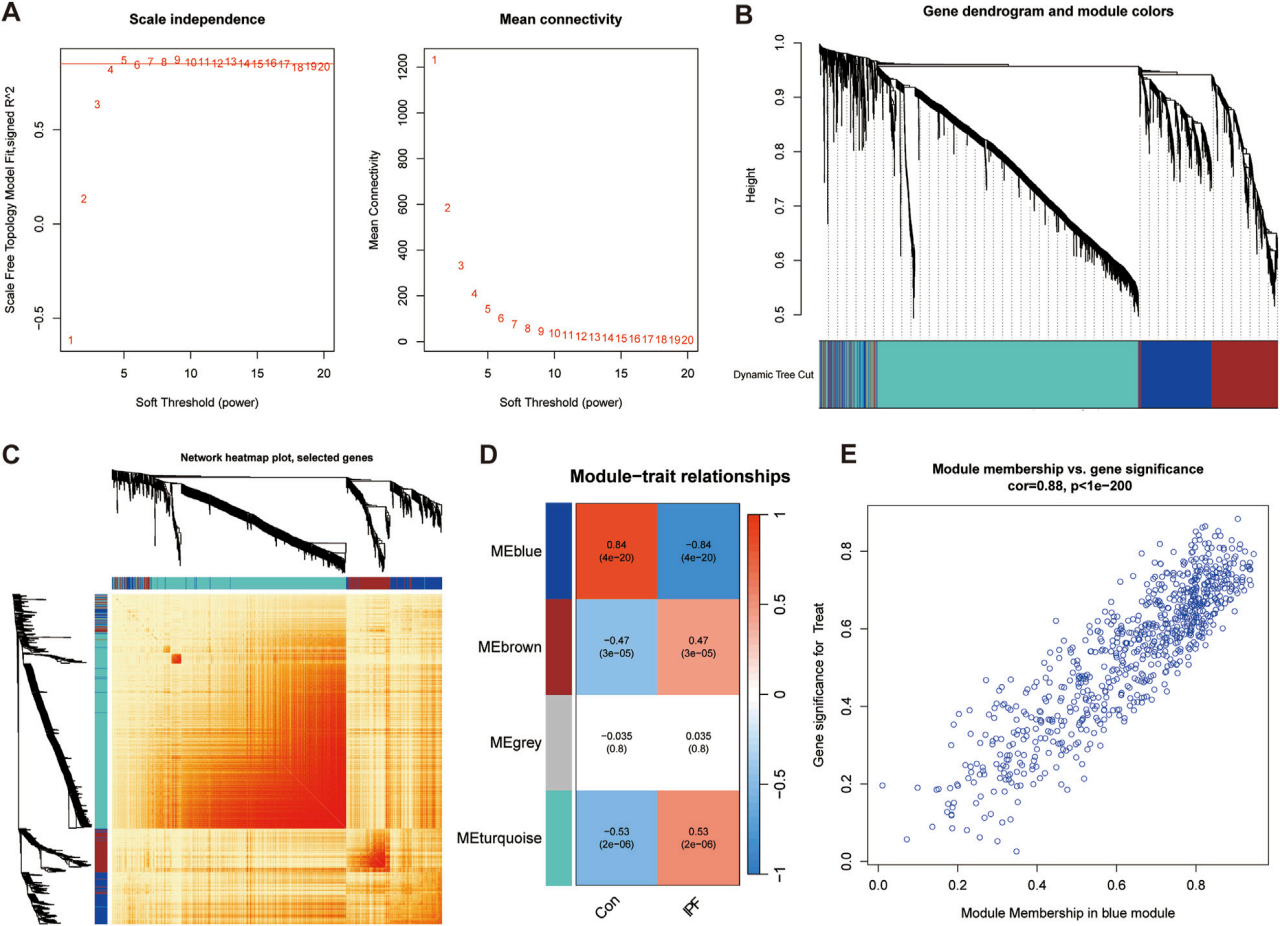
Weighted gene co-expression network analysis (WGCNA). **(A)** Mean connectivity and scale independence of various soft threshold powers. **(B)** Cluster tree dendrogram of co-expression modules. **(C)** Topological overlap matrix (TOM) heatmap among all the modules. **(D)** Heatmap of module-trait correlation. **(E)** MM and GS scatter plot of IPF in blue module.

In order to screen out the hub gene modules related to IPF, we performed a cluster analysis using the WGCNA algorithm on 26 control samples and 45 IPF samples. The clustering tree shows that the samples are well clustered and no obvious outlier samples are detected (Supplementary Figure S2). We selected β = 5 as the most suitable soft threshold parameter for constructing scale-free networks, when R2 is set to 0.85 and the average connectivity is high (Figure 3A). Four co-expression modules in diverse colors were created by the dynamic cutting method (Figure 3B), and a TOM heatmap of all the modules’ associated genes was also supplied (Figure 3C). Subsequently, we plotted the heatmap between these module eigengenes (MEs) and clinical traits (Figure 3D). Turquoise (r = 0.53) and brown (r = 0.47) MEs were significantly positively correlated with IPF, while blue (r = −0.84) MEs was significantly negatively correlated with IPF. The correlation analysis showed that the blue module was highly correlated with IPF (Figure 3E). Finally, we obtained 35 disease candidate genes by taking the intersection of blue module genes and telomere-related DEGs (Table 2).

**TABLE 2:** Detailed information of 35 disease candidate genes.

id	logFC	AveExpr	t	P.Value	adj.P.Val	B
ABCC5	0.9411	9.2126	8.1973	0.0000	0.0000	16.9517
ALPL	−0.6580	8.8712	−3.5933	0.0006	0.0017	−0.9145
BDKRB2	0.6948	9.2600	3.7674	0.0003	0.0011	−0.3731
CAMK2A	−0.7960	5.0719	−5.7440	0.0000	0.0000	6.7613
CCDC155	−0.7611	5.5875	−3.5647	0.0006	0.0018	−1.0020
CCNB1	1.1287	7.2485	11.0169	0.0000	0.0000	28.7959
CDKL2	−0.6226	7.7487	−3.6111	0.0006	0.0016	−0.8599
CLIC3	−1.3823	9.0822	−7.0590	0.0000	0.0000	12.1380
DLG2	0.8595	6.4377	6.8358	0.0000	0.0000	11.2061
ESR2	0.9913	5.7408	6.1519	0.0000	0.0000	8.3950
ETV1	−0.5956	8.5515	−3.3730	0.0012	0.0031	−1.5739
FGFR4	−1.0772	8.0638	−6.4080	0.0000	0.0000	9.4384
FOXR1	−0.6655	4.2482	−4.2590	0.0001	0.0003	1.2454
GAP43	0.9091	4.8057	5.5264	0.0000	0.0000	5.9067
GATA2	−1.2108	8.0871	−7.5189	0.0000	0.0000	14.0740
GLI1	0.6158	7.2788	3.4884	0.0008	0.0023	−1.2322
GPA33	−1.1053	7.1861	−3.6418	0.0005	0.0015	−0.7656
GREM1	2.1971	6.4502	7.0538	0.0000	0.0000	12.1162
HMGB3	1.2436	8.9833	7.4759	0.0000	0.0000	13.8922
HSPA4L	1.8994	6.0461	9.1296	0.0000	0.0000	20.9136
IDO1	−1.1730	8.8754	−3.9202	0.0002	0.0007	0.1162
KCTD16	−1.4901	5.6111	−7.0984	0.0000	0.0000	12.3032
LRRN1	1.4650	6.4372	9.1977	0.0000	0.0000	21.2021
MICA	−1.0703	8.2739	−7.6759	0.0000	0.0000	14.7383
PCP4	1.3468	6.3487	5.7901	0.0000	0.0000	6.9439
PCSK9	−0.8712	7.9531	−3.9670	0.0002	0.0006	0.2686
PDLIM2	−0.8822	9.3993	−7.0138	0.0000	0.0000	11.9489
PNMT	−0.6975	7.7186	−3.2953	0.0015	0.0038	−1.7990
PRX	−1.4277	9.4779	−7.5547	0.0000	0.0000	14.2250
PSAT1	1.9541	7.3432	13.1509	0.0000	0.0000	37.2685
SH3GL2	−1.3732	5.7549	−9.3327	0.0000	0.0000	21.7737
TNNC1	−1.9119	8.9295	−8.5358	0.0000	0.0000	18.3917
UCHL1	0.8241	8.9454	6.1640	0.0000	0.0000	8.4439
WFS1	−1.1707	9.8150	−9.6279	0.0000	0.0000	23.0198
ZSCAN4	1.0096	4.0610	5.5427	0.0000	0.0000	5.9702

### 4.4 Identification of telomere clusters in IPF

**FIGURE 4:**
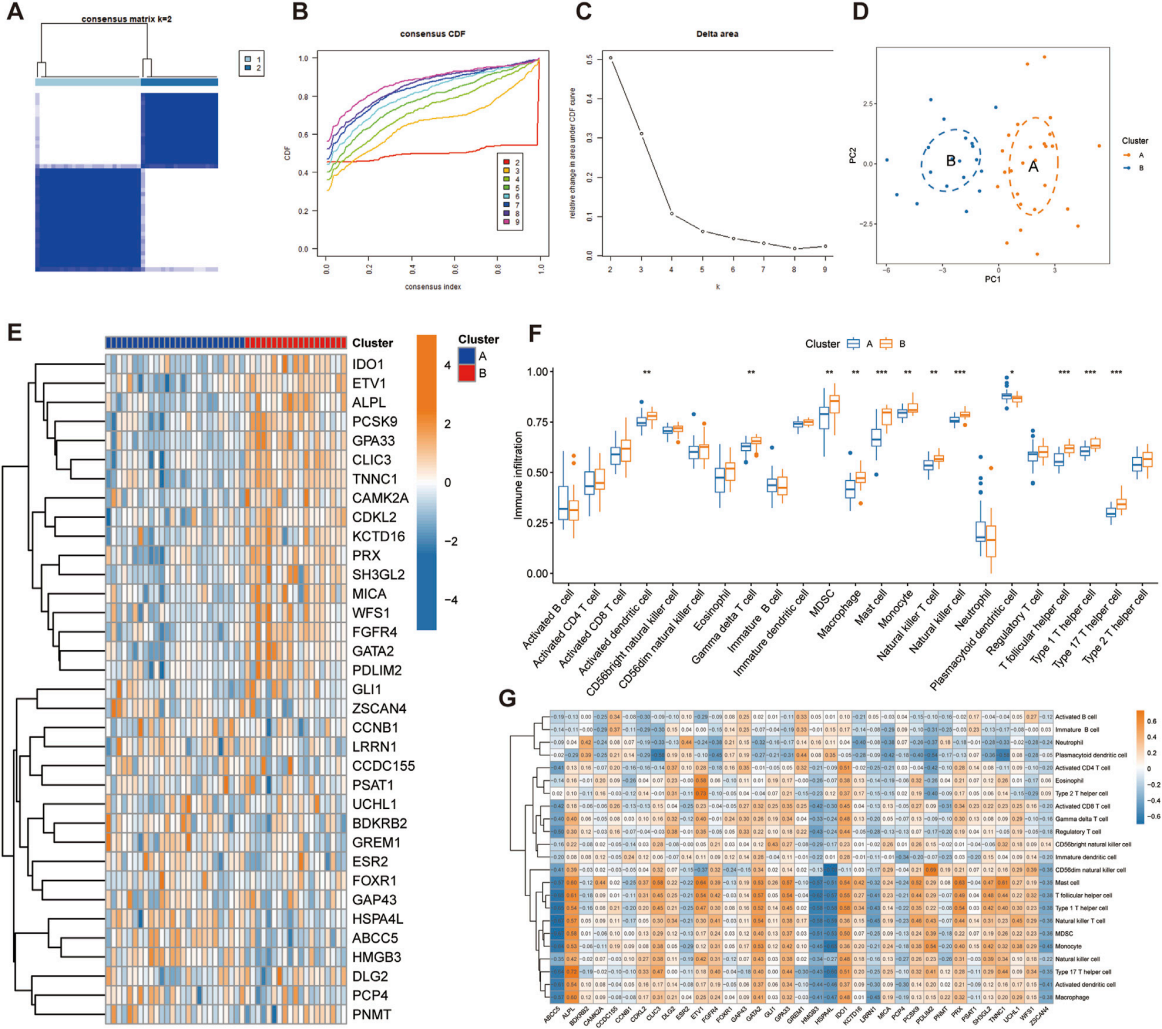
Identification of telomere subtypes in IPF. **(A)** Consensus matrix heatmap when k = 2. **(B)** Consensus cumulative distribution function when k = 2–9. **(C)** Change in the relative area under the CDF curve. **(D)** Significant distinctions across clusters can be seen in the PCA diagram. **(E)** Heatmap shows the expression of 35 disease candidate genes in different clusters. **(G)** The box plot shows the immune infiltration in different clusters. **(H)** Correlation between 35 disease candidate genes and infiltrating immune cells.

Through consensus clustering analysis, we identified two different telomere patterns based on the expression profiles of 35 disease candidate genes, including cluster A (n = 26) and cluster B (n = 19) (Figure 4A). When k = 2–9, the area under the CDF curve shows the difference between the two CDF curves (k and k-1) (Figures 4B, C). The PCA diagram showed significant differences between clusters, and these disease candidate genes can distinguish these two telomere patterns (Figure 4D). The heatmap displayed the differential expression levels of 35 disease candidate genes between clusters A and B (Figure 4E). The results showed that ALPL, CAMK2A, CDKL2, CLIC3, ETV1, FGFR4, GATA2, GPA33, IDO1, KCTD16, MICA, PCSK9, PDLIM2, PRX, SH3GL2, TNNC1 and WFS1 were overexpressed in cluster B, while ABCC5, GREM1, HMGB3 and HSPA4L were opposite. Further immune infiltration analysis revealed that cluster B had a more active immune microenvironment, such as Activated dendritic cell, Gamma delta T cell, MDSC, Macrophage, Mast cell, Monocyte, Natural killer T cell, Natural killer cell, T follicular helper cell, Type 1T helper cell and Type 17T helper cell were significantly upregulated in cluster B (Figure 4F). Finally, we revealed that ALPL, ETV1, GATA2, GPA33, IDO1 and UCHL1 were positively correlated with most immune cells, while ABCC5, HMGB3, HSPA4L, LRRN1 and ZSCAN4 were negatively correlated with most immune cells (Figure 4G).

### 4.5 SMR analysis reveals causal genes of IPF

**FIGURE 5:**
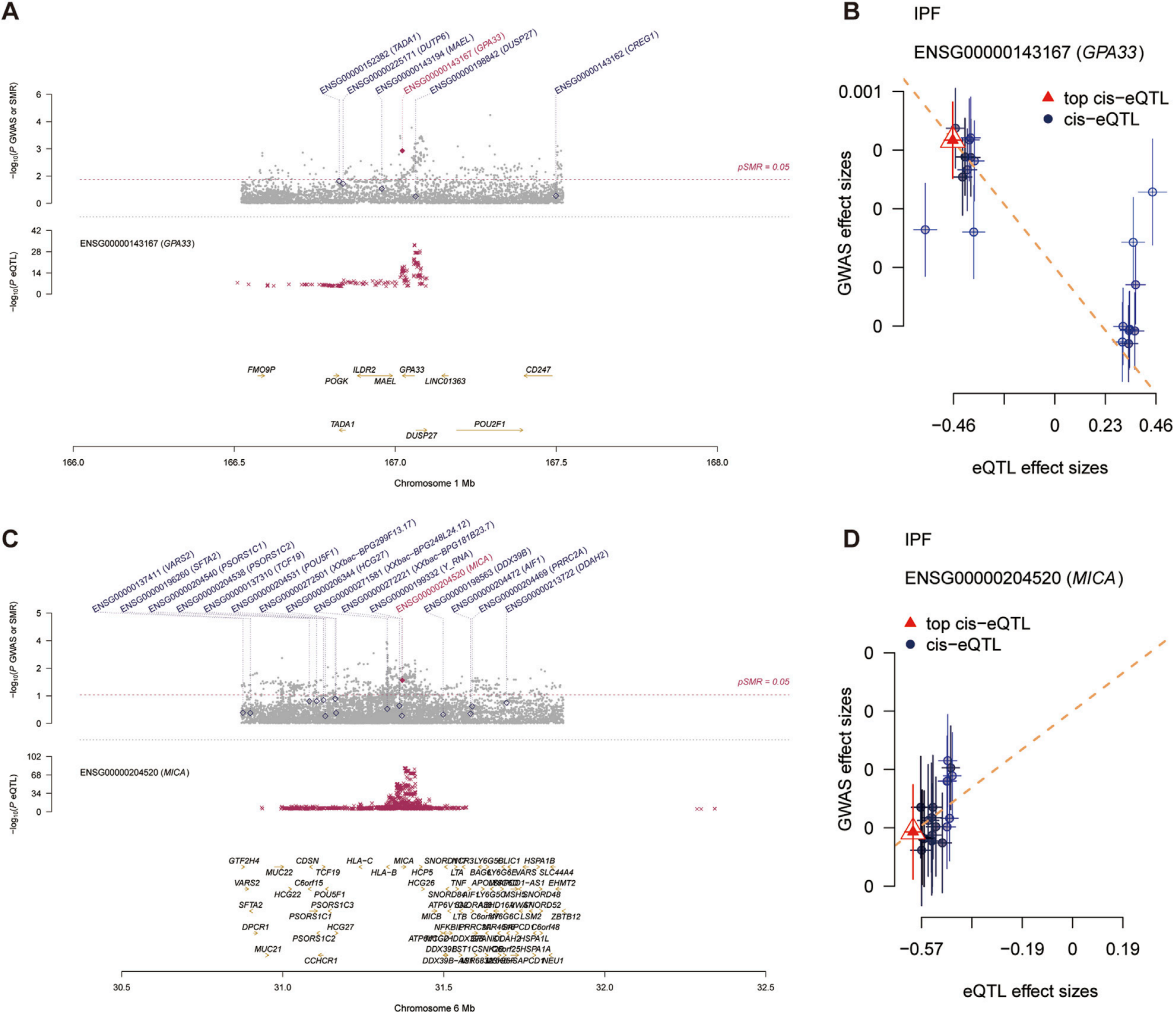
Summary data-based Mendelian randomization (SMR) analysis. **(A)** Prioritizing gene around GPA33 in pleiotropic association with IPF in the participants of European ancestry using GTEx eQTL data. **(B)** The scatter plot illustrates the relationship between the expression site of GPA33 gene and IPF GWAS. **(C)** Prioritizing gene around MICA in pleiotropic association with IPF in the participants of European ancestry using GTEx eQTL data. **(D)** The scatter plot illustrates the relationship between the expression site of MICA gene and IPF GWAS.

In participants of European ancestry, we identified several genes with pleiotropic associations with IPF (Table 3). Specifically, using GTEx eQTL data, our SMR analysis determined two genes with potential causal associations with IPF, including GPA33 (PSMR = 0.0013; PHEIDI = 0.0741) and MICA (PSMR = 0.0112; PHEIDI = 0.9712). Overall, our study revealed that a decrease in GPA33 expression (Figures 5A, B) and an increase in MICA expression (Figures 5C, D) may play a causal role in the pathogenesis of IPF.

**TABLE 3:** Summary data-based Mendelian randomization analysis results.

Gene	topSNP	A1	A2	Freq	b_SMR	se_SMR	p_SMR	p_HEIDI
PCSK9	rs34232196	T	C	0.2336	0.0000	0.0004	0.9578	0.1671
GPA33	rs2281963	C	G	0.5348	−0.0008	0.0003	0.0013	0.0741
LRRN1	rs6442843	C	G	0.8678	0.0000	0.0004	0.9988	0.7178
ABCC5	rs9861983	T	C	0.1869	0.0000	0.0010	0.9687	NA
UCHL1	rs11556271	G	A	0.1163	−0.0005	0.0006	0.3546	0.3964
CAMK2A	rs11744389	C	T	0.3897	0.0007	0.0005	0.1138	0.3581
MICA	rs1051798	T	C	0.3141	0.0006	0.0002	0.0112	0.9712
SH3GL2	rs2754334	T	G	0.9632	−0.0002	0.0006	0.7136	0.8674
ESR2	rs1255986	T	C	0.5408	0.0003	0.0005	0.4523	0.9346
BDKRB2	rs1889373	A	G	0.5258	0.0001	0.0004	0.8540	0.8807
PNMT	rs876493	A	G	0.5805	0.0009	0.0008	0.2592	NA
CCDC155	rs7248502	A	G	0.5239	0.0001	0.0001	0.6800	0.0454

### 4.6 Core genes expression and its association with IPF

**FIGURE 6:**
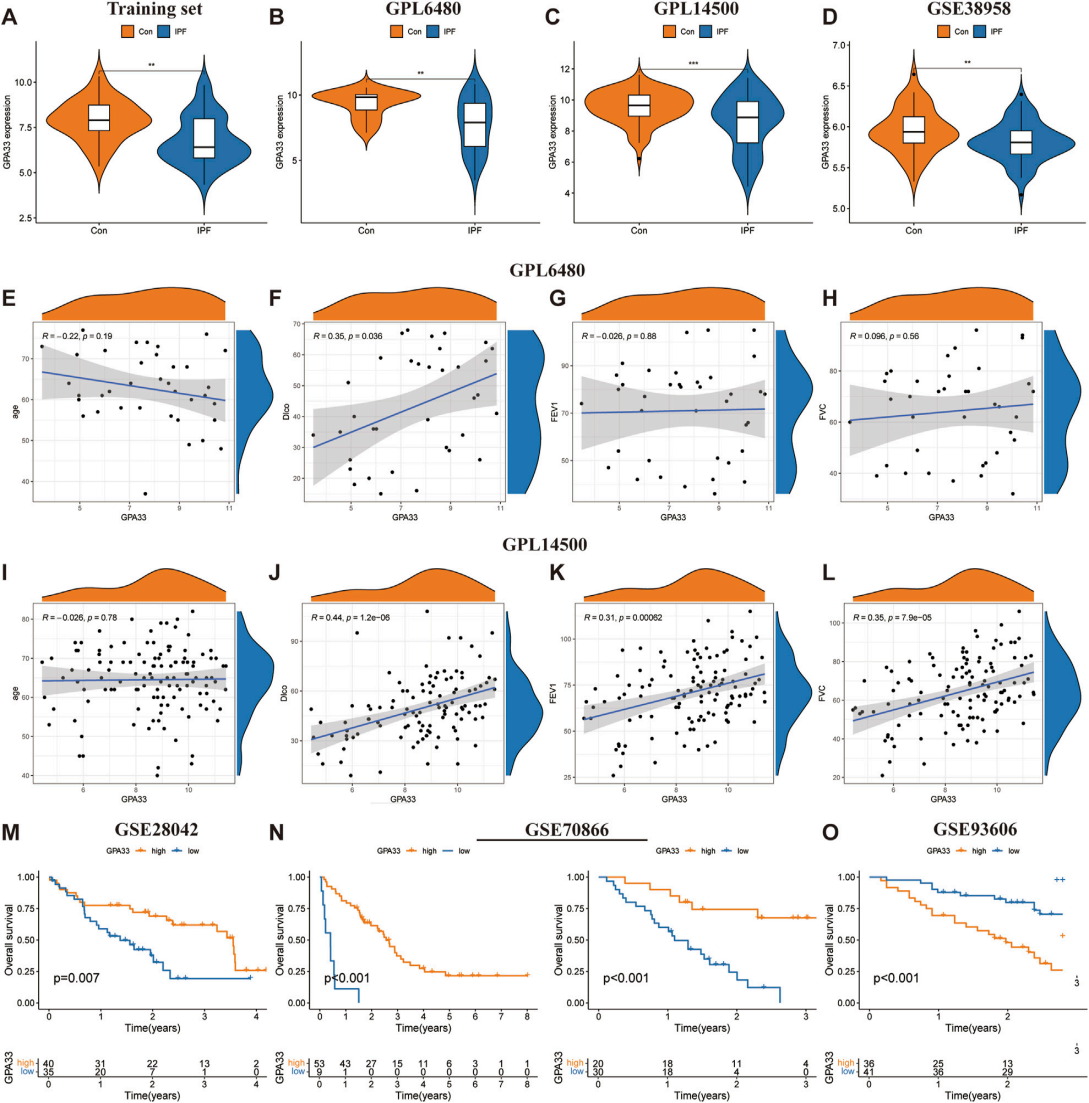
GPA33 expression and clinical correlation analysis. The violin plot shows the expression of GPA33 in the training set **(A)**, GSE47460 dataset GPL6480 platform **(B)**, GSE47460 dataset GPL14500 platform **(C)** and GSE38958 dataset **(D)**. The correlation scatter plot shows the correlation between GPA33 and age **(E)**, DLco % predicated **(F)**, FEV1% predicated **(G)**, FVC % predicated **(H)** in the GSE47460 dataset GPL6480 platform. The correlation scatter plot shows the correlation between GPA33 and age **(I)**, DLco % predicated **(J)**, FEV1% predicated **(K)**, FVC % predicated **(L)** in the GSE47460 dataset GPL14500 platform. **(M)** The survival curve shows the relationship between the expression of GPA33 in GSE28042 dataset and the overall survival of IPF patients. **(N)** The survival curve shows the relationship between the expression of GPA33 and the overall survival of IPF patients in the FREIBURG and SIENA cohorts of the GSE70866 dataset. **(O)** The survival curve shows the relationship between the expression of GPA33 in GSE93606 dataset and the overall survival of IPF patients.

To assess the predictive ability of core genes for IPF, we plotted ROC curves for the core genes predicting the occurrence of IPF. In the training set, GPA33 (AUC = 0.730) and MICA (AUC = 0.889) showed good predictive ability for IPF (Supplementary Figure S3A). To evaluate the stability of core gene expression in IPF, we verified it on multiple external datasets. In the training set, GPA33 and MICA were significantly downregulated in IPF (Figure 6A; Supplementary Figure S3B). In the GPL6480 platform and GPL14500 platform of the GSE47460 dataset, we observed that GPA33 was significantly downregulated in IPF (Figures 6B, C). In the GSE32537 dataset, we found that GPA33 and MICA were lowly expressed in IPF, but GPA33 expression was not significant (Supplementary Figure S3B). We further verified the expression of core genes in peripheral blood tissues. In the GSE38958 dataset, we found that GPA33 was significantly downregulated in IPF (Figure 6D). In the GSE93606 dataset, we found that GPA33 and MICA were upregulated in IPF, but MICA expression was not significant (Supplementary Figure S3D).

We further revealed the relationship between GPA33 expression and lung function. In the GPL6480 platform, we found that GPA33 expression was positively correlated with predictive DLco% (R = 0.35, *p* = 0.036) (Figures 6E–H). In the GPL14500 platform, we found that GPA33 expression was positively correlated with predicted DLco% (R = 0.44, *p* = 1.2e − 06), predicted FEV1% (R = 0.31, *p* = 0.00062), and predicted FVC% (R = 0.35, *p* = 7.9e − 05) (Figures 6I–L).

Moreover, survival analysis revealed that the expression of core genes was related to the survival time of IPF patients (Table 4). In the GSE28042 dataset, we found that GPA33 is a protective gene for IPF, and its high expression is associated with longer survival of patients (Figure 6M; Supplementary Figure S3E). In the FREIBURG cohort of the GSE70866 dataset, we found that GPA33 and MICA are protective genes for IPF, and their high expression is associated with longer survival of patients (Figure 6N; Supplementary Figure S3F). In the SIENA cohort of the GSE70866 dataset, we found that GPA33 is a protective gene for IPF, and its high expression is associated with longer survival of patients (Figure 6N; Supplementary Figure S3F). However, in the GSE93606 dataset, we found that GPA33 is a risk gene for IPF, and its high expression is associated with shorter survival of patients (Figure 6O; Supplementary Figure S3G).

**TABLE 4:** Survival analysis results of core genes.

Dataset	Gene	HR	HR.95L	HR.95H	P-value	km
GSE28042	GPA33	0.4550	0.2364	0.8755	0.0184	0.0074
	MICA	0.8157	0.4157	1.6004	0.5535	0.0811
GSE70866(FREIBURG)	GPA33	0.6032	0.4635	0.7848	0.0002	0.0000
	MICA	0.5558	0.2044	1.5117	0.2500	0.0002
GSE70866(SIENA)	GPA33	0.5998	0.4548	0.7911	0.0003	0.0002
	MICA	0.7260	0.3633	1.4506	0.3645	0.1208
GSE93606	GPA33	2.8303	1.3216	6.0613	0.0074	0.0002
	MICA	0.4539	0.0632	3.2619	0.4325	0.0595

### 4.7 Changes of IPF immune microenvironment

**FIGURE 7:**
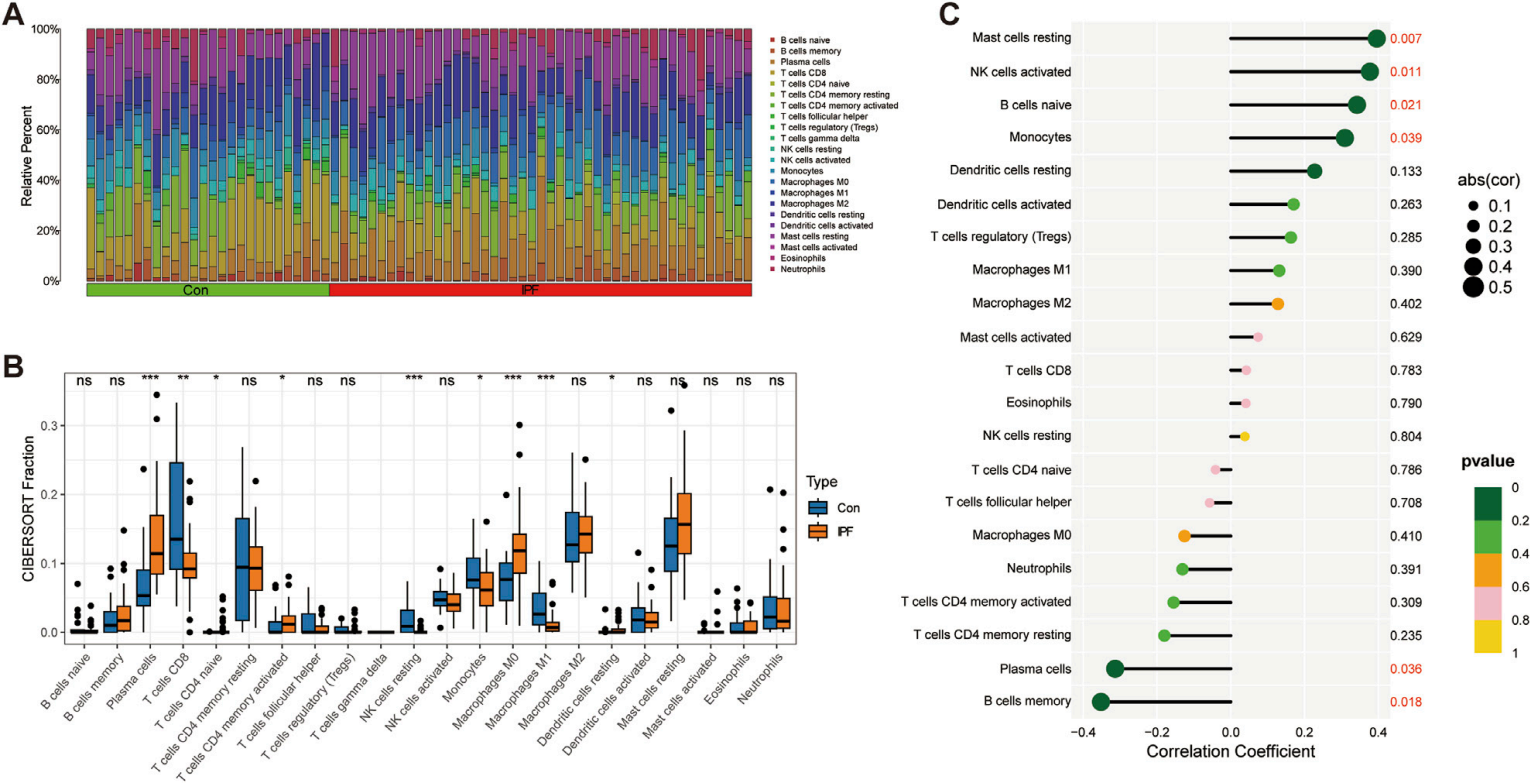
Immune infiltration analysis. **(A)** The bar chart shows the relative proportions of 22 immune cells in the control group and IPF group. **(B)** The box plot shows the expression of 22 immune cells in the control group and IPF group. **(C)** The correlation lollipop plot shows the relationship between GPA33 and immune cells.

Immune infiltration analysis revealed the changes in the proportion of immune cells among samples (Figure 7A). We further revealed that Plasma cells, T cells CD4 memory activated, Macrophages M0, and Dendritic cells resting were significantly upregulated in IPF, while T cells CD8, NK cells resting, Monocytes, and Macrophages M1 were significantly downregulated in IPF (Figure 7B). Finally, we revealed the relationship between GPA33 expression and immune cells. We found that GPA33 expression was positively correlated with Mast cells resting, NK cells activated, B cells naive and Monocytes, but negatively correlated with Plasma cells and B cells memory (Figure 7C).

## 5 Discussion

In this study, 471 telomere-related DEGs were screened from the combined dataset, including 308 upregulated genes and 163 downregulated genes. Through functional enrichment analysis, it was found that these telomere-related DEGs were involved in DNA metabolic processes, chromosome organization, telomere maintenance, RNA helicase activity, DNA helicase activity, telomeric DNA binding, Cell cycle, Cellular senescence, HIF-1 signaling pathway, Mismatch repair and other biological processes. WGCNA was used to screen co-expressed gene modules and obtain disease candidate genes. Then, we used consensus clustering analysis to identify two different telomere patterns, including cluster A (n = 26) and cluster B (n = 19). Immune infiltration analysis showed that cluster B had a more active immune microenvironment, indicating its potential association with IPF. Through SMR analysis, we screened two genes (GPA33 and MICA) that are causally related to IPF. Further analysis showed that GPA33 had a stable expression pattern in IPF, and high expression of GPA33 was associated with longer overall survival, higher predicted FVC %, higher predicted FEV1% and higher predicted Dlco % in IPF patients.

Telomere genes were found to be important in the regulation of DNA metabolism, cell cycle, cellular senescence, chromosome tissue, and other biological activities, according to enrichment analysis. Premature lung failure and fibrosis in IPF are caused by DNA damage in telomerase-deficient lungs. Existing data show that telomere dysfunction leads to pulmonary fibrosis by changing the lung microenvironment and cell conduction to cause alveolar stem cell senescence (Zhang et al., 2021; Alder and Armanios, 2022). Studies have found that pulmonary fibrosis is closely related to telomere shortening and increased DNA damage (van Batenburg et al., 2021; McDonough et al., 2018). In the process of pulmonary fibrosis, AEC2 stem cells are vulnerable to pressure attacks, triggering telomere DDR and senescence-related directional differentiation disorders (Hong et al., 2022). Senescent cells are metabolically active, producing various cytokines, chemokines, growth factors and matrix metalloproteinases, forming an senescence-associated secretory phenotypes (SASP) (Pawlikowski et al., 2013; Freund et al., 2010). Studies have found that adjacent cells are also affected by TGF-β secreted by senescent cells and enter the aging state (Acosta et al., 2013). In particular, telomere shortening combined with environment-induced lung injury accelerates the progression of pulmonary fibrosis by stimulating the TGF-β/Smads signaling pathway (Liu et al., 2018). It is believed that once AEC2 stem cells enter replicative senescence, they will be entangled in the environment of SALI, and carry out two-way signal transduction with senescent cells through various inflammatory factors, thus promoting the development of fibrosis (Chen et al., 2015; Kadota et al., 2020; Misawa et al., 2020). In addition, mouse models showed that conditional knockout of Cdc42 in AEC2s could lead to progressive pulmonary fibrosis. This suggests that Cdc42 deficiency impairs the ability of AEC2s to differentiate into AEC1s, thereby inhibiting the formation of new alveoli and leading to increased pulmonary mechanical tension (Wu et al., 2020). Other studies have shown that radiation exposure, oxidative stress, or bleomycin can trigger the degradation of TPP1 in AEC2s, triggering telomere unblocking, stem cell failure, DDR, fiber gene expression and pulmonary fibrosis (Wang et al., 2020). Interestingly, TELODIN prevents pulmonary fibrosis by competitively inhibiting pressure-induced TPP1 to accelerate turnover and telomere shortening (Wang et al., 2020; Wang et al., 2021). Therefore, the development of some new treatment strategies for telomere dysfunction is expected to become a new way to intervene in pulmonary fibrosis. It is worth mentioning that studies have shown that senescent fibroblasts in IPF patients are abnormally activated, accompanied by SASP, anti-apoptosis, telomere shortening, etc. These senescent fibroblasts’ traits in IPF patients mostly contribute to the onset and progression of the disease (Lin and Xu, 2020). In general, on the one hand, the senescence of AEC2s and fibroblasts accelerates the progression of pulmonary fibrosis by promoting the production of SASP. On the other hand, during epithelial injury repair, AEC2 stem cells cannot differentiate into AEC1 cells, resulting in increased lung mechanical tension and activation of TGF-β signaling pathway in AEC2 to promote pulmonary fibrosis.

At present, more and more evidence shows that immune dysfunction may be an important part of the pathogenesis of IPF. In innate immunity, transgenic mice with telomere dysfunction showed infiltration of monocytes, macrophages, neutrophils, lymphocytes and natural killer cells in the lungs (Naikawadi et al., 2016; Liu et al., 2018; Chen et al., 2015). It has been reported that elevated levels of IL-8 and granulocyte colony-stimulating factor in bronchoalveolar lavage fluid from IPF patients are linked to an increase in activated neutrophils, a risk of acute exacerbations, and a decline in lung function (Papiris et al., 2018). M2 macrophages play a crucial part in the pathogenesis of pulmonary fibrosis by secreting a number of growth factors to promote the deterioration of pulmonary fibrosis (Heukels et al., 2019; Wynn et al., 2013). Increased expression of prefibrotic genes (arginase1 and MMP13) was observed in monocytes-derived macrophages from IPF patients and experimental pulmonary fibrosis (Misharin et al., 2017). However, how telomere dysfunction causes the infiltration of innate immune cells in the lung and how it participates in the occurrence of pulmonary fibrosis require further study. In adaptive immunity, the pathogenesis of IPF, in which a Th1/Th2 imbalance response contributes to pulmonary fibrosis, may involve dysfunction amongst Th subsets. Previous studies have confirmed that Th2 cytokines play a leading role in pulmonary fibrosis, and its pathogenesis is related to the secretion of IL-4, IL-5 and IL-13 (Chung et al., 2016; Wynn, 2015; Harari and Caminati, 2010). Th1 is mainly related to the production of pro-inflammatory cytokine IFN-γ, and its secretion is reduced in pulmonary interstitial fibrosis and rat pulmonary fibrosis models (Vu et al., 2019; Maeyama et al., 2020). This suggests that impaired IFN-γ release may contribute to the development of pulmonary fibrosis. Other studies have demonstrated that the Th1/Th2 imbalance is only one aspect of the pathogenesis of IPF and that its more intricate interactions with other T cells (such as Tregs, Tfhs, NKTs, and T cells) may be more significant in the pathogenesis of IPF (Spagnolo et al., 2022). By promoting the production of TGF-β in the early stages of IPF, Tregs play a pro-fibrotic role, whereas in the late stages, Tregs play a protective role by stimulating the secretion of IL-10 (Boveda-Ruiz et al., 2013). In our study, two telomere patterns (cluster A and cluster B) were identified from 35 important telomere-related genes by consensus clustering analysis. Cluster B has a more active immune microenvironment, indicating that cluster B may be related to IPF. Our results show that cluster B has higher levels of Th1 and Th2 cells, indicating that more complex interactions between various T cells may be crucial in the pathogenesis of IPF. Although the mechanism of adaptive immunity in IPF remains unclear, the interaction between various T cell populations and how telomere dysfunction causes adaptive immune cells to infiltrate the lungs is a promising research area that may help identify possible molecular therapeutic targets.

Our study first revealed the expression of GPA33 in IPF and its potential protective effect. Our results showed that GPA33 was significantly downregulated in IPF patients, and high expression of GPA33 was associated with longer survival and higher lung function indicators in IPF patients. The consistency of GPA33 expression was verified in lung tissue and peripheral blood samples. However, in another independent peripheral blood sample, we observed that GPA33 was significantly highly expressed in IPF. In the GSE93606 dataset, we observed that high GPA33 expression is associated with shorter survival in IPF patients, which appears to contradict the results from other datasets. First, gene expression in peripheral blood samples may differ significantly from that in lung tissue samples. Although peripheral blood samples are easier to collect, their gene expression profiles may not fully reflect the situation in lung tissue. Second, variations in the technical platforms and data processing methods used across different datasets could affect the gene expression results. Additionally, biological heterogeneity between samples may also contribute to the observed discrepancies. Our study aims to explore novel therapeutic targets for IPF. Despite the current findings showing differences in GPA33 expression across datasets, these results lay the groundwork for further in-depth research. Future studies should increase the sample size, conduct multi-center validation, and explore the specific mechanisms of GPA33 in IPF to further confirm its potential as a therapeutic target. Furthermore, we found that MICA was a risk gene for IPF through SMR analysis, but further analysis found that MICA was significantly downregulated in IPF. Previous studies have found that MICA is expressed in alveolar epithelial cells and fibroblasts of IPF patients (Aquino-Galvez et al., 2009). This indicates that the abnormal expression of MICA in IPF patients may be closely related to the pathological process of the disease. While MICA is considered a risk gene for IPF, our study consistently found it to be downregulated in IPF lung tissue across three independent datasets: GSE2052, GSE24206, and GSE53845 (Supplementary Figure S4). Specifically, MICA was downregulated in the GSE2052 and GSE53845 datasets, although the downregulation in GSE53845 was not statistically significant. In the GSE24206 dataset, we found that MICA was downregulated in both the early and advanced stages of IPF, with more pronounced downregulation in the advanced stage. This phenomenon may reflect the complex role of MICA in disease progression, such as potentially promoting fibrosis by suppressing immune cell activity. Although the downregulation was not significant in some datasets, the overall trend clearly indicates low MICA expression in IPF, suggesting a potential suppressive regulatory role in the pathogenesis of IPF. We also attempted to explore the causal relationship between MICA and IPF through Mendelian randomization, but were unable to perform multiple validations due to limited lung tissue-specific eQTL data. Nevertheless, based on the consistent results across multiple datasets, we believe that the low expression of MICA in IPF is reliable, and future experimental studies will further investigate its specific mechanisms. In summary, our research provides an important foundation for understanding the potential function of MICA in IPF and points to future directions for further investigation.

In addition, immune infiltration analysis revealed a downregulation of NK cells and an upregulation of plasma cells and memory B cells in IPF. The decrease in NK cell numbers may indicate an impaired ability of IPF patients to eliminate diseased cells, contributing to disease progression and exacerbation. Previous studies have reported reduced proportion and activity of NK cells in the lungs of IPF patients (Cruz et al., 2021). Another study indicated that the lung microenvironment in advanced IPF impairs NK cell activity, thereby reducing the clearance of senescent cells and hindering the reversal of lung fibrosis (Cruz et al., 2023). Conversely, the upregulation of plasma cells and memory B cells suggests a significant role for B cell-mediated immune responses in IPF. Prior research found that bortezomib treatment in mice depletes plasma cells, thereby reducing bleomycin-induced lung fibrosis (Prêle et al., 2022). Further correlation analysis revealed that GPA33 expression is positively correlated with NK cells but negatively correlated with plasma cells and memory B cells. These findings suggest that GPA33 may play a crucial role in regulating the function and distribution of different immune cell types. Although research on GPA33 and MICA in IPF is limited, studies on these genes in other diseases provide valuable insights. Given that GPA33 plays a crucial role in epithelial adhesion and cell proliferation and differentiation in the intestinal epithelium, and MICA is a ligand for the NKG2D receptor, which is closely related to NK cell activation (Xing and Ferrari de Andrade, 2020), we can infer their potential roles in the context of telomere dysfunction. Specifically, GPA33 may impact the renewal and repair processes of epithelial cells under conditions of telomere dysfunction, while MICA may influence immune surveillance mechanisms and play a role in the progression of IPF. Therefore, we hypothesize that the reduced expression of MICA in the context of telomere dysfunction might weaken NK cell activity, leading to reduced clearance of senescent cells and thereby promoting fibrosis development.

Our results revealed a positive correlation between the expression of GPA33 and lung function indicators. An increasing number of studies have confirmed that decreased lung function is a feature of disease progression and poor prognosis in patients with IPF, and decreased FVC and Dlco can predict the risk of death in patients with IPF (Brown et al., 2020; Khan et al., 2022; Qiu et al., 2018; Ley et al., 2012). A multicenter observational retrospective study showed that individuals with maintenance of FVC but a moderate-to-severe DLco reduction and a UIP radiological pattern at diagnosis are at greater risk of progression, death, or requiring lung transplantation (Bermudo et al., 2022). To delve deeper into these findings, future research could further explore the exact mechanistic of GPA33 gene in the development and progression of IPF. In addition, considering the association between declining lung function and adverse outcomes in IPF patients, our study provides robust support for GPA33 gene as a potential diagnostic and prognostic biomarker for IPF. Further clinical and molecular investigations are needed to validate their effectiveness as biological markers for IPF and explore new avenues for patient management and therapeutic strategies.

However, our research also has some limitations. First, the mechanism of GPA33 in IPF remains unclear. Secondly, considering that our work is based on bioinformatics analysis, the effectiveness needs to be confirmed in further animal and human experimental studies. In addition, our study relies on publicly available data sets, and differences in sample collection, processing, and sequencing methods between different data sets may introduce variation. In the future research, we will continue to focus on the role of GPA33 in IPF, and through experimental verification to enhance the reliability and applicability of the research results.

## 6 Conclusion

In summary, our study provides a new perspective on the role of telomere dysfunction and immune infiltration in IPF. Our study supports GPA33 as a potential therapeutic target for IPF, and further research may reveal how GPA33 affects cell function and disease progression.

## Data Availability

Publicly available datasets were analyzed in this study. This data can be found here: GEO database (https://www.ncbi.nlm.nih.gov/geo/) with accession numbers GSE10667, GSE110147, GSE47460, GSE32537, GSE38958, GSE28042, GSE93606, GSE70866; TelNet database (http://www.cancertelsys.org/telnet/); GWAS Catalog database (https://www.ebi.ac.uk/gwas/home) with accession number GCST90018120; GTEx_V8 (https://yanglab.westlake.edu.cn/software/smr/#eQTLsummarydata).
