# Supplementary material for: Role of telomere dysfunction and immune infiltration in idiopathic pulmonary fibrosis: new insights from bioinformatics analysis
Source: Front Genet. 2024 Sep 13;15:1447296. doi: 10.3389/fgene.2024.1447296 (PMC11427275; doi:10.3389/fgene.2024.1447296)
Supplement: Supplementary file 1 [file Table1.DOCX]

**
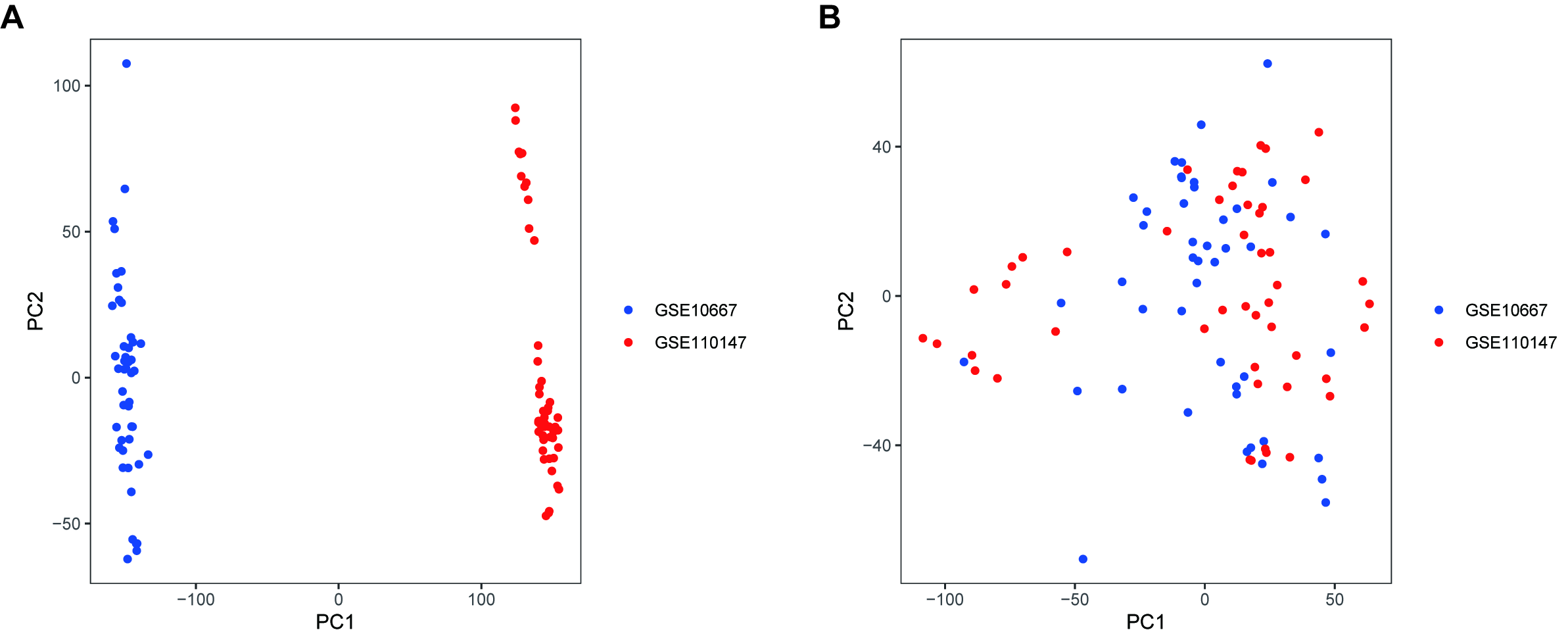
**

**Supplementary Figure 1**. PCA analysis before and after batch correction. (A) The dataset before batch correction is shown in the PCA graphic. (B) Dataset after batch correction is displayed in the PCA plot.


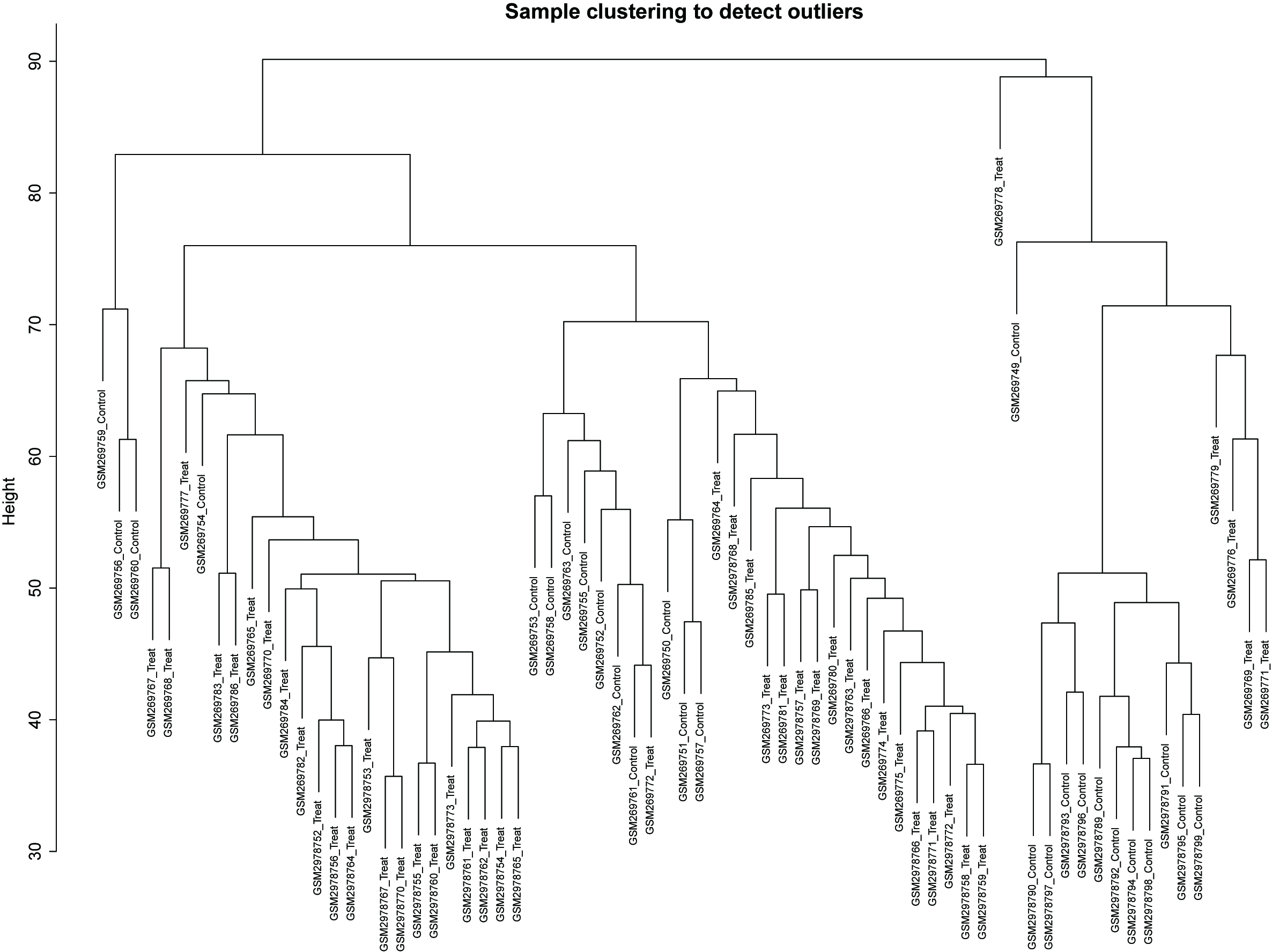


**Supplementary Figure 2**. The clustering tree diagram shows the clustering results of the samples.


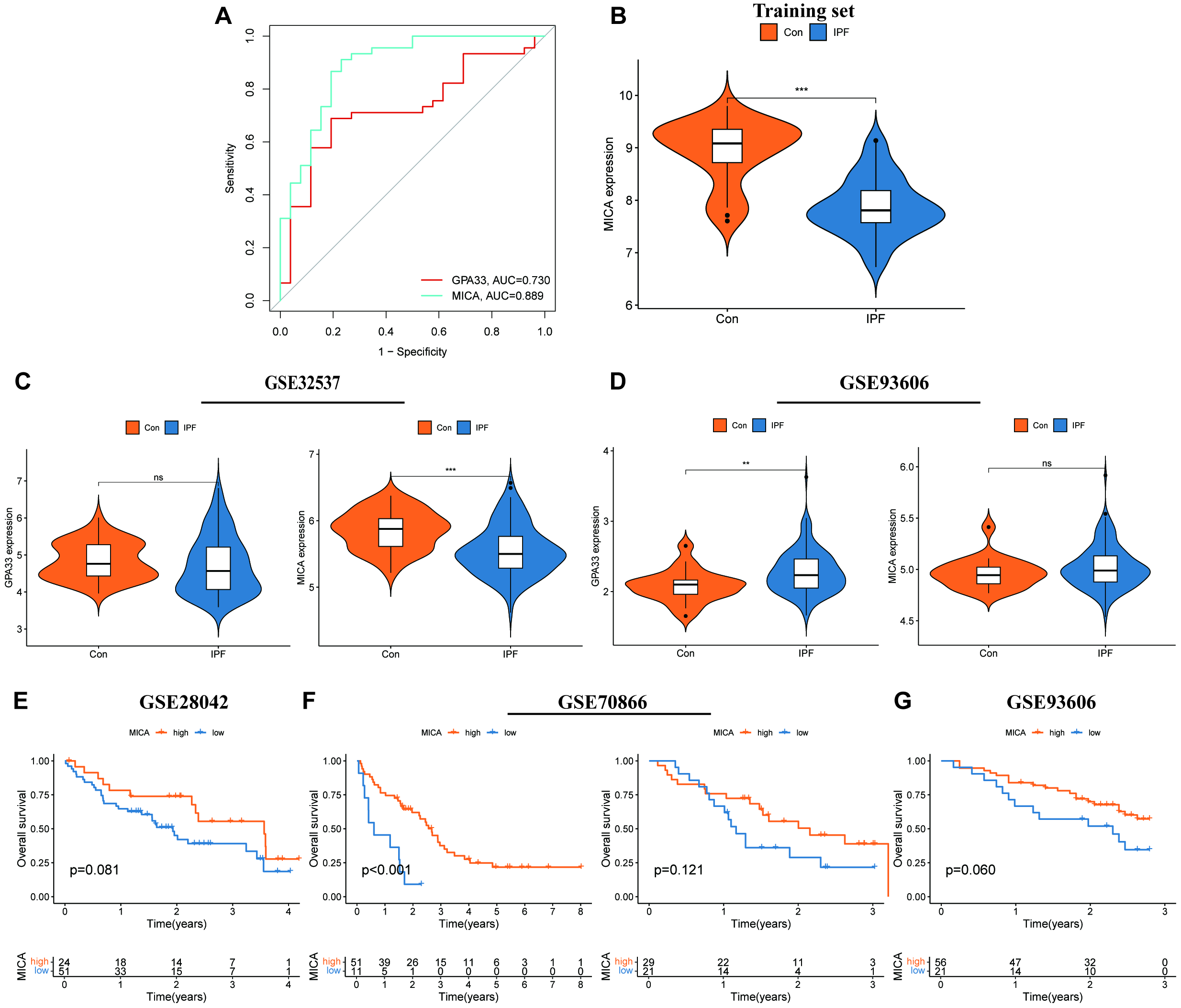


**Supplementary Figure 3**. **Core genes expression and clinical correlation analysis**. (A) The ROC curve shows the ability of core genes to predict IPF in the training set. (B) The violin plot shows the expression of MICA in the training set. The violin plot shows the expression of the core genes in the GSE32537 (C) and GSE93606 (D) datasets. (E) The survival curve shows the relationship between the expression of MICA in GSE28042 dataset and the overall survival of IPF patients. (F) The survival curve shows the relationship between the expression of MICA and the overall survival of IPF patients in the FREIBURG and SIENA cohorts of the GSE70866 dataset. (G) The survival curve shows the relationship between the expression of MICA in GSE93606 dataset and the overall survival of IPF patients.


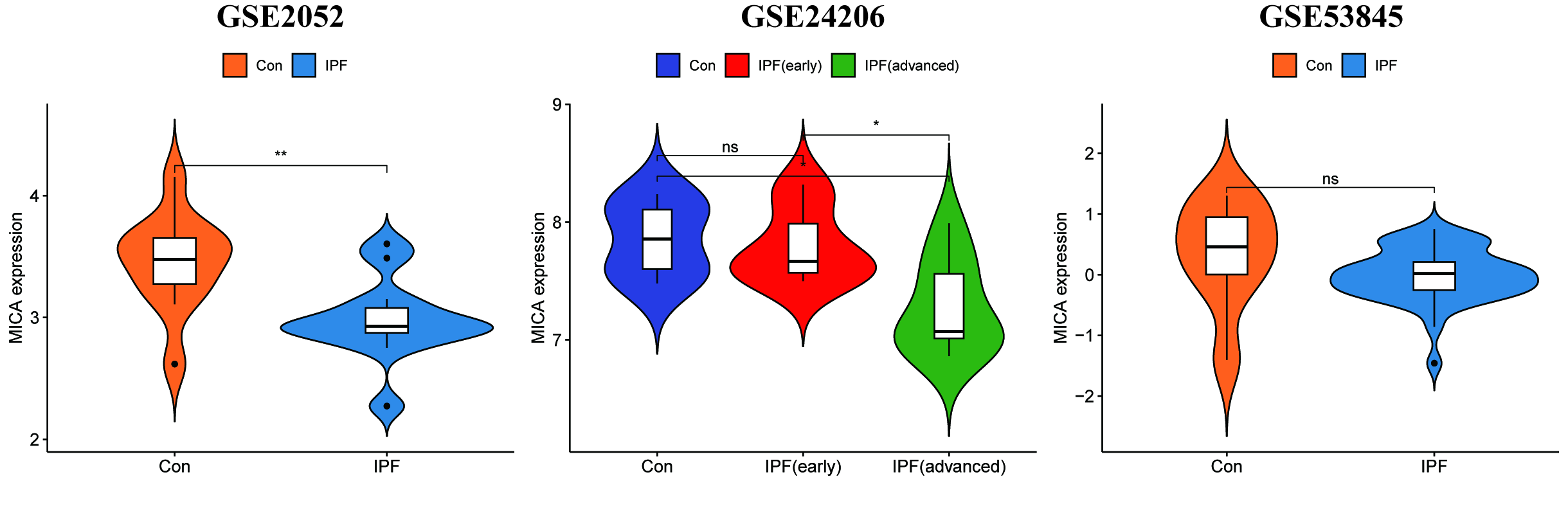


**Supplementary Figure 4**. The expression of MICA in the GSE2052, GSE24206, and GSE53845 datasets.

Supplementary Table 1 | Telomere related genes.

| H. sapiens (human) | ASF1A | Chromatin organization |
| --- | --- | --- |
| H. sapiens (human) | ASF1B | Chromatin organization |
| H. sapiens (human) | ATM | DNA replication and repair |
| H. sapiens (human) | ATR | DNA replication and repair |
| H. sapiens (human) | ATRX | Chromatin organization |
| H. sapiens (human) | BLM | DNA replication and repair |
| H. sapiens (human) | CBX3 | Chromatin organization |
| H. sapiens (human) | DAXX | Chromatin organization |
| H. sapiens (human) | ERCC4 | DNA replication and repair |
| H. sapiens (human) | FANCD2 | DNA replication and repair |
| H. sapiens (human) | FEN1 | DNA replication and repair |
| H. sapiens (human) | H3F3A | Chromatin organization |
| H. sapiens (human) | HMBOX1 | Telomere biology |
| H. sapiens (human) | NSMCE2 | Protein modification |
| H. sapiens (human) | PCNA | Cell cycle |
| H. sapiens (human) | PIN1 | Protein modification |
| H. sapiens (human) | PINX1 | Telomere biology |
| H. sapiens (human) | PML | Nuclear organization |
| H. sapiens (human) | POT1 | Telomere biology |
| H. sapiens (human) | RPA1 | DNA replication and repair |
| H. sapiens (human) | RPA2 | DNA replication and repair |
| H. sapiens (human) | RTEL1 | DNA replication and repair |
| H. sapiens (human) | SP100 | Nuclear organization |
| H. sapiens (human) | SUMO1 | Protein modification |
| H. sapiens (human) | SUMO2 | Protein modification |
| H. sapiens (human) | TERF1 | Telomere biology |
| H. sapiens (human) | TERF2 | Telomere biology |
| H. sapiens (human) | TERF2IP | Telomere biology |
| H. sapiens (human) | TERRA | Telomere biology |
| H. sapiens (human) | TERT | Telomere biology |
| H. sapiens (human) | TINF2 | Telomere biology |
| H. sapiens (human) | TOP3A | DNA replication and repair |
| H. sapiens (human) | UBE2I | Protein modification |
| H. sapiens (human) | XRCC6 | DNA replication and repair |
| H. sapiens (human) | CDKN1A | Cell cycle |
| H. sapiens (human) | DNMT1 | Chromatin organization |
| H. sapiens (human) | HDAC7 | Chromatin organization |
| H. sapiens (human) | HMGN5 | RNA transcription and processing |
| H. sapiens (human) | PARP2 | DNA replication and repair |
| H. sapiens (human) | SENP6 | Protein modification |
| H. sapiens (human) | TEP1 | Telomere biology |
| H. sapiens (human) | AASDHPPT | Metabolism |
| H. sapiens (human) | ACP1 | Protein modification |
| H. sapiens (human) | ACTR5 | DNA replication and repair |
| H. sapiens (human) | ACTR8 | DNA replication and repair |
| H. sapiens (human) | ALYREF | RNA transcription and processing |
| H. sapiens (human) | SLF1 | Cell cycle |
| H. sapiens (human) | ANP32B | Cell differentiation |
| H. sapiens (human) | ANP32E | Nucleocytoplasmic transport |
| H. sapiens (human) | APEX1 | DNA replication and repair |
| H. sapiens (human) | API5 | Cell death |
| H. sapiens (human) | APLF | DNA replication and repair |
| H. sapiens (human) | ARF3 | Protein transport |
| H. sapiens (human) | ARF4 | Protein transport |
| H. sapiens (human) | ARF5 | Protein transport |
| H. sapiens (human) | ARHGDIA | Signaling |
| H. sapiens (human) | ARID3A | Cell cycle |
| H. sapiens (human) | ARID4A | Chromatin organization |
| H. sapiens (human) | ARID4B | Chromatin organization |
| H. sapiens (human) | ATRIP | DNA replication and repair |
| H. sapiens (human) | AURKA | Cell cycle |
| H. sapiens (human) | AURKB | Cell cycle |
| H. sapiens (human) | AZGP1 | Protein transport |
| H. sapiens (human) | BARD1 | DNA replication and repair |
| H. sapiens (human) | BCCIP | DNA replication and repair |
| H. sapiens (human) | BRCA2 | DNA replication and repair |
| H. sapiens (human) | BRIP1 | DNA replication and repair |
| H. sapiens (human) | BUB3 | Cell cycle |
| H. sapiens (human) | BZW1 | RNA transcription and processing |
| H. sapiens (human) | C14ORF166 | RNA transcription and processing |
| H. sapiens (human) | FAAP100 | DNA replication and repair |
| H. sapiens (human) | FAAP24 | DNA replication and repair |
| H. sapiens (human) | C1QBP | Signaling |
| H. sapiens (human) | RTCB | RNA transcription and processing |
| H. sapiens (human) | TTI2 | Signaling |
| H. sapiens (human) | CACYBP | Signaling |
| H. sapiens (human) | CAND1 | --- not yet entered --- |
| H. sapiens (human) | CARM1 | Chromatin organization |
| H. sapiens (human) | CBFB | Cell differentiation |
| H. sapiens (human) | CBX1 | RNA transcription and processing |
| H. sapiens (human) | CBX5 | RNA transcription and processing |
| H. sapiens (human) | CCAR2 | Cell cycle |
| H. sapiens (human) | POLR1A | RNA transcription and processing |
| H. sapiens (human) | CCNA2 | Cell cycle |
| H. sapiens (human) | CCT2 | Protein synthesis and degradation |
| H. sapiens (human) | CCT4 | Protein synthesis and degradation |
| H. sapiens (human) | CCT5 | Protein synthesis and degradation |
| H. sapiens (human) | CCT7 | Protein synthesis and degradation |
| H. sapiens (human) | CDC25A | Cell cycle |
| H. sapiens (human) | CDC25B | Cell cycle |
| H. sapiens (human) | CDC73 | Cell cycle |
| H. sapiens (human) | CDCA8 | Cell cycle |
| H. sapiens (human) | CDK1 | Cell cycle |
| H. sapiens (human) | CEP164 | DNA replication and repair |
| H. sapiens (human) | CGGBP1 | RNA transcription and processing |
| H. sapiens (human) | CHAF1B | Chromatin organization |
| H. sapiens (human) | CHEK1 | Cell cycle |
| H. sapiens (human) | CHEK2 | Cell cycle |
| H. sapiens (human) | CHFR | Cell cycle |
| H. sapiens (human) | CHMP4B | Protein transport |
| H. sapiens (human) | CHTF18 | Cell cycle |
| H. sapiens (human) | CIRBP | Signaling |
| H. sapiens (human) | CLIC1 | Signaling |
| H. sapiens (human) | CLSPN | DNA replication and repair |
| H. sapiens (human) | CMPK1 | Metabolism |
| H. sapiens (human) | CPSF6 | RNA transcription and processing |
| H. sapiens (human) | CREBBP | Signaling |
| H. sapiens (human) | CSE1L | Protein transport |
| H. sapiens (human) | CSNK2A1 | Signaling |
| H. sapiens (human) | CSRP1 | Cell differentiation |
| H. sapiens (human) | CTBP1 | --- not yet entered --- |
| H. sapiens (human) | CTDP1 | Protein modification |
| H. sapiens (human) | DBF4B | Cell cycle |
| H. sapiens (human) | DCLRE1A | DNA replication and repair |
| H. sapiens (human) | DCLRE1B | Telomere biology |
| H. sapiens (human) | DCLRE1C | Telomere biology |
| H. sapiens (human) | DCTN2 | Cell cycle |
| H. sapiens (human) | DDB1 | DNA replication and repair |
| H. sapiens (human) | DDX1 | RNA transcription and processing |
| H. sapiens (human) | DDX39B | RNA transcription and processing |
| H. sapiens (human) | DDX5 | RNA transcription and processing |
| H. sapiens (human) | DHX15 | RNA transcription and processing |
| H. sapiens (human) | DKC1 | Telomere biology |
| H. sapiens (human) | DNAJA1 | Protein synthesis and degradation |
| H. sapiens (human) | DNAJA2 | Protein synthesis and degradation |
| H. sapiens (human) | DNAJB1 | Protein synthesis and degradation |
| H. sapiens (human) | DNAJC8 | RNA transcription and processing |
| H. sapiens (human) | DNAJC9 |  |
| H. sapiens (human) | DNMT3A | Chromatin organization |
| H. sapiens (human) | DNMT3B | Chromatin organization |
| H. sapiens (human) | DNTT | DNA replication and repair |
| H. sapiens (human) | DUT | DNA replication and repair |
| H. sapiens (human) | E2F1 | Cell cycle |
| H. sapiens (human) | E2F2 | Cell cycle |
| H. sapiens (human) | E2F3 | Cell cycle |
| H. sapiens (human) | E2F4 | Cell cycle |
| H. sapiens (human) | ECT2 | Protein transport |
| H. sapiens (human) | EDF1 | RNA transcription and processing |
| H. sapiens (human) | EFTUD2 | RNA transcription and processing |
| H. sapiens (human) | EID3 | DNA replication and repair |
| H. sapiens (human) | EIF4H | Protein synthesis and degradation |
| H. sapiens (human) | EIF5A | Protein transport |
| H. sapiens (human) | EIF5A2 | Protein transport |
| H. sapiens (human) | ELAVL1 | RNA transcription and processing |
| H. sapiens (human) | EMD | Cell differentiation |
| H. sapiens (human) | EME1 | DNA replication and repair |
| H. sapiens (human) | ERCC1 | DNA replication and repair |
| H. sapiens (human) | ERCC2 | DNA replication and repair |
| H. sapiens (human) | ERCC3 | DNA replication and repair |
| H. sapiens (human) | ERCC5 | DNA replication and repair |
| H. sapiens (human) | ESRRA | Signaling |
| H. sapiens (human) | ETAA1 | DNA replication and repair |
| H. sapiens (human) | ETFA | Metabolism |
| H. sapiens (human) | ETHE1 | Metabolism |
| H. sapiens (human) | EXO1 | DNA replication and repair |
| H. sapiens (human) | FAM175A | DNA replication and repair |
| H. sapiens (human) | FAM50A | Cell differentiation |
| H. sapiens (human) | FAM84A | DNA replication and repair |
| H. sapiens (human) | FAM84B | DNA replication and repair |
| H. sapiens (human) | FANCA | DNA replication and repair |
| H. sapiens (human) | FANCB | DNA replication and repair |
| H. sapiens (human) | FANCC | DNA replication and repair |
| H. sapiens (human) | FANCE | DNA replication and repair |
| H. sapiens (human) | FANCF | DNA replication and repair |
| H. sapiens (human) | FANCG | DNA replication and repair |
| H. sapiens (human) | FANCI | DNA replication and repair |
| H. sapiens (human) | FANCL | DNA replication and repair |
| H. sapiens (human) | FANCM | DNA replication and repair |
| H. sapiens (human) | FBL | RNA transcription and processing |
| H. sapiens (human) | FBXO18 | DNA replication and repair |
| H. sapiens (human) | FH | Metabolism |
| H. sapiens (human) | FKBP5 | Protein synthesis and degradation |
| H. sapiens (human) | FUBP1 | RNA transcription and processing |
| H. sapiens (human) | FUS | RNA transcription and processing |
| H. sapiens (human) | GAR1 | RNA transcription and processing |
| H. sapiens (human) | GATAD2B | RNA transcription and processing |
| H. sapiens (human) | GBE1 | Metabolism |
| H. sapiens (human) | GEN1 | DNA replication and repair |
| H. sapiens (human) | GET4 | Protein synthesis and degradation |
| H. sapiens (human) | GMNN | Cell cycle |
| H. sapiens (human) | GMPS | Metabolism |
| H. sapiens (human) | RACK1 | Cell cycle |
| H. sapiens (human) | GNL3 | Protein synthesis and degradation |
| H. sapiens (human) | GPKOW | RNA transcription and processing |
| H. sapiens (human) | GRWD1 | RNA transcription and processing |
| H. sapiens (human) | GTF3C3 | RNA transcription and processing |
| H. sapiens (human) | H2AFX | Chromatin organization |
| H. sapiens (human) | H2AFY | Chromatin organization |
| H. sapiens (human) | H2AFY2 | Chromatin organization |
| H. sapiens (human) | HAT1 | DNA replication and repair |
| H. sapiens (human) | HDAC1 | Chromatin organization |
| H. sapiens (human) | HDAC2 | Chromatin organization |
| H. sapiens (human) | HDAC3 | Chromatin organization |
| H. sapiens (human) | HDAC4 | Chromatin organization |
| H. sapiens (human) | HDGF | RNA transcription and processing |
| H. sapiens (human) | HELZ | RNA transcription and processing |
| H. sapiens (human) | HIC1 | RNA transcription and processing |
| H. sapiens (human) | HINT2 | Metabolism |
| H. sapiens (human) | HIST1H2AA | Chromatin organization |
| H. sapiens (human) | HIST1H2BA | Chromatin organization |
| H. sapiens (human) | HIST1H2BB | Chromatin organization |
| H. sapiens (human) | HIST1H4H | Chromatin organization |
| H. sapiens (human) | HIST3H3 | Chromatin organization |
| H. sapiens (human) | HLTF | RNA transcription and processing |
| H. sapiens (human) | HMGA1 | RNA transcription and processing |
| H. sapiens (human) | HMGB1 | Chromatin organization |
| H. sapiens (human) | HMGB1P10 | --- not yet entered --- |
| H. sapiens (human) | HMGB2 | Chromatin organization |
| H. sapiens (human) | HMGB3 | DNA replication and repair |
| H. sapiens (human) | HNRNPA1 | RNA transcription and processing |
| H. sapiens (human) | HP1BP3 | Chromatin organization |
| H. sapiens (human) | HSD17B10 | Metabolism |
| H. sapiens (human) | HUS1 | DNA replication and repair |
| H. sapiens (human) | IDH3A | Metabolism |
| H. sapiens (human) | INO80 | Chromatin organization |
| H. sapiens (human) | INO80B | Chromatin organization |
| H. sapiens (human) | INO80C | Chromatin organization |
| H. sapiens (human) | INO80E | Chromatin organization |
| H. sapiens (human) | ISG15 | Protein modification |
| H. sapiens (human) | ISG20 | RNA transcription and processing |
| H. sapiens (human) | ITGAX | Signaling |
| H. sapiens (human) | KAT2A | Chromatin organization |
| H. sapiens (human) | KAT2B | Chromatin organization |
| H. sapiens (human) | KDM1A | Chromatin organization |
| H. sapiens (human) | KDM4A | Chromatin organization |
| H. sapiens (human) | KDM4B | Chromatin organization |
| H. sapiens (human) | KDM4C | Chromatin organization |
| H. sapiens (human) | KHDRBS1 | Cell cycle |
| H. sapiens (human) | KHSRP | RNA transcription and processing |
| H. sapiens (human) | KIF4A | Cell cycle |
| H. sapiens (human) | KMT2A | Chromatin organization |
| H. sapiens (human) | KPNB1 | Protein transport |
| H. sapiens (human) | LASP1 | Cell membrane / wall |
| H. sapiens (human) | LEMD2 | Cellular structure |
| H. sapiens (human) | LGALS1 | Cell differentiation |
| H. sapiens (human) | LGALS7 | Cell death |
| H. sapiens (human) | LIG1 | DNA replication and repair |
| H. sapiens (human) | LIG3 | DNA replication and repair |
| H. sapiens (human) | LIG4 | DNA replication and repair |
| H. sapiens (human) | LMNB1 | Nuclear organization |
| H. sapiens (human) | LONP1 | Mitochondria |
| H. sapiens (human) | LRIF1 | RNA transcription and processing |
| H. sapiens (human) | LRRC41 | Protein modification |
| H. sapiens (human) | LRWD1 | Chromatin organization |
| H. sapiens (human) | MAT2B | Metabolism |
| H. sapiens (human) | MBD3 | Chromatin organization |
| H. sapiens (human) | MCM2 | Cell cycle |
| H. sapiens (human) | MCM3 | Cell cycle |
| H. sapiens (human) | MCM4 | Cell cycle |
| H. sapiens (human) | MCM5 | Cell cycle |
| H. sapiens (human) | MCM6 | Cell cycle |
| H. sapiens (human) | MCM7 | Cell cycle |
| H. sapiens (human) | MCPH1 | RNA transcription and processing |
| H. sapiens (human) | MCRS1 | Chromatin organization |
| H. sapiens (human) | MDC1 | DNA replication and repair |
| H. sapiens (human) | MKI67 | Cell cycle |
| H. sapiens (human) | MLH1 | Cell cycle |
| H. sapiens (human) | MORC4 | RNA transcription and processing |
| H. sapiens (human) | MRE11 | DNA replication and repair |
| H. sapiens (human) | MSH2 | DNA replication and repair |
| H. sapiens (human) | MSH3 | DNA replication and repair |
| H. sapiens (human) | MSH4 | DNA replication and repair |
| H. sapiens (human) | MSH5 | DNA replication and repair |
| H. sapiens (human) | MSH6 | DNA replication and repair |
| H. sapiens (human) | MTA1 | Chromatin organization |
| H. sapiens (human) | MTF2 | Chromatin organization |
| H. sapiens (human) | MUS81 | DNA replication and repair |
| H. sapiens (human) | MUTYH | DNA replication and repair |
| H. sapiens (human) | NABP1 | DNA replication and repair |
| H. sapiens (human) | NABP2 | Cell cycle |
| H. sapiens (human) | NACA | Protein synthesis and degradation |
| H. sapiens (human) | NAMPT | Metabolism |
| H. sapiens (human) | NANS | Metabolism |
| H. sapiens (human) | NAT10 | RNA transcription and processing |
| H. sapiens (human) | NBN | Cell cycle |
| H. sapiens (human) | NCAPG | Cell cycle |
| H. sapiens (human) | NDC80 | Cell cycle |
| H. sapiens (human) | NSMCE3 | DNA replication and repair |
| H. sapiens (human) | NELFB | RNA transcription and processing |
| H. sapiens (human) | NFRKB | DNA replication and repair |
| H. sapiens (human) | NHEJ1 | DNA replication and repair |
| H. sapiens (human) | NHP2 | RNA transcription and processing |
| H. sapiens (human) | NIPBL | Chromatin organization |
| H. sapiens (human) | NIPSNAP1 | Mitochondria |
| H. sapiens (human) | NOP10 | RNA transcription and processing |
| H. sapiens (human) | NOP9 | --- not yet entered --- |
| H. sapiens (human) | NOSIP | Metabolism |
| H. sapiens (human) | NR2C1 | RNA transcription and processing |
| H. sapiens (human) | NR2C2 | RNA transcription and processing |
| H. sapiens (human) | NR2F1 | RNA transcription and processing |
| H. sapiens (human) | NR2F6 | RNA transcription and processing |
| H. sapiens (human) | NRIP1 | RNA transcription and processing |
| H. sapiens (human) | NSMCE4A | DNA replication and repair |
| H. sapiens (human) | NSUN2 | Cell cycle |
| H. sapiens (human) | NUDC | Cell cycle |
| H. sapiens (human) | NUDT21 | RNA transcription and processing |
| H. sapiens (human) | NUMA1 | Cell cycle |
| H. sapiens (human) | STN1 | Telomere biology |
| H. sapiens (human) | OLA1 | Metabolism |
| H. sapiens (human) | ORC1 | Cell cycle |
| H. sapiens (human) | ORC2 | Cell cycle |
| H. sapiens (human) | ORC4 | Cell cycle |
| H. sapiens (human) | ORC5 | Cell cycle |
| H. sapiens (human) | ORC6 | Cell cycle |
| H. sapiens (human) | OTUB1 | DNA replication and repair |
| H. sapiens (human) | PA2G4 | RNA transcription and processing |
| H. sapiens (human) | PABPC1 | RNA transcription and processing |
| H. sapiens (human) | PALB2 | DNA replication and repair |
| H. sapiens (human) | PARK7 | RNA transcription and processing |
| H. sapiens (human) | PARP1 | Protein modification |
| H. sapiens (human) | PARP4 | Protein modification |
| H. sapiens (human) | PAXIP1 | Chromatin organization |
| H. sapiens (human) | PCNP | Cell cycle |
| H. sapiens (human) | PDCD4 | Cell death |
| H. sapiens (human) | PDCD6 | Cell death |
| H. sapiens (human) | PEBP1 | Signaling |
| H. sapiens (human) | PES1 | RNA transcription and processing |
| H. sapiens (human) | PHF1 | Chromatin organization |
| H. sapiens (human) | PHF20L1 | RNA transcription and processing |
| H. sapiens (human) | PIAS1 | Protein modification |
| H. sapiens (human) | PIAS3 | Protein modification |
| H. sapiens (human) | PIR | RNA transcription and processing |
| H. sapiens (human) | PKMYT1 | Cell cycle |
| H. sapiens (human) | PLK1 | Cell cycle |
| H. sapiens (human) | PMS1 | DNA replication and repair |
| H. sapiens (human) | PMS2 | DNA replication and repair |
| H. sapiens (human) | PNKP | DNA replication and repair |
| H. sapiens (human) | POLA1 | DNA replication and repair |
| H. sapiens (human) | POLA2 | DNA replication and repair |
| H. sapiens (human) | POLD1 | DNA replication and repair |
| H. sapiens (human) | POLD2 | DNA replication and repair |
| H. sapiens (human) | POLD3 | DNA replication and repair |
| H. sapiens (human) | POLD4 | DNA replication and repair |
| H. sapiens (human) | POLE | DNA replication and repair |
| H. sapiens (human) | POLE2 | DNA replication and repair |
| H. sapiens (human) | POLE3 | DNA replication and repair |
| H. sapiens (human) | POLE4 | DNA replication and repair |
| H. sapiens (human) | POLH | DNA replication and repair |
| H. sapiens (human) | POLL | DNA replication and repair |
| H. sapiens (human) | POLM | DNA replication and repair |
| H. sapiens (human) | POLR1B | RNA transcription and processing |
| H. sapiens (human) | POLR1C | RNA transcription and processing |
| H. sapiens (human) | POLR1E | RNA transcription and processing |
| H. sapiens (human) | POLR2E | RNA transcription and processing |
| H. sapiens (human) | PPME1 | Protein modification |
| H. sapiens (human) | PPP1CA | Chromatin organization |
| H. sapiens (human) | PPP1CB | Chromatin organization |
| H. sapiens (human) | PPP1CC | Chromatin organization |
| H. sapiens (human) | PPP1R10 | Chromatin organization |
| H. sapiens (human) | PRIM1 | DNA replication and repair |
| H. sapiens (human) | PRIM2 | DNA replication and repair |
| H. sapiens (human) | PRKDC | DNA replication and repair |
| H. sapiens (human) | PRMT1 | Chromatin organization |
| H. sapiens (human) | PRMT2 | Chromatin organization |
| H. sapiens (human) | PRMT3 | Chromatin organization |
| H. sapiens (human) | PRMT5 | --- not yet entered --- |
| H. sapiens (human) | PRMT6 | Chromatin organization |
| H. sapiens (human) | PRPF19 | DNA replication and repair |
| H. sapiens (human) | PRPF31 | RNA transcription and processing |
| H. sapiens (human) | PRPF4B | RNA transcription and processing |
| H. sapiens (human) | PSMA7 | Protein synthesis and degradation |
| H. sapiens (human) | PSMA8 | Protein synthesis and degradation |
| H. sapiens (human) | PSMB6 | Protein synthesis and degradation |
| H. sapiens (human) | PSMD10 | Protein synthesis and degradation |
| H. sapiens (human) | PSMD5 | Protein synthesis and degradation |
| H. sapiens (human) | PSME3 | Protein synthesis and degradation |
| H. sapiens (human) | PTMS | DNA replication and repair |
| H. sapiens (human) | RAB10 | Protein transport |
| H. sapiens (human) | RAB1B | Protein transport |
| H. sapiens (human) | RAB35 | Protein transport |
| H. sapiens (human) | RAB5C | Protein transport |
| H. sapiens (human) | RAB6A | Protein transport |
| H. sapiens (human) | RAD1 | DNA replication and repair |
| H. sapiens (human) | RAD17 | DNA replication and repair |
| H. sapiens (human) | RAD18 | DNA replication and repair |
| H. sapiens (human) | RAD21 | DNA replication and repair |
| H. sapiens (human) | RAD23B | DNA replication and repair |
| H. sapiens (human) | RAD50 | DNA replication and repair |
| H. sapiens (human) | RAD51 | DNA replication and repair |
| H. sapiens (human) | RAD51AP1 | DNA replication and repair |
| H. sapiens (human) | RAD51B | DNA replication and repair |
| H. sapiens (human) | RAD51C | DNA replication and repair |
| H. sapiens (human) | RAD51D | DNA replication and repair |
| H. sapiens (human) | RAD52 | DNA replication and repair |
| H. sapiens (human) | RAD54B | DNA replication and repair |
| H. sapiens (human) | RAD54L | DNA replication and repair |
| H. sapiens (human) | RAD9A | DNA replication and repair |
| H. sapiens (human) | RANP1 | --- not yet entered --- |
| H. sapiens (human) | RB1 | Cell cycle |
| H. sapiens (human) | RBBP7 | Chromatin organization |
| H. sapiens (human) | RBBP8 | Cell cycle |
| H. sapiens (human) | RBL1 | Cell cycle |
| H. sapiens (human) | RBL2 | Cell cycle |
| H. sapiens (human) | RBM14 | DNA replication and repair |
| H. sapiens (human) | RBM8A | RNA transcription and processing |
| H. sapiens (human) | RCC1 | Cell cycle |
| H. sapiens (human) | RCC2 | Cell cycle |
| H. sapiens (human) | RCOR1 | Cell differentiation |
| H. sapiens (human) | RECQL | DNA replication and repair |
| H. sapiens (human) | RECQL4 | DNA replication and repair |
| H. sapiens (human) | RECQL5 | DNA replication and repair |
| H. sapiens (human) | REV1 | DNA replication and repair |
| H. sapiens (human) | RFC1 | DNA replication and repair |
| H. sapiens (human) | RFC2 | DNA replication and repair |
| H. sapiens (human) | RFC3 | DNA replication and repair |
| H. sapiens (human) | RFC4 | DNA replication and repair |
| H. sapiens (human) | RFC5 | DNA replication and repair |
| H. sapiens (human) | RIF1 | DNA replication and repair |
| H. sapiens (human) | RMI1 | DNA replication and repair |
| H. sapiens (human) | RMI2 | DNA replication and repair |
| H. sapiens (human) | RNF8 | DNA replication and repair |
| H. sapiens (human) | RPA3 | DNA replication and repair |
| H. sapiens (human) | RPAIN | DNA replication and repair |
| H. sapiens (human) | RPRD1B | Cell cycle |
| H. sapiens (human) | RUVBL1 | Chromatin organization |
| H. sapiens (human) | RUVBL2 | DNA replication and repair |
| H. sapiens (human) | SAE1 | Protein modification |
| H. sapiens (human) | SARNP | RNA transcription and processing |
| H. sapiens (human) | 2-Sep | Cell cycle |
| H. sapiens (human) | SERPINB12 | Cell differentiation |
| H. sapiens (human) | SET | Chromatin organization |
| H. sapiens (human) | SFR1 | DNA replication and repair |
| H. sapiens (human) | SGO1 | Chromatin organization |
| H. sapiens (human) | SEM1 | DNA replication and repair |
| H. sapiens (human) | SHMT1 | Metabolism |
| H. sapiens (human) | SIMC1 | Protein modification |
| H. sapiens (human) | SIRT1 | Chromatin organization |
| H. sapiens (human) | SIRT6 | Chromatin organization |
| H. sapiens (human) | SKP1 | Cell cycle |
| H. sapiens (human) | SLX4 | DNA replication and repair |
| H. sapiens (human) | SLX4IP | --- not yet entered --- |
| H. sapiens (human) | SMARCA1 | Chromatin organization |
| H. sapiens (human) | SMARCA5 | Chromatin organization |
| H. sapiens (human) | SMARCC2 | Chromatin organization |
| H. sapiens (human) | SMC1A | Cell cycle |
| H. sapiens (human) | SMC3 | Cell cycle |
| H. sapiens (human) | SMC5 | DNA replication and repair |
| H. sapiens (human) | SMC6 | DNA replication and repair |
| H. sapiens (human) | SMCHD1 | Chromatin organization |
| H. sapiens (human) | SMG1 | RNA transcription and processing |
| H. sapiens (human) | SMG5 | RNA transcription and processing |
| H. sapiens (human) | SMG6 | RNA transcription and processing |
| H. sapiens (human) | SMG7 | RNA transcription and processing |
| H. sapiens (human) | SND1 | RNA transcription and processing |
| H. sapiens (human) | SNRPA1 | RNA transcription and processing |
| H. sapiens (human) | SNW1 | RNA transcription and processing |
| H. sapiens (human) | SOD2 | Mitochondria |
| H. sapiens (human) | SP110 | RNA transcription and processing |
| H. sapiens (human) | SRP14 | Cell membrane / wall |
| H. sapiens (human) | SRSF9 | RNA transcription and processing |
| H. sapiens (human) | SSB | RNA transcription and processing |
| H. sapiens (human) | SSRP1 | DNA replication and repair |
| H. sapiens (human) | STAG1 | Cell cycle |
| H. sapiens (human) | STAG2 | Cell cycle |
| H. sapiens (human) | STIP1 | Protein synthesis and degradation |
| H. sapiens (human) | STOML2 | Mitochondria |
| H. sapiens (human) | SUMO3 | Protein modification |
| H. sapiens (human) | SUPT16H | DNA replication and repair |
| H. sapiens (human) | SUV39H1 | Chromatin organization |
| H. sapiens (human) | SUV39H2 | Chromatin organization |
| H. sapiens (human) | KMT5B | Chromatin organization |
| H. sapiens (human) | KMT5C | Chromatin organization |
| H. sapiens (human) | SWI5 | DNA replication and repair |
| H. sapiens (human) | TAF15 | RNA transcription and processing |
| H. sapiens (human) | TAGLN2 | Cell differentiation |
| H. sapiens (human) | TARDBP | RNA transcription and processing |
| H. sapiens (human) | TCEA1 | RNA transcription and processing |
| H. sapiens (human) | TCOF1 | RNA transcription and processing |
| H. sapiens (human) | TDP1 | DNA replication and repair |
| H. sapiens (human) | TDRD1 | Cell differentiation |
| H. sapiens (human) | TDRD10 | Cell differentiation |
| H. sapiens (human) | TDRD12 | Cell differentiation |
| H. sapiens (human) | TDRD3 | Cell differentiation |
| H. sapiens (human) | TDRD5 | Cell differentiation |
| H. sapiens (human) | TDRD6 | Cell differentiation |
| H. sapiens (human) | TDRD7 | Cell differentiation |
| H. sapiens (human) | TDRD9 | Cell differentiation |
| H. sapiens (human) | TDRKH | Cell differentiation |
| H. sapiens (human) | TELO2 | Signaling |
| H. sapiens (human) | TERC | Telomere biology |
| H. sapiens (human) | TFPT | Cell death |
| H. sapiens (human) | TFRC | Cell membrane / wall |
| H. sapiens (human) | TMED10 | --- not yet entered --- |
| H. sapiens (human) | TMEM109 | Cell membrane / wall |
| H. sapiens (human) | TMEM201 | Cell membrane / wall |
| H. sapiens (human) | TNKS | Protein modification |
| H. sapiens (human) | TNKS2 | Protein modification |
| H. sapiens (human) | TOP1 | DNA replication and repair |
| H. sapiens (human) | TOP2A | DNA replication and repair |
| H. sapiens (human) | TOP2B | DNA replication and repair |
| H. sapiens (human) | TOP3B | DNA replication and repair |
| H. sapiens (human) | TOPBP1 | DNA replication and repair |
| H. sapiens (human) | TP53BP1 | DNA replication and repair |
| H. sapiens (human) | TPR | RNA transcription and processing |
| H. sapiens (human) | TREX1 | DNA replication and repair |
| H. sapiens (human) | TREX2 | DNA replication and repair |
| H. sapiens (human) | TRIM28 | Chromatin organization |
| H. sapiens (human) | TRIP13 | RNA transcription and processing |
| H. sapiens (human) | TTI1 | RNA transcription and processing |
| H. sapiens (human) | UAP1 | Metabolism |
| H. sapiens (human) | UBA1 | Protein synthesis and degradation |
| H. sapiens (human) | UBR5 | Chromatin organization |
| H. sapiens (human) | UBTF | RNA transcription and processing |
| H. sapiens (human) | UBXN1 | Protein synthesis and degradation |
| H. sapiens (human) | UCHL1 | Protein synthesis and degradation |
| H. sapiens (human) | UIMC1 | DNA replication and repair |
| H. sapiens (human) | UPF1 | RNA transcription and processing |
| H. sapiens (human) | USP7 | Protein synthesis and degradation |
| H. sapiens (human) | VCP | Protein transport |
| H. sapiens (human) | WDR61 | Chromatin organization |
| H. sapiens (human) | WDR82 | Chromatin organization |
| H. sapiens (human) | WEE1 | Cell cycle |
| H. sapiens (human) | WRN | DNA replication and repair |
| H. sapiens (human) | WRNIP1 | DNA replication and repair |
| H. sapiens (human) | XPO1 | RNA transcription and processing |
| H. sapiens (human) | XRCC1 | DNA replication and repair |
| H. sapiens (human) | XRCC2 | DNA replication and repair |
| H. sapiens (human) | XRCC3 | DNA replication and repair |
| H. sapiens (human) | XRCC4 | DNA replication and repair |
| H. sapiens (human) | XRCC5 | DNA replication and repair |
| H. sapiens (human) | YBX3 | Cell differentiation |
| H. sapiens (human) | YWHAB | Cell membrane / wall |
| H. sapiens (human) | YWHAE | Signaling |
| H. sapiens (human) | YWHAG | Cell cycle |
| H. sapiens (human) | YWHAQ | Cell membrane / wall |
| H. sapiens (human) | YWHAZ | Signaling |
| H. sapiens (human) | ZBTB44 | RNA transcription and processing |
| H. sapiens (human) | ZCCHC7 | RNA transcription and processing |
| H. sapiens (human) | ZMYM2 | RNA transcription and processing |
| H. sapiens (human) | ZNF827 | --- unknown --- |
| H. sapiens (human) | ZSWIM7 | DNA replication and repair |
| H. sapiens (human) | AR | RNA transcription and processing |
| H. sapiens (human) | BHLHE40 | RNA transcription and processing |
| H. sapiens (human) | BRCA1 | DNA replication and repair |
| H. sapiens (human) | CEBPB | RNA transcription and processing |
| H. sapiens (human) | CTCF | RNA transcription and processing |
| H. sapiens (human) | CTCFL | RNA transcription and processing |
| H. sapiens (human) | E2F5 | RNA transcription and processing |
| H. sapiens (human) | E2F6 | RNA transcription and processing |
| H. sapiens (human) | EGR1 | RNA transcription and processing |
| H. sapiens (human) | EPAS1 | RNA transcription and processing |
| H. sapiens (human) | ESR1 | RNA transcription and processing |
| H. sapiens (human) | ESR2 | RNA transcription and processing |
| H. sapiens (human) | ETS1 | RNA transcription and processing |
| H. sapiens (human) | ETS2 | RNA transcription and processing |
| H. sapiens (human) | ETV1 | RNA transcription and processing |
| H. sapiens (human) | EWSR1 | RNA transcription and processing |
| H. sapiens (human) | FOS | RNA transcription and processing |
| H. sapiens (human) | GLI1 | RNA transcription and processing |
| H. sapiens (human) | GLI2 | RNA transcription and processing |
| H. sapiens (human) | HIF1A | RNA transcription and processing |
| H. sapiens (human) | HOXA7 | RNA transcription and processing |
| H. sapiens (human) | IKZF1 | RNA transcription and processing |
| H. sapiens (human) | IRF1 | RNA transcription and processing |
| H. sapiens (human) | JUN | RNA transcription and processing |
| H. sapiens (human) | JUND | RNA transcription and processing |
| H. sapiens (human) | MAD1L1 | Cell cycle |
| H. sapiens (human) | MAZ | RNA transcription and processing |
| H. sapiens (human) | MBD2 | RNA transcription and processing |
| H. sapiens (human) | MEN1 | RNA transcription and processing |
| H. sapiens (human) | MITF | RNA transcription and processing |
| H. sapiens (human) | MXD1 | RNA transcription and processing |
| H. sapiens (human) | MYB | RNA transcription and processing |
| H. sapiens (human) | MYC | Cell cycle |
| H. sapiens (human) | MYCN | RNA transcription and processing |
| H. sapiens (human) | MZF1 | RNA transcription and processing |
| H. sapiens (human) | NFAT5 | RNA transcription and processing |
| H. sapiens (human) | NFATC2 | RNA transcription and processing |
| H. sapiens (human) | NFKB1 | Signaling |
| H. sapiens (human) | RELA | RNA transcription and processing |
| H. sapiens (human) | NFX1 | RNA transcription and processing |
| H. sapiens (human) | NR2F2 | RNA transcription and processing |
| H. sapiens (human) | PAX5 | RNA transcription and processing |
| H. sapiens (human) | PAX8 | RNA transcription and processing |
| H. sapiens (human) | PITX1 | RNA transcription and processing |
| H. sapiens (human) | RUNX2 | RNA transcription and processing |
| H. sapiens (human) | SMAD3 | RNA transcription and processing |
| H. sapiens (human) | SP1 | RNA transcription and processing |
| H. sapiens (human) | SP3 | RNA transcription and processing |
| H. sapiens (human) | STAT3 | RNA transcription and processing |
| H. sapiens (human) | STAT5A | RNA transcription and processing |
| H. sapiens (human) | TCF7 | RNA transcription and processing |
| H. sapiens (human) | TCF7L2 | RNA transcription and processing |
| H. sapiens (human) | TFAP2A | RNA transcription and processing |
| H. sapiens (human) | TFAP2B | RNA transcription and processing |
| H. sapiens (human) | TFAP4 | RNA transcription and processing |
| H. sapiens (human) | TP53 | RNA transcription and processing |
| H. sapiens (human) | TP73 | RNA transcription and processing |
| H. sapiens (human) | USF1 | RNA transcription and processing |
| H. sapiens (human) | WT1 | RNA transcription and processing |
| H. sapiens (human) | ZBTB48 | Telomere biology |
| H. sapiens (human) | BAZ2A | Chromatin organization |
| H. sapiens (human) | KLF4 | Telomere biology |
| H. sapiens (human) | CTNNB1 | Signaling |
| H. sapiens (human) | TEN1 | Telomere biology |
| H. sapiens (human) | ORC3 | Cell cycle |
| H. sapiens (human) | ETV4 | RNA transcription and processing |
| H. sapiens (human) | FLI1 | RNA transcription and processing |
| H. sapiens (human) | ACD | Telomere biology |
| H. sapiens (human) | ACYP2 | Metabolism |
| H. sapiens (human) | TSPYL6 | --- unknown --- |
| H. sapiens (human) | NAF1 | RNA transcription and processing |
| H. sapiens (human) | ZNF208 | RNA transcription and processing |
| H. sapiens (human) | MPHOSPH6 | RNA transcription and processing |
| H. sapiens (human) | DNA2 | DNA replication and repair |
| H. sapiens (human) | HRG | Signaling |
| H. sapiens (human) | IDH1 | Metabolism |
| H. sapiens (human) | RINT1 | Signaling |
| H. sapiens (human) | TNKS1BP1 | DNA replication and repair |
| H. sapiens (human) | ZSCAN4 | RNA transcription and processing |
| H. sapiens (human) | TP53RK | --- not yet entered --- |
| H. sapiens (human) | TPRKB | Metabolism |
| H. sapiens (human) | MYEF2 | --- not yet entered --- |
| H. sapiens (human) | HNRNPM | --- not yet entered --- |
| H. sapiens (human) | OSGEP | --- not yet entered --- |
| H. sapiens (human) | PIF1 | DNA replication and repair |
| H. sapiens (human) | KMT2C | Chromatin organization |
| H. sapiens (human) | KMT2B | Chromatin organization |
| H. sapiens (human) | SERBP1 | RNA transcription and processing |
| H. sapiens (human) | HABP4 | Chromatin organization |
| H. sapiens (human) | YRDC | Mitochondria |
| H. sapiens (human) | RBBP5 | Chromatin organization |
| H. sapiens (human) | ATAD5 | --- not yet entered --- |
| H. sapiens (human) | RRAGA | --- not yet entered --- |
| H. sapiens (human) | RRAGB | --- not yet entered --- |
| H. sapiens (human) | TNIP3 | --- not yet entered --- |
| H. sapiens (human) | TNIP2 | --- not yet entered --- |
| H. sapiens (human) | TNIP1 | DNA replication and repair |
| H. sapiens (human) | MMS19 | DNA replication and repair |
| H. sapiens (human) | THOC7 | RNA transcription and processing |
| H. sapiens (human) | PIAS4 | --- not yet entered --- |
| H. sapiens (human) | RPL13 | --- not yet entered --- |
| H. sapiens (human) | PBRM1 | Chromatin organization |
| H. sapiens (human) | SAP30 | Chromatin organization |
| H. sapiens (human) | SAP30L | Chromatin organization |
| H. sapiens (human) | CHMP4BP1 | --- not yet entered --- |
| H. sapiens (human) | CHMP4A | Protein transport |
| H. sapiens (human) | MED18 | RNA transcription and processing |
| H. sapiens (human) | CCNL1 | --- not yet entered --- |
| H. sapiens (human) | CCNC | RNA transcription and processing |
| H. sapiens (human) | CCNL2 | RNA transcription and processing |
| H. sapiens (human) | CCNT1 | RNA transcription and processing |
| H. sapiens (human) | CCNT2 | RNA transcription and processing |
| H. sapiens (human) | FAM58A | --- not yet entered --- |
| H. sapiens (human) | UBE2A | DNA replication and repair |
| H. sapiens (human) | UBE2B | DNA replication and repair |
| H. sapiens (human) | OSGEPL1 | Mitochondria |
| H. sapiens (human) | KMT2D | Chromatin organization |
| H. sapiens (human) | SETD1A | Chromatin organization |
| H. sapiens (human) | SETD1B | Chromatin organization |
| H. sapiens (human) | EZH1 | Chromatin organization |
| H. sapiens (human) | EZH2 | Chromatin organization |
| H. sapiens (human) | SIRT4 | Mitochondria |
| H. sapiens (human) | SIRT5 | Mitochondria |
| H. sapiens (human) | CHMP4C | Protein transport |
| H. sapiens (human) | CHMP7 | Cell cycle |
| H. sapiens (human) | NCBP2L | --- not yet entered --- |
| H. sapiens (human) | NCBP2 | RNA transcription and processing |
| H. sapiens (human) | NAA11 | --- not yet entered --- |
| H. sapiens (human) | NAA10 | --- not yet entered --- |
| H. sapiens (human) | HIST1H4B | DNA replication and repair |
| H. sapiens (human) | HIST4H4 | DNA replication and repair |
| H. sapiens (human) | POLR2B | RNA transcription and processing |
| H. sapiens (human) | THOC5 | RNA transcription and processing |
| H. sapiens (human) | TOX4 | Cell cycle |
| H. sapiens (human) | THOC1 | RNA transcription and processing |
| H. sapiens (human) | THOC2 | RNA transcription and processing |
| H. sapiens (human) | THOC6 | --- not yet entered --- |
| H. sapiens (human) | THOC3 | RNA transcription and processing |
| H. sapiens (human) | CTC1 | Telomere biology |
| H. sapiens (human) | ACAT2 | --- not yet entered --- |
| H. sapiens (human) | ACTB | --- not yet entered --- |
| H. sapiens (human) | ACY1 | --- not yet entered --- |
| H. sapiens (human) | ADPRH | --- not yet entered --- |
| H. sapiens (human) | AHCY | --- not yet entered --- |
| H. sapiens (human) | AKT1 | --- not yet entered --- |
| H. sapiens (human) | ALDH3A1 | --- not yet entered --- |
| H. sapiens (human) | ALDOA | --- not yet entered --- |
| H. sapiens (human) | AMPD2 | --- not yet entered --- |
| H. sapiens (human) | AMPH | Cell membrane / wall |
| H. sapiens (human) | SLC25A6 | Mitochondria |
| H. sapiens (human) | ANXA2 | Cell membrane / wall |
| H. sapiens (human) | ANXA4 | Cell membrane / wall |
| H. sapiens (human) | ANXA5 | --- not yet entered --- |
| H. sapiens (human) | ARRB1 | Chromatin organization |
| H. sapiens (human) | ZFP36L1 | RNA transcription and processing |
| H. sapiens (human) | CALD1 | Signaling |
| H. sapiens (human) | CALML3 | Protein synthesis and degradation |
| H. sapiens (human) | CARS | --- not yet entered --- |
| H. sapiens (human) | CKB | --- not yet entered --- |
| H. sapiens (human) | CLK3 | RNA transcription and processing |
| H. sapiens (human) | CRK | Cell membrane / wall |
| H. sapiens (human) | CRYBB1 | --- not yet entered --- |
| H. sapiens (human) | CRYGS | --- not yet entered --- |
| H. sapiens (human) | CRYM | Metabolism |
| H. sapiens (human) | DBN1 | --- not yet entered --- |
| H. sapiens (human) | DCX | --- not yet entered --- |
| H. sapiens (human) | DPYSL3 | --- not yet entered --- |
| H. sapiens (human) | EEF1D | --- not yet entered --- |
| H. sapiens (human) | EIF4B | --- not yet entered --- |
| H. sapiens (human) | CTTN | Cell membrane / wall |
| H. sapiens (human) | ENO2 | --- not yet entered --- |
| H. sapiens (human) | ENSA | --- not yet entered --- |
| H. sapiens (human) | EPB41L1 | Cell membrane / wall |
| H. sapiens (human) | EPHX2 | --- not yet entered --- |
| H. sapiens (human) | FABP2 | --- not yet entered --- |
| H. sapiens (human) | FES | Cell differentiation |
| H. sapiens (human) | NR5A1 | --- not yet entered --- |
| H. sapiens (human) | GAGE5 | --- not yet entered --- |
| H. sapiens (human) | GAMT | --- not yet entered --- |
| H. sapiens (human) | GAPDH | DNA replication and repair |
| H. sapiens (human) | GSS | --- not yet entered --- |
| H. sapiens (human) | HIST1H1A | Chromatin organization |
| H. sapiens (human) | HAGH | Mitochondria |
| H. sapiens (human) | HCLS1 | --- not yet entered --- |
| H. sapiens (human) | HGD | --- not yet entered --- |
| H. sapiens (human) | HLCS | --- not yet entered --- |
| H. sapiens (human) | HMGN1 | Chromatin organization |
| H. sapiens (human) | HMGN2 | Chromatin organization |
| H. sapiens (human) | HMOX1 | --- not yet entered --- |
| H. sapiens (human) | HNMT | --- not yet entered --- |
| H. sapiens (human) | HOXA3 | --- not yet entered --- |
| H. sapiens (human) | HSPA1A | Cell membrane / wall |
| H. sapiens (human) | HSPA6 | Cell membrane / wall |
| H. sapiens (human) | IL1RN | --- not yet entered --- |
| H. sapiens (human) | IVL | --- not yet entered --- |
| H. sapiens (human) | RPSA | Cell membrane / wall |
| H. sapiens (human) | LDHA | --- not yet entered --- |
| H. sapiens (human) | LDHB | --- not yet entered --- |
| H. sapiens (human) | LTA4H | --- not yet entered --- |
| H. sapiens (human) | MAGEA4 | --- not yet entered --- |
| H. sapiens (human) | MDH1 | --- not yet entered --- |
| H. sapiens (human) | MPG | --- not yet entered --- |
| H. sapiens (human) | MSN | Cell membrane / wall |
| H. sapiens (human) | MT1X | --- not yet entered --- |
| H. sapiens (human) | MT3 | --- not yet entered --- |
| H. sapiens (human) | MVK | Metabolism |
| H. sapiens (human) | NAP1L1 | Chromatin organization |
| H. sapiens (human) | NASP | Chromatin organization |
| H. sapiens (human) | NCL | Chromatin organization |
| H. sapiens (human) | NEFL | --- not yet entered --- |
| H. sapiens (human) | PRDX1 | --- not yet entered --- |
| H. sapiens (human) | PALM | Cell membrane / wall |
| H. sapiens (human) | PCP4 | --- not yet entered --- |
| H. sapiens (human) | PDE1B | --- not yet entered --- |
| H. sapiens (human) | PEPD | --- not yet entered --- |
| H. sapiens (human) | PFKP | --- not yet entered --- |
| H. sapiens (human) | PGM1 | --- not yet entered --- |
| H. sapiens (human) | PNMT | --- not yet entered --- |
| H. sapiens (human) | PPM1G | --- not yet entered --- |
| H. sapiens (human) | PPP1R2 | --- not yet entered --- |
| H. sapiens (human) | MAP2K3 | --- not yet entered --- |
| H. sapiens (human) | PTMA | --- not yet entered --- |
| H. sapiens (human) | PEX5 | --- not yet entered --- |
| H. sapiens (human) | MAP4K2 | --- not yet entered --- |
| H. sapiens (human) | RABIF | --- not yet entered --- |
| H. sapiens (human) | RBMY1A1 | --- not yet entered --- |
| H. sapiens (human) | RGS3 | --- not yet entered --- |
| H. sapiens (human) | RPL19 | --- not yet entered --- |
| H. sapiens (human) | S100P | --- not yet entered --- |
| H. sapiens (human) | MAPK12 | DNA replication and repair |
| H. sapiens (human) | SARS | --- not yet entered --- |
| H. sapiens (human) | SH3GL2 | Cell membrane / wall |
| H. sapiens (human) | SNCG | --- not yet entered --- |
| H. sapiens (human) | SOAT1 | Cell membrane / wall |
| H. sapiens (human) | SOX5 | --- not yet entered --- |
| H. sapiens (human) | SRM | --- not yet entered --- |
| H. sapiens (human) | SUPT5H | RNA transcription and processing |
| H. sapiens (human) | SYK | Cell membrane / wall |
| H. sapiens (human) | TAGLN | --- not yet entered --- |
| H. sapiens (human) | TALDO1 | --- not yet entered --- |
| H. sapiens (human) | TBCA | Protein synthesis and degradation |
| H. sapiens (human) | TBCD | Cell membrane / wall |
| H. sapiens (human) | TBL1X | --- not yet entered --- |
| H. sapiens (human) | TKT | --- not yet entered --- |
| H. sapiens (human) | TPI1 | --- not yet entered --- |
| H. sapiens (human) | TUBB2A | --- not yet entered --- |
| H. sapiens (human) | WARS | --- not yet entered --- |
| H. sapiens (human) | WFS1 | --- not yet entered --- |
| H. sapiens (human) | RNF113A | --- not yet entered --- |
| H. sapiens (human) | IFRD2 | --- not yet entered --- |
| H. sapiens (human) | HIST1H2AL | DNA replication and repair |
| H. sapiens (human) | FKBP6 | Cell differentiation |
| H. sapiens (human) | MADD | --- not yet entered --- |
| H. sapiens (human) | PEA15 | Cell membrane / wall |
| H. sapiens (human) | CPNE3 | --- not yet entered --- |
| H. sapiens (human) | NOL3 | Mitochondria |
| H. sapiens (human) | CLIC3 | Cell membrane / wall |
| H. sapiens (human) | DOK2 | --- not yet entered --- |
| H. sapiens (human) | MAP7 | Cell membrane / wall |
| H. sapiens (human) | TMSB4Y | --- not yet entered --- |
| H. sapiens (human) | TMSB10 | --- not yet entered --- |
| H. sapiens (human) | DDX21 | RNA transcription and processing |
| H. sapiens (human) | BCL7B | --- not yet entered --- |
| H. sapiens (human) | GPR52 | --- not yet entered --- |
| H. sapiens (human) | TRIP10 | Cell membrane / wall |
| H. sapiens (human) | HMGN3 | DNA replication and repair |
| H. sapiens (human) | DDX23 | --- not yet entered --- |
| H. sapiens (human) | XAGE2 | --- not yet entered --- |
| H. sapiens (human) | BAG3 | Protein synthesis and degradation |
| H. sapiens (human) | PRDX6 | --- not yet entered --- |
| H. sapiens (human) | DHX38 | --- not yet entered --- |
| H. sapiens (human) | KEAP1 | --- not yet entered --- |
| H. sapiens (human) | RNF10 | --- not yet entered --- |
| H. sapiens (human) | GFPT2 | --- not yet entered --- |
| H. sapiens (human) | DPP3 | --- not yet entered --- |
| H. sapiens (human) | TSSC4 | --- not yet entered --- |
| H. sapiens (human) | BCAP31 | Protein synthesis and degradation |
| H. sapiens (human) | GPA33 | --- not yet entered --- |
| H. sapiens (human) | STUB1 | DNA replication and repair |
| H. sapiens (human) | PAK4 | --- not yet entered --- |
| H. sapiens (human) | RTN3 | DNA replication and repair |
| H. sapiens (human) | AKR1A1 | --- not yet entered --- |
| H. sapiens (human) | TUBB3 | --- not yet entered --- |
| H. sapiens (human) | HMGN4 | --- not yet entered --- |
| H. sapiens (human) | PAICS | --- not yet entered --- |
| H. sapiens (human) | ARID3B | --- not yet entered --- |
| H. sapiens (human) | TRIM16 | --- not yet entered --- |
| H. sapiens (human) | GAS2L1 | --- not yet entered --- |
| H. sapiens (human) | RGS14 | --- not yet entered --- |
| H. sapiens (human) | RBBP9 | --- not yet entered --- |
| H. sapiens (human) | WDR4 | --- not yet entered --- |
| H. sapiens (human) | TOMM34 | Mitochondria |
| H. sapiens (human) | OGFR | --- not yet entered --- |
| H. sapiens (human) | WDR5 | Chromatin organization |
| H. sapiens (human) | PACSIN2 | Cell membrane / wall |
| H. sapiens (human) | AKR7A3 | --- not yet entered --- |
| H. sapiens (human) | NCDN | --- not yet entered --- |
| H. sapiens (human) | COTL1 | Protein synthesis and degradation |
| H. sapiens (human) | HAAO | --- not yet entered --- |
| H. sapiens (human) | SH3BP1 | Cell membrane / wall |
| H. sapiens (human) | AIPL1 | --- not yet entered --- |
| H. sapiens (human) | FKBP8 | Mitochondria |
| H. sapiens (human) | PGLS | --- not yet entered --- |
| H. sapiens (human) | SULT4A1 | --- not yet entered --- |
| H. sapiens (human) | CHMP2B | Protein transport |
| H. sapiens (human) | RPAP1 | Chromatin organization |
| H. sapiens (human) | CCDC9 | --- not yet entered --- |
| H. sapiens (human) | OR2H1 | --- not yet entered --- |
| H. sapiens (human) | GNMT | --- not yet entered --- |
| H. sapiens (human) | SULT1B1 | --- not yet entered --- |
| H. sapiens (human) | C19orf53 | --- not yet entered --- |
| H. sapiens (human) | DBNL | --- not yet entered --- |
| H. sapiens (human) | PHPT1 | --- not yet entered --- |
| H. sapiens (human) | PACSIN3 | Cell membrane / wall |
| H. sapiens (human) | PACSIN1 | Cell membrane / wall |
| H. sapiens (human) | TNPO2 | --- not yet entered --- |
| H. sapiens (human) | HDGFRP3 | --- not yet entered --- |
| H. sapiens (human) | SBDS | --- not yet entered --- |
| H. sapiens (human) | PIPOX | --- not yet entered --- |
| H. sapiens (human) | BIN2 | --- not yet entered --- |
| H. sapiens (human) | ISYNA1 | --- not yet entered --- |
| H. sapiens (human) | SCLY | --- not yet entered --- |
| H. sapiens (human) | C9orf78 | --- not yet entered --- |
| H. sapiens (human) | PRMT7 | Chromatin organization |
| H. sapiens (human) | KLHDC4 | --- not yet entered --- |
| H. sapiens (human) | EPS8L1 | Cell membrane / wall |
| H. sapiens (human) | RPP25 | --- not yet entered --- |
| H. sapiens (human) | BANP | --- not yet entered --- |
| H. sapiens (human) | PGM2 | --- not yet entered --- |
| H. sapiens (human) | UFSP2 | --- not yet entered --- |
| H. sapiens (human) | TRMT1 | --- not yet entered --- |
| H. sapiens (human) | LYAR | --- not yet entered --- |
| H. sapiens (human) | ENAH | --- not yet entered --- |
| H. sapiens (human) | LANCL2 | Cell membrane / wall |
| H. sapiens (human) | RGMA | --- not yet entered --- |
| H. sapiens (human) | AKR1B10 | --- not yet entered --- |
| H. sapiens (human) | ANKMY2 | --- not yet entered --- |
| H. sapiens (human) | DDX24 | --- not yet entered --- |
| H. sapiens (human) | CAMK1D | --- not yet entered --- |
| H. sapiens (human) | RTN4 | Mitochondria |
| H. sapiens (human) | CORO1B | --- not yet entered --- |
| H. sapiens (human) | KIAA1191 | --- not yet entered --- |
| H. sapiens (human) | PRX | Cell membrane / wall |
| H. sapiens (human) | RBKS | --- not yet entered --- |
| H. sapiens (human) | PDLIM2 | --- not yet entered --- |
| H. sapiens (human) | C12orf43 | --- not yet entered --- |
| H. sapiens (human) | AHNAK | --- not yet entered --- |
| H. sapiens (human) | C1orf35 | --- not yet entered --- |
| H. sapiens (human) | RPAP3 | Protein synthesis and degradation |
| H. sapiens (human) | KCTD17 | --- not yet entered --- |
| H. sapiens (human) | CCM2 | --- not yet entered --- |
| H. sapiens (human) | FERMT3 | --- not yet entered --- |
| H. sapiens (human) | TBC1D10A | --- not yet entered --- |
| H. sapiens (human) | ZNF414 | --- not yet entered --- |
| H. sapiens (human) | AFAP1L2 | --- not yet entered --- |
| H. sapiens (human) | PTPN5 | --- not yet entered --- |
| H. sapiens (human) | SCRN2 | --- not yet entered --- |
| H. sapiens (human) | LRSAM1 | --- not yet entered --- |
| H. sapiens (human) | PAGE5 | --- not yet entered --- |
| H. sapiens (human) | SYAP1 | --- not yet entered --- |
| H. sapiens (human) | NXNL1 | --- not yet entered --- |
| H. sapiens (human) | CCDC43 | --- not yet entered --- |
| H. sapiens (human) | C17orf49 | Chromatin organization |
| H. sapiens (human) | JSRP1 | Cell membrane / wall |
| H. sapiens (human) | LRRC25 | --- not yet entered --- |
| H. sapiens (human) | NUDCD2 | Protein synthesis and degradation |
| H. sapiens (human) | APOBEC3F | DNA replication and repair |
| H. sapiens (human) | DHFRL1 | Mitochondria |
| H. sapiens (human) | TUBB | --- not yet entered --- |
| H. sapiens (human) | NAIF1 | --- not yet entered --- |
| H. sapiens (human) | PAGE2 | --- not yet entered --- |
| H. sapiens (human) | CPNE2 | Cell membrane / wall |
| H. sapiens (human) | PHYHD1 | --- not yet entered --- |
| H. sapiens (human) | NUDT14 | --- not yet entered --- |
| H. sapiens (human) | BOD1L1 | DNA replication and repair |
| H. sapiens (human) | CCDC137 | --- not yet entered --- |
| H. sapiens (human) | C1orf174 | --- not yet entered --- |
| H. sapiens (human) | ANXA8L1 | --- not yet entered --- |
| H. sapiens (human) | MICA | --- not yet entered --- |
| H. sapiens (human) | ASS1 | --- not yet entered --- |
| H. sapiens (human) | HNRNPK | RNA transcription and processing |
| H. sapiens (human) | HSP90AB1 | Chromatin organization |
| H. sapiens (human) | IDO1 | --- not yet entered --- |
| H. sapiens (human) | IPO5 | Chromatin organization |
| H. sapiens (human) | OCM2 | --- not yet entered --- |
| H. sapiens (human) | PKM | --- not yet entered --- |
| H. sapiens (human) | PRKCB | Mitochondria |
| H. sapiens (human) | SRSF6 | --- not yet entered --- |
| H. sapiens (human) | SULT1C2 | --- not yet entered --- |
| H. sapiens (human) | AIMP2 | --- not yet entered --- |
| H. sapiens (human) | EIF3G | --- not yet entered --- |
| H. sapiens (human) | VGLL4 | --- not yet entered --- |
| H. sapiens (human) | FAM131B | --- not yet entered --- |
| H. sapiens (human) | DDX39A | --- not yet entered --- |
| H. sapiens (human) | DNPH1 | --- not yet entered --- |
| H. sapiens (human) | HEXIM1 | RNA transcription and processing |
| H. sapiens (human) | ARPP21 | --- not yet entered --- |
| H. sapiens (human) | DDX19B | --- not yet entered --- |
| H. sapiens (human) | ACOT7 | --- not yet entered --- |
| H. sapiens (human) | TWF2 | --- not yet entered --- |
| H. sapiens (human) | TOR1AIP1 | --- not yet entered --- |
| H. sapiens (human) | WIPI2 | Cell membrane / wall |
| H. sapiens (human) | KIF1BP | --- not yet entered --- |
| H. sapiens (human) | BABAM1 | Chromatin organization |
| H. sapiens (human) | LGALSL | --- not yet entered --- |
| H. sapiens (human) | MRTO4 | --- not yet entered --- |
| H. sapiens (human) | THG1L | RNA transcription and processing |
| H. sapiens (human) | MAGOHB | --- not yet entered --- |
| H. sapiens (human) | APPL2 | --- not yet entered --- |
| H. sapiens (human) | PPP6R3 | --- not yet entered --- |
| H. sapiens (human) | CPPED1 | --- not yet entered --- |
| H. sapiens (human) | C19orf66 | DNA replication and repair |
| H. sapiens (human) | CEP85 | --- not yet entered --- |
| H. sapiens (human) | NOL12 | --- not yet entered --- |
| H. sapiens (human) | NUDT18 | --- not yet entered --- |
| H. sapiens (human) | PYM1 | RNA transcription and processing |
| H. sapiens (human) | HDGFRP2 | --- not yet entered --- |
| H. sapiens (human) | TXNDC17 | --- not yet entered --- |
| H. sapiens (human) | PHYKPL | --- not yet entered --- |
| H. sapiens (human) | C15orf57 | --- not yet entered --- |
| H. sapiens (human) | FAM122A | --- not yet entered --- |
| H. sapiens (human) | ZC3H18 | --- not yet entered --- |
| H. sapiens (human) | HEXIM2 | RNA transcription and processing |
| H. sapiens (human) | SIRPD | --- not yet entered --- |
| H. sapiens (human) | REM2 | --- not yet entered --- |
| H. sapiens (human) | CNST | Cell membrane / wall |
| H. sapiens (human) | PROSER2 | --- not yet entered --- |
| H. sapiens (human) | ZNF790 | --- not yet entered --- |
| H. sapiens (human) | PDGFRA |  |
| H. sapiens (human) | MORC3 | RNA transcription and processing |
| H. sapiens (human) | CDK2 | DNA replication and repair |
| H. sapiens (human) | RNF4 | --- not yet entered --- |
| H. sapiens (human) | SENP2 | --- not yet entered --- |
| H. sapiens (human) | ZNF451 | Chromatin organization |
| H. sapiens (human) | HNRNPA2B1 | RNA transcription and processing |
| H. sapiens (human) | RAP1A | Cell membrane / wall |
| H. sapiens (human) | SENP1 | --- not yet entered --- |
| H. sapiens (human) | SENP3 | --- not yet entered --- |
| H. sapiens (human) | SENP5 | --- not yet entered --- |
| H. sapiens (human) | SENP7 | --- not yet entered --- |
| H. sapiens (human) | CASP7 | --- not yet entered --- |
| H. sapiens (human) | PGD | --- not yet entered --- |
| H. sapiens (human) | MAP4 | --- not yet entered --- |
| H. sapiens (human) | FBP1 | --- not yet entered --- |
| H. sapiens (human) | PAFAH1B3 | --- not yet entered --- |
| H. sapiens (human) | EVL | --- not yet entered --- |
| H. sapiens (human) | CFL1 | Cell membrane / wall |
| H. sapiens (human) | ADA | --- not yet entered --- |
| H. sapiens (human) | TRIM15 | --- not yet entered --- |
| H. sapiens (human) | AK1 | --- not yet entered --- |
| H. sapiens (human) | TUBB4B | --- not yet entered --- |
| H. sapiens (human) | NCBP1 | --- not yet entered --- |
| H. sapiens (human) | CPSF4 | RNA transcription and processing |
| H. sapiens (human) | GRHL2 | DNA replication and repair |
| H. sapiens (human) | HNRNPD | RNA transcription and processing |
| H. sapiens (human) | HSP90AA1 | Chromatin organization |
| H. sapiens (human) | NOS3 | Signaling |
| H. sapiens (human) | RFPL3 | RNA transcription and processing |
| H. sapiens (human) | SCYL1 | RNA transcription and processing |
| H. sapiens (human) | ARID1A | Chromatin organization |
| H. sapiens (human) | DEK | Chromatin organization |
| H. sapiens (human) | KLF2 | RNA transcription and processing |
| H. sapiens (human) | SNAI1 | Chromatin organization |
| H. sapiens (human) | TAL1 | Cell differentiation |
| H. sapiens (human) | VDR | Chromatin organization |
| H. sapiens (human) | AVPR1B | --- not yet entered --- |
| H. sapiens (human) | CAMK2A | --- not yet entered --- |
| H. sapiens (human) | CASK | Cell membrane / wall |
| H. sapiens (human) | CDKL2 | --- not yet entered --- |
| H. sapiens (human) | DLG2 | --- not yet entered --- |
| H. sapiens (human) | DLG4 | --- not yet entered --- |
| H. sapiens (human) | DNAJC3 | Protein synthesis and degradation |
| H. sapiens (human) | EDN2 | --- not yet entered --- |
| H. sapiens (human) | EIF2AK4 | Protein modification |
| H. sapiens (human) | FGFR3 | --- not yet entered --- |
| H. sapiens (human) | GNE | --- not yet entered --- |
| H. sapiens (human) | KSR2 | --- not yet entered --- |
| H. sapiens (human) | LCP2 | --- not yet entered --- |
| H. sapiens (human) | MAP3K4 | Protein modification |
| H. sapiens (human) | MAPK1 | --- not yet entered --- |
| H. sapiens (human) | MAPK14 | Chromatin organization |
| H. sapiens (human) | NEK2 | Cell cycle |
| H. sapiens (human) | NEK6 | Chromatin organization |
| H. sapiens (human) | NEK7 | Telomere biology |
| H. sapiens (human) | NEK8 | --- not yet entered --- |
| H. sapiens (human) | RAC1 | --- not yet entered --- |
| H. sapiens (human) | RPS6KA5 | Chromatin organization |
| H. sapiens (human) | RYK | --- not yet entered --- |
| H. sapiens (human) | AKAP8 | DNA replication and repair |
| H. sapiens (human) | ANGPT4 | --- not yet entered --- |
| H. sapiens (human) | BDKRB2 | --- not yet entered --- |
| H. sapiens (human) | BMP2K | --- not yet entered --- |
| H. sapiens (human) | CAMK2G | --- not yet entered --- |
| H. sapiens (human) | CD7 | --- not yet entered --- |
| H. sapiens (human) | CDKN2B | --- not yet entered --- |
| H. sapiens (human) | CERK | --- not yet entered --- |
| H. sapiens (human) | DCK | --- not yet entered --- |
| H. sapiens (human) | DMPK | --- not yet entered --- |
| H. sapiens (human) | DTYMK | --- not yet entered --- |
| H. sapiens (human) | DUSP10 | --- not yet entered --- |
| H. sapiens (human) | EPHA4 | Protein modification |
| H. sapiens (human) | FGFR4 | Cell membrane / wall |
| H. sapiens (human) | GAP43 | Cell membrane / wall |
| H. sapiens (human) | IKBKAP | Signaling |
| H. sapiens (human) | IRAK1 | --- not yet entered --- |
| H. sapiens (human) | JAK2 | Chromatin organization |
| H. sapiens (human) | MALT1 | --- not yet entered --- |
| H. sapiens (human) | MAP3K1 | Signaling |
| H. sapiens (human) | MAP3K2 | --- not yet entered --- |
| H. sapiens (human) | MAP3K3 | --- not yet entered --- |
| H. sapiens (human) | MAP3K5 | Mitochondria |
| H. sapiens (human) | MAP4K3 | --- not yet entered --- |
| H. sapiens (human) | MAPK3 | --- not yet entered --- |
| H. sapiens (human) | MAPK8 | DNA replication and repair |
| H. sapiens (human) | MAPKAPK5 | --- not yet entered --- |
| H. sapiens (human) | MET | --- not yet entered --- |
| H. sapiens (human) | MPZL1 | Cell membrane / wall |
| H. sapiens (human) | NEK4 | --- not yet entered --- |
| H. sapiens (human) | NTRK1 | --- not yet entered --- |
| H. sapiens (human) | PCK1 | --- not yet entered --- |
| H. sapiens (human) | PI4K2B | Cell membrane / wall |
| H. sapiens (human) | PIK3C2B | --- not yet entered --- |
| H. sapiens (human) | PKIB | --- not yet entered --- |
| H. sapiens (human) | PPP1R1B | --- not yet entered --- |
| H. sapiens (human) | PRKAR2A | Cell membrane / wall |
| H. sapiens (human) | PRKAR2B | Cell membrane / wall |
| H. sapiens (human) | PRKCQ | --- not yet entered --- |
| H. sapiens (human) | PRKCSH | --- not yet entered --- |
| H. sapiens (human) | PRKD2 | --- not yet entered --- |
| H. sapiens (human) | PTPRG | --- not yet entered --- |
| H. sapiens (human) | RAPGEF3 | --- not yet entered --- |
| H. sapiens (human) | ROCK1 | Cell membrane / wall |
| H. sapiens (human) | ROCK2 | --- not yet entered --- |
| H. sapiens (human) | RPS6KA4 | Chromatin organization |
| H. sapiens (human) | RPS6KB2 | --- not yet entered --- |
| H. sapiens (human) | SEPHS2 | --- not yet entered --- |
| H. sapiens (human) | SOCS1 | --- not yet entered --- |
| H. sapiens (human) | SPA17 | --- not yet entered --- |
| H. sapiens (human) | SPHK2 | --- not yet entered --- |
| H. sapiens (human) | SRC | Mitochondria |
| H. sapiens (human) | STK16 | Cell membrane / wall |
| H. sapiens (human) | STK19 | Chromatin organization |
| H. sapiens (human) | TNIK | Protein modification |
| H. sapiens (human) | UGP2 | --- not yet entered --- |
| H. sapiens (human) | YES1 | --- not yet entered --- |
| H. sapiens (human) | STK26 | --- not yet entered --- |
| H. sapiens (human) | STRADA | --- not yet entered --- |
| H. sapiens (human) | MAPK15 | --- not yet entered --- |
| H. sapiens (human) | COQ8B | Mitochondria |
| H. sapiens (human) | CDK20 | Cell membrane / wall |
| H. sapiens (human) | PAK5 | --- not yet entered --- |
| H. sapiens (human) | MAST1 | --- not yet entered --- |
| H. sapiens (human) | BRSK2 | --- not yet entered --- |
| H. sapiens (human) | IP6K3 | --- not yet entered --- |
| H. sapiens (human) | EPHA6 | --- not yet entered --- |
| H. sapiens (human) | APPL1 | --- not yet entered --- |
| H. sapiens (human) | IQCH | --- not yet entered --- |
| H. sapiens (human) | PPP1R17 | --- not yet entered --- |
| H. sapiens (human) | RPRD1A | --- not yet entered --- |
| H. sapiens (human) | MAP2K7 | Mitochondria |
| H. sapiens (human) | TPRXL | Cell differentiation |
| H. sapiens (human) | ACAD10 | --- not yet entered --- |
| H. sapiens (human) | NADK | --- not yet entered --- |
| H. sapiens (human) | LOC100421108 | --- not yet entered --- |
| H. sapiens (human) | ILF3 | DNA replication and repair |
| H. sapiens (human) | BASP1 | Cell membrane / wall |
| H. sapiens (human) | ATP5O | Mitochondria |
| H. sapiens (human) | DLAT | Mitochondria |
| H. sapiens (human) | GLG1 | --- not yet entered --- |
| H. sapiens (human) | DAP3 | Mitochondria |
| H. sapiens (human) | RPN2 | --- not yet entered --- |
| H. sapiens (human) | PC | Mitochondria |
| H. sapiens (human) | CAPRIN1 | Cell membrane / wall |
| H. sapiens (human) | G3BP1 | --- not yet entered --- |
| H. sapiens (human) | IGF2BP1 | RNA transcription and processing |
| H. sapiens (human) | ATP1A1 | Cell membrane / wall |
| H. sapiens (human) | PAPSS1 | --- not yet entered --- |
| H. sapiens (human) | FBXO22 | --- not yet entered --- |
| H. sapiens (human) | HNRNPC | RNA transcription and processing |
| H. sapiens (human) | IGF2BP2 | --- not yet entered --- |
| H. sapiens (human) | ATP5F1 | Mitochondria |
| H. sapiens (human) | IGF2BP3 | --- not yet entered --- |
| H. sapiens (human) | SLC25A5 | Mitochondria |
| H. sapiens (human) | MARCKS | Cellular structure |
| H. sapiens (human) | PGRMC1 | Cell membrane / wall |
| H. sapiens (human) | RPN1 | --- not yet entered --- |
| H. sapiens (human) | EIF5B | --- not yet entered --- |
| H. sapiens (human) | ATP5A1 | Mitochondria |
| H. sapiens (human) | RPL26 | --- not yet entered --- |
| H. sapiens (human) | RPL27 | --- not yet entered --- |
| H. sapiens (human) | RPLP1 | --- not yet entered --- |
| H. sapiens (human) | ABCF1 | --- not yet entered --- |
| H. sapiens (human) | DDX3X | DNA replication and repair |
| H. sapiens (human) | NPM1 | DNA replication and repair |
| H. sapiens (human) | FARSA | --- not yet entered --- |
| H. sapiens (human) | RPL27A | --- not yet entered --- |
| H. sapiens (human) | RPS20 | --- not yet entered --- |
| H. sapiens (human) | ATP2A2 | Mitochondria |
| H. sapiens (human) | KRT10 | --- not yet entered --- |
| H. sapiens (human) | NONO | DNA replication and repair |
| H. sapiens (human) | SLC3A2 | Cell membrane / wall |
| H. sapiens (human) | ATP5B | Mitochondria |
| H. sapiens (human) | RPL21 | --- not yet entered --- |
| H. sapiens (human) | FKBP3 | --- not yet entered --- |
| H. sapiens (human) | AIFM1 | Mitochondria |
| H. sapiens (human) | HNRNPCL1 | --- not yet entered --- |
| H. sapiens (human) | GIGYF2 | --- not yet entered --- |
| H. sapiens (human) | RPL23 | --- not yet entered --- |
| H. sapiens (human) | DDX17 | RNA transcription and processing |
| H. sapiens (human) | DLST | Mitochondria |
| H. sapiens (human) | CDC37 | Protein synthesis and degradation |
| H. sapiens (human) | RPL23A | --- not yet entered --- |
| H. sapiens (human) | NAA15 | --- not yet entered --- |
| H. sapiens (human) | CKAP4 | Cell membrane / wall |
| H. sapiens (human) | APEH | --- not yet entered --- |
| H. sapiens (human) | DHX9 | RNA transcription and processing |
| H. sapiens (human) | ATP6V1A | --- not yet entered --- |
| H. sapiens (human) | RPL31 | --- not yet entered --- |
| H. sapiens (human) | RPL36 | --- not yet entered --- |
| H. sapiens (human) | PDAP1 | --- not yet entered --- |
| H. sapiens (human) | RPL10 | --- not yet entered --- |
| H. sapiens (human) | EIF5 | --- not yet entered --- |
| H. sapiens (human) | TPT1 | --- not yet entered --- |
| H. sapiens (human) | SYNCRIP | RNA transcription and processing |
| H. sapiens (human) | ILF2 | --- not yet entered --- |
| H. sapiens (human) | RPLP2 | --- not yet entered --- |
| H. sapiens (human) | RPS7 | --- not yet entered --- |
| H. sapiens (human) | RPL12 | --- not yet entered --- |
| H. sapiens (human) | HNRNPA0 | --- not yet entered --- |
| H. sapiens (human) | YBX1 | DNA replication and repair |
| H. sapiens (human) | RPL26L1 | --- not yet entered --- |
| H. sapiens (human) | RPL17 | --- not yet entered --- |
| H. sapiens (human) | CTNNA1 | --- not yet entered --- |
| H. sapiens (human) | IPO8 | --- not yet entered --- |
| H. sapiens (human) | WDHD1 | DNA replication and repair |
| H. sapiens (human) | REEP5 | --- not yet entered --- |
| H. sapiens (human) | GARS | --- not yet entered --- |
| H. sapiens (human) | PPP1R7 | --- not yet entered --- |
| H. sapiens (human) | PHGDH | --- not yet entered --- |
| H. sapiens (human) | ALDH2 | Mitochondria |
| H. sapiens (human) | ANXA1 | Cell membrane / wall |
| H. sapiens (human) | SERPINH1 | Metabolism |
| H. sapiens (human) | PSPH | --- not yet entered --- |
| H. sapiens (human) | PSAT1 | --- not yet entered --- |
| H. sapiens (human) | OXCT1 | Mitochondria |
| H. sapiens (human) | GSPT1 | --- not yet entered --- |
| H. sapiens (human) | ASNS | --- not yet entered --- |
| H. sapiens (human) | ALDH1A1 | --- not yet entered --- |
| H. sapiens (human) | PUS7 | --- not yet entered --- |
| H. sapiens (human) | PKLR | --- not yet entered --- |
| H. sapiens (human) | ABCC8 | --- not yet entered --- |
| H. sapiens (human) | UROS | --- not yet entered --- |
| H. sapiens (human) | ABCC2 | --- not yet entered --- |
| H. sapiens (human) | PAFAH1B1 | --- not yet entered --- |
| H. sapiens (human) | CFTR | --- not yet entered --- |
| H. sapiens (human) | POR | --- not yet entered --- |
| H. sapiens (human) | RPL34 | --- not yet entered --- |
| H. sapiens (human) | CHTF8 | --- not yet entered --- |
| H. sapiens (human) | RABL2B | --- not yet entered --- |
| H. sapiens (human) | SP140 | Chromatin organization |
| H. sapiens (human) | ACTR2 | --- not yet entered --- |
| H. sapiens (human) | VPS54 | --- not yet entered --- |
| H. sapiens (human) | WDR31 | --- not yet entered --- |
| H. sapiens (human) | LRRC18 | --- not yet entered --- |
| H. sapiens (human) | RPS4X | --- not yet entered --- |
| H. sapiens (human) | RPS4Y1 | --- not yet entered --- |
| H. sapiens (human) | CNOT4 | --- not yet entered --- |
| H. sapiens (human) | LGMN | --- not yet entered --- |
| H. sapiens (human) | TCHHL1 | --- not yet entered --- |
| H. sapiens (human) | USP20 | --- not yet entered --- |
| H. sapiens (human) | TRAPPC2 | --- not yet entered --- |
| H. sapiens (human) | CREB5 | --- not yet entered --- |
| H. sapiens (human) | NAA30 | --- not yet entered --- |
| H. sapiens (human) | FOXP4 | --- not yet entered --- |
| H. sapiens (human) | FOXP1 | --- not yet entered --- |
| H. sapiens (human) | RPS10 | --- not yet entered --- |
| H. sapiens (human) | USP21 | Chromatin organization |
| H. sapiens (human) | RPS11 | --- not yet entered --- |
| H. sapiens (human) | FPGS | Mitochondria |
| H. sapiens (human) | RPS15A | --- not yet entered --- |
| H. sapiens (human) | RPS16 | --- not yet entered --- |
| H. sapiens (human) | RPS17 | --- not yet entered --- |
| H. sapiens (human) | RPS19 | --- not yet entered --- |
| H. sapiens (human) | ABCC5 | --- not yet entered --- |
| H. sapiens (human) | RPS21 | --- not yet entered --- |
| H. sapiens (human) | ARF1 | Cell membrane / wall |
| H. sapiens (human) | LRRC63 | --- not yet entered --- |
| H. sapiens (human) | BRMS1 | --- not yet entered --- |
| H. sapiens (human) | RPS23 | --- not yet entered --- |
| H. sapiens (human) | RPS14 | --- not yet entered --- |
| H. sapiens (human) | CHN1 | --- not yet entered --- |
| H. sapiens (human) | ARRDC2 | --- not yet entered --- |
| H. sapiens (human) | RPS27 | --- not yet entered --- |
| H. sapiens (human) | RPS28 | --- not yet entered --- |
| H. sapiens (human) | CALML4 | --- not yet entered --- |
| H. sapiens (human) | RRM1 | --- not yet entered --- |
| H. sapiens (human) | CSTF3 | --- not yet entered --- |
| H. sapiens (human) | ZNHIT3 | --- not yet entered --- |
| H. sapiens (human) | CABP1 | Cell membrane / wall |
| H. sapiens (human) | ARL4A | Cell membrane / wall |
| H. sapiens (human) | FOXK1 | RNA transcription and processing |
| H. sapiens (human) | PARL | Mitochondria |
| H. sapiens (human) | PRPS2 | --- not yet entered --- |
| H. sapiens (human) | RPS4Y2 | --- not yet entered --- |
| H. sapiens (human) | PNCK | --- not yet entered --- |
| H. sapiens (human) | CHN2 | --- not yet entered --- |
| H. sapiens (human) | MLH3 | DNA replication and repair |
| H. sapiens (human) | ACTR3B | --- not yet entered --- |
| H. sapiens (human) | HSP90AA2P | --- not yet entered --- |
| H. sapiens (human) | INCENP | Cell cycle |
| H. sapiens (human) | UEVLD | --- not yet entered --- |
| H. sapiens (human) | GDAP1 | Mitochondria |
| H. sapiens (human) | XRN1 | DNA replication and repair |
| H. sapiens (human) | WDR38 | --- not yet entered --- |
| H. sapiens (human) | SLC7A6 | --- not yet entered --- |
| H. sapiens (human) | POMT1 | --- not yet entered --- |
| H. sapiens (human) | PNKD | --- not yet entered --- |
| H. sapiens (human) | UBXN2B | Cell membrane / wall |
| H. sapiens (human) | CDC16 | --- not yet entered --- |
| H. sapiens (human) | ABCC6 | --- not yet entered --- |
| H. sapiens (human) | ARHGAP9 | --- not yet entered --- |
| H. sapiens (human) | KDM6B | Chromatin organization |
| H. sapiens (human) | LRRIQ4 | --- not yet entered --- |
| H. sapiens (human) | ASPG | --- not yet entered --- |
| H. sapiens (human) | ARRDC5 | --- not yet entered --- |
| H. sapiens (human) | ISCA1P1 | --- not yet entered --- |
| H. sapiens (human) | RB1CC1 | DNA replication and repair |
| H. sapiens (human) | CDK10 | --- not yet entered --- |
| H. sapiens (human) | ZGRF1 | --- not yet entered --- |
| H. sapiens (human) | PSMB11 | --- not yet entered --- |
| H. sapiens (human) | RAP1GDS1 | --- not yet entered --- |
| H. sapiens (human) | ELOVL7 | Cell membrane / wall |
| H. sapiens (human) | ASH2L | Chromatin organization |
| H. sapiens (human) | ABCC4 | --- not yet entered --- |
| H. sapiens (human) | CDC27 | --- not yet entered --- |
| H. sapiens (human) | FOXP3 | Chromatin organization |
| H. sapiens (human) | HHAT | --- not yet entered --- |
| H. sapiens (human) | BCKDK | --- not yet entered --- |
| H. sapiens (human) | ADK | --- not yet entered --- |
| H. sapiens (human) | PPP6C | --- not yet entered --- |
| H. sapiens (human) | ADSS | --- not yet entered --- |
| H. sapiens (human) | RACGAP1 | Cell membrane / wall |
| H. sapiens (human) | SLC7A7 | --- not yet entered --- |
| H. sapiens (human) | SLC25A19 | Mitochondria |
| H. sapiens (human) | GINS3 | DNA replication and repair |
| H. sapiens (human) | SLC7A9 | --- not yet entered --- |
| H. sapiens (human) | USP8 | Cell membrane / wall |
| H. sapiens (human) | SMARCA4 | Chromatin organization |
| H. sapiens (human) | ATF7 | --- not yet entered --- |
| H. sapiens (human) | MOV10 | DNA replication and repair |
| H. sapiens (human) | KARS | --- not yet entered --- |
| H. sapiens (human) | DNAJA4 | --- not yet entered --- |
| H. sapiens (human) | AHCYL2 | --- not yet entered --- |
| H. sapiens (human) | ELOVL6 | Cell membrane / wall |
| H. sapiens (human) | PSMB5 | --- not yet entered --- |
| H. sapiens (human) | HYOU1 | Protein synthesis and degradation |
| H. sapiens (human) | RPS27A | DNA replication and repair |
| H. sapiens (human) | ARHGAP26 | --- not yet entered --- |
| H. sapiens (human) | RAB29 | --- not yet entered --- |
| H. sapiens (human) | DOLPP1 | --- not yet entered --- |
| H. sapiens (human) | PTGES3L-AARSD1 | --- not yet entered --- |
| H. sapiens (human) | FZR1 | DNA replication and repair |
| H. sapiens (human) | ANAPC7 | --- not yet entered --- |
| H. sapiens (human) | PDK3 | Mitochondria |
| H. sapiens (human) | TGFBRAP1 | Cell membrane / wall |
| H. sapiens (human) | SEC61A2 | Cell membrane / wall |
| H. sapiens (human) | UBTFL1 | --- not yet entered --- |
| H. sapiens (human) | NDOR1 | --- not yet entered --- |
| H. sapiens (human) | ABCC3 | --- not yet entered --- |
| H. sapiens (human) | EFCAB3 | --- not yet entered --- |
| H. sapiens (human) | SIN3A | Chromatin organization |
| H. sapiens (human) | CDC20B | --- not yet entered --- |
| H. sapiens (human) | RNF222 | --- not yet entered --- |
| H. sapiens (human) | ARHGAP27 | --- not yet entered --- |
| H. sapiens (human) | MPND | --- not yet entered --- |
| H. sapiens (human) | POC1A | --- not yet entered --- |
| H. sapiens (human) | MOV10L1 | --- not yet entered --- |
| H. sapiens (human) | TUBB8 | --- not yet entered --- |
| H. sapiens (human) | LRCH1 | --- not yet entered --- |
| H. sapiens (human) | DHX16 | --- not yet entered --- |
| H. sapiens (human) | CDK5 | Chromatin organization |
| H. sapiens (human) | ACTR3C | --- not yet entered --- |
| H. sapiens (human) | TSPY4 | --- not yet entered --- |
| H. sapiens (human) | RAB7B | --- not yet entered --- |
| H. sapiens (human) | CALM1 | Signaling |
| H. sapiens (human) | EIF2B3 | --- not yet entered --- |
| H. sapiens (human) | CDK16 | --- not yet entered --- |
| H. sapiens (human) | CDK17 | Chromatin organization |
| H. sapiens (human) | ZNHIT6 | --- not yet entered --- |
| H. sapiens (human) | FOXO4 | --- not yet entered --- |
| H. sapiens (human) | EFCAB9 | --- not yet entered --- |
| H. sapiens (human) | FOXP2 | --- not yet entered --- |
| H. sapiens (human) | PPP2R1B | --- not yet entered --- |
| H. sapiens (human) | VAMP4 | Cell membrane / wall |
| H. sapiens (human) | C1D | RNA transcription and processing |
| H. sapiens (human) | DHX35 | --- not yet entered --- |
| H. sapiens (human) | TNPO3 | --- not yet entered --- |
| H. sapiens (human) | RAB9A | --- not yet entered --- |
| H. sapiens (human) | TSPY1 | --- not yet entered --- |
| H. sapiens (human) | FOXJ3 | --- not yet entered --- |
| H. sapiens (human) | ABCC10 | --- not yet entered --- |
| H. sapiens (human) | ARHGAP23 | --- not yet entered --- |
| H. sapiens (human) | DHX33 | RNA transcription and processing |
| H. sapiens (human) | POC1B | --- not yet entered --- |
| H. sapiens (human) | PDK2 | Mitochondria |
| H. sapiens (human) | RNF7 | --- not yet entered --- |
| H. sapiens (human) | RPS10-NUDT3 | --- not yet entered --- |
| H. sapiens (human) | WBSCR22 | Chromatin organization |
| H. sapiens (human) | PRPS1 | --- not yet entered --- |
| H. sapiens (human) | COG3 | --- not yet entered --- |
| H. sapiens (human) | EIF4A1 | --- not yet entered --- |
| H. sapiens (human) | BNIP1 | Mitochondria |
| H. sapiens (human) | NSFL1C | --- not yet entered --- |
| H. sapiens (human) | RNF40 | Chromatin organization |
| H. sapiens (human) | ATAD2B | --- not yet entered --- |
| H. sapiens (human) | AHCYL1 | DNA replication and repair |
| H. sapiens (human) | ELOVL5 | Cell membrane / wall |
| H. sapiens (human) | FOXM1 | DNA replication and repair |
| H. sapiens (human) | HIP1 | --- not yet entered --- |
| H. sapiens (human) | TSPY8 | --- not yet entered --- |
| H. sapiens (human) | USP2 | --- not yet entered --- |
| H. sapiens (human) | PRPSAP2 | --- not yet entered --- |
| H. sapiens (human) | LRCH2 | --- not yet entered --- |
| H. sapiens (human) | PRPF4 | --- not yet entered --- |
| H. sapiens (human) | CDC20 | Cell membrane / wall |
| H. sapiens (human) | PIK3CB | Cell membrane / wall |
| H. sapiens (human) | ATF2 | Mitochondria |
| H. sapiens (human) | ELOVL1 | Cell membrane / wall |
| H. sapiens (human) | USP39 | --- not yet entered --- |
| H. sapiens (human) | GDAP1L1 | --- not yet entered --- |
| H. sapiens (human) | PAF1 | Chromatin organization |
| H. sapiens (human) | DNAJC6 | --- not yet entered --- |
| H. sapiens (human) | UTY | Chromatin organization |
| H. sapiens (human) | CLPB | --- not yet entered --- |
| H. sapiens (human) | CDK8 | RNA transcription and processing |
| H. sapiens (human) | CDK9 | DNA replication and repair |
| H. sapiens (human) | LPIN1 | Mitochondria |
| H. sapiens (human) | AARSD1 | --- not yet entered --- |
| H. sapiens (human) | CDK15 | --- not yet entered --- |
| H. sapiens (human) | SLC7A8 | Metabolism |
| H. sapiens (human) | ARPC5 | --- not yet entered --- |
| H. sapiens (human) | PSMD11 | DNA replication and repair |
| H. sapiens (human) | ARHGAP12 | --- not yet entered --- |
| H. sapiens (human) | TFAM | Mitochondria |
| H. sapiens (human) | ABCB7 | Mitochondria |
| H. sapiens (human) | PSMD6 | DNA replication and repair |
| H. sapiens (human) | RRAGC | --- not yet entered --- |
| H. sapiens (human) | AAR2 | --- not yet entered --- |
| H. sapiens (human) | ISCA2 | Mitochondria |
| H. sapiens (human) | TRAP1 | Mitochondria |
| H. sapiens (human) | ACTR3 | --- not yet entered --- |
| H. sapiens (human) | PDK1 | Mitochondria |
| H. sapiens (human) | RHBDL1 | Cell membrane / wall |
| H. sapiens (human) | VPS36 | --- not yet entered --- |
| H. sapiens (human) | SIGMAR1 | Mitochondria |
| H. sapiens (human) | PIK3CG | Cell membrane / wall |
| H. sapiens (human) | ARL4C | Cell membrane / wall |
| H. sapiens (human) | TSPY10 | --- not yet entered --- |
| H. sapiens (human) | GAPVD1 | Cell membrane / wall |
| H. sapiens (human) | EIF3J | --- not yet entered --- |
| H. sapiens (human) | LEO1 | Chromatin organization |
| H. sapiens (human) | HSPH1 | Protein synthesis and degradation |
| H. sapiens (human) | ANAPC4 | --- not yet entered --- |
| H. sapiens (human) | GAK | --- not yet entered --- |
| H. sapiens (human) | RABGEF1 | Cell membrane / wall |
| H. sapiens (human) | CDK14 | --- not yet entered --- |
| H. sapiens (human) | SETSIP | --- not yet entered --- |
| H. sapiens (human) | TEX2 | --- not yet entered --- |
| H. sapiens (human) | PIK3C2G | --- not yet entered --- |
| H. sapiens (human) | GMIP | --- not yet entered --- |
| H. sapiens (human) | PHLPP2 | --- not yet entered --- |
| H. sapiens (human) | TUBB4A | --- not yet entered --- |
| H. sapiens (human) | SMARCA2 | Chromatin organization |
| H. sapiens (human) | HAGHL | --- not yet entered --- |
| H. sapiens (human) | FOXO6 | --- not yet entered --- |
| H. sapiens (human) | CDK11B | --- not yet entered --- |
| H. sapiens (human) | KDM6A | Chromatin organization |
| H. sapiens (human) | DARS | --- not yet entered --- |
| H. sapiens (human) | VAMP1 | Cell membrane / wall |
| H. sapiens (human) | SIN3B | Chromatin organization |
| H. sapiens (human) | DDX46 | --- not yet entered --- |
| H. sapiens (human) | CABP4 | --- not yet entered --- |
| H. sapiens (human) | SYDE1 | --- not yet entered --- |
| H. sapiens (human) | ZMIZ2 | --- not yet entered --- |
| H. sapiens (human) | CDK19 | --- not yet entered --- |
| H. sapiens (human) | VPS39 | Cell membrane / wall |
| H. sapiens (human) | LPIN3 | --- not yet entered --- |
| H. sapiens (human) | DHX8 | --- not yet entered --- |
| H. sapiens (human) | HIP1R | --- not yet entered --- |
| H. sapiens (human) | TUBB6 | --- not yet entered --- |
| H. sapiens (human) | PTPN23 | --- not yet entered --- |
| H. sapiens (human) | HMG20A | Chromatin organization |
| H. sapiens (human) | RHBDL2 | Cell membrane / wall |
| H. sapiens (human) | CALM2 | --- not yet entered --- |
| H. sapiens (human) | RABL2A | --- not yet entered --- |
| H. sapiens (human) | CSTF2 | --- not yet entered --- |
| H. sapiens (human) | PIK3C3 | Cell membrane / wall |
| H. sapiens (human) | SP140L | --- not yet entered --- |
| H. sapiens (human) | GSTZ1 | --- not yet entered --- |
| H. sapiens (human) | CDK11A | --- not yet entered --- |
| H. sapiens (human) | TXNIP | --- not yet entered --- |
| H. sapiens (human) | PSMD12 | DNA replication and repair |
| H. sapiens (human) | SNF8 | RNA transcription and processing |
| H. sapiens (human) | HSPA4L | Protein synthesis and degradation |
| H. sapiens (human) | XRN2 | RNA transcription and processing |
| H. sapiens (human) | ARRDC1 | --- not yet entered --- |
| H. sapiens (human) | TRAPPC4 | --- not yet entered --- |
| H. sapiens (human) | CABP2 | --- not yet entered --- |
| H. sapiens (human) | ATG16L2 | --- not yet entered --- |
| H. sapiens (human) | CAMKV | --- not yet entered --- |
| H. sapiens (human) | ADSSL1 | --- not yet entered --- |
| H. sapiens (human) | GPS1 | --- not yet entered --- |
| H. sapiens (human) | SUZ12 | Chromatin organization |
| H. sapiens (human) | AHSA2 | Protein synthesis and degradation |
| H. sapiens (human) | PIK3C2A | Cell membrane / wall |
| H. sapiens (human) | CNPPD1 | --- not yet entered --- |
| H. sapiens (human) | AHSA1 | Protein synthesis and degradation |
| H. sapiens (human) | NAA35 | --- not yet entered --- |
| H. sapiens (human) | PIAS2 | --- not yet entered --- |
| H. sapiens (human) | ARRDC3 | Cell membrane / wall |
| H. sapiens (human) | CALM3 | --- not yet entered --- |
| H. sapiens (human) | DAW1 | --- not yet entered --- |
| H. sapiens (human) | PRKD1 | --- not yet entered --- |
| H. sapiens (human) | VAMP2 | Cell membrane / wall |
| H. sapiens (human) | RHBDL3 | Cell membrane / wall |
| H. sapiens (human) | CALML6 | --- not yet entered --- |
| H. sapiens (human) | PRPSAP1 | --- not yet entered --- |
| H. sapiens (human) | PDXK | --- not yet entered --- |
| H. sapiens (human) | FOXF2 | --- not yet entered --- |
| H. sapiens (human) | FOXJ1 | --- not yet entered --- |
| H. sapiens (human) | FOXO3 | RNA transcription and processing |
| H. sapiens (human) | AARS | --- not yet entered --- |
| H. sapiens (human) | TRIM23 | --- not yet entered --- |
| H. sapiens (human) | ARL4D | Cell membrane / wall |
| H. sapiens (human) | ARF6 | --- not yet entered --- |
| H. sapiens (human) | EIF4A2 | --- not yet entered --- |
| H. sapiens (human) | FOXO1 | DNA replication and repair |
| H. sapiens (human) | HSPA4 | --- not yet entered --- |
| H. sapiens (human) | JUNB | RNA transcription and processing |
| H. sapiens (human) | OGG1 | DNA replication and repair |
| H. sapiens (human) | OPHN1 | --- not yet entered --- |
| H. sapiens (human) | CDK18 | --- not yet entered --- |
| H. sapiens (human) | PDK4 | Mitochondria |
| H. sapiens (human) | PFDN1 | Protein synthesis and degradation |
| H. sapiens (human) | POLR2G | RNA transcription and processing |
| H. sapiens (human) | PSMC3 | DNA replication and repair |
| H. sapiens (human) | PSMD8 | DNA replication and repair |
| H. sapiens (human) | SMARCC1 | Chromatin organization |
| H. sapiens (human) | SMARCE1 | Chromatin organization |
| H. sapiens (human) | TNNC2 | --- not yet entered --- |
| H. sapiens (human) | TNNC1 | Cellular structure |
| H. sapiens (human) | HSP90B1 | DNA replication and repair |
| H. sapiens (human) | TSPYL1 | --- not yet entered --- |
| H. sapiens (human) | SLC7A5 | Cell membrane / wall |
| H. sapiens (human) | PPM1D | --- not yet entered --- |
| H. sapiens (human) | CAMK1 | --- not yet entered --- |
| H. sapiens (human) | CDK13 | RNA transcription and processing |
| H. sapiens (human) | VAMP8 | Cell membrane / wall |
| H. sapiens (human) | FOXH1 | --- not yet entered --- |
| H. sapiens (human) | PSMB8 | --- not yet entered --- |
| H. sapiens (human) | MED14 | RNA transcription and processing |
| H. sapiens (human) | GCSH | Mitochondria |
| H. sapiens (human) | ARHGAP35 | --- not yet entered --- |
| H. sapiens (human) | FOXK2 | --- not yet entered --- |
| H. sapiens (human) | RAB7A | Cell membrane / wall |
| H. sapiens (human) | CDC23 | --- not yet entered --- |
| H. sapiens (human) | VAMP3 | Cell membrane / wall |
| H. sapiens (human) | POLR2D | RNA transcription and processing |
| H. sapiens (human) | SNRNP40 | --- not yet entered --- |
| H. sapiens (human) | MRPL49 | Mitochondria |
| H. sapiens (human) | ABCC1 | --- not yet entered --- |
| H. sapiens (human) | PIK3CD | Cell membrane / wall |
| H. sapiens (human) | MED12 | RNA transcription and processing |
| H. sapiens (human) | GNL1 | --- not yet entered --- |
| H. sapiens (human) | PIGK | --- not yet entered --- |
| H. sapiens (human) | ABCB6 | Mitochondria |
| H. sapiens (human) | ABCC9 | --- not yet entered --- |
| H. sapiens (human) | ARPC2 | --- not yet entered --- |
| H. sapiens (human) | PRKD3 | --- not yet entered --- |
| H. sapiens (human) | PIK3CA | Cell membrane / wall |
| H. sapiens (human) | POLR2I | RNA transcription and processing |
| H. sapiens (human) | TLN1 | Cell membrane / wall |
| H. sapiens (human) | TSG101 | --- not yet entered --- |
| H. sapiens (human) | HMG20B | Chromatin organization |
| H. sapiens (human) | RNASEH2A | DNA replication and repair |
| H. sapiens (human) | PSMC4 | DNA replication and repair |
| H. sapiens (human) | VAMP5 | Cell membrane / wall |
| H. sapiens (human) | PSKH1 | --- not yet entered --- |
| H. sapiens (human) | RAB32 | Mitochondria |
| H. sapiens (human) | RPL10A | --- not yet entered --- |
| H. sapiens (human) | SEC63 | Cell membrane / wall |
| H. sapiens (human) | PIGN | DNA replication and repair |
| H. sapiens (human) | NUFIP1 | --- not yet entered --- |
| H. sapiens (human) | SEC61A1 | Cell membrane / wall |
| H. sapiens (human) | POMT2 | --- not yet entered --- |
| H. sapiens (human) | MRM2 | Mitochondria |
| H. sapiens (human) | ATAD2 | Chromatin organization |
| H. sapiens (human) | PPP2R1A | --- not yet entered --- |
| H. sapiens (human) | RBX1 | DNA replication and repair |
| H. sapiens (human) | SLC7A11 | Cell membrane / wall |
| H. sapiens (human) | PIK3R4 | Cell membrane / wall |
| H. sapiens (human) | MDN1 | Protein synthesis and degradation |
| H. sapiens (human) | PPM1F | --- not yet entered --- |
| H. sapiens (human) | LPIN2 | --- not yet entered --- |
| H. sapiens (human) | IPO13 | --- not yet entered --- |
| H. sapiens (human) | DHX34 | --- not yet entered --- |
| H. sapiens (human) | EIF4A3 | --- not yet entered --- |
| H. sapiens (human) | PPM1E | --- not yet entered --- |
| H. sapiens (human) | USP33 | --- not yet entered --- |
| H. sapiens (human) | TLN2 | --- not yet entered --- |
| H. sapiens (human) | CDK12 | DNA replication and repair |
| H. sapiens (human) | RTF1 | Chromatin organization |
| H. sapiens (human) | CSTF2T | --- not yet entered --- |
| H. sapiens (human) | RRP8 | Chromatin organization |
| H. sapiens (human) | UPF2 | RNA transcription and processing |
| H. sapiens (human) | GPATCH4 | --- not yet entered --- |
| H. sapiens (human) | RPS27L | --- not yet entered --- |
| H. sapiens (human) | MED31 | RNA transcription and processing |
| H. sapiens (human) | NAA20 | --- not yet entered --- |
| H. sapiens (human) | LSM7 | --- not yet entered --- |
| H. sapiens (human) | LSM8 | --- not yet entered --- |
| H. sapiens (human) | VPS28 | --- not yet entered --- |
| H. sapiens (human) | TUBE1 | --- not yet entered --- |
| H. sapiens (human) | DNAJB11 | Protein synthesis and degradation |
| H. sapiens (human) | RAB9B | --- not yet entered --- |
| H. sapiens (human) | USP18 | --- not yet entered --- |
| H. sapiens (human) | CALML5 | --- not yet entered --- |
| H. sapiens (human) | LRRC40 | --- not yet entered --- |
| H. sapiens (human) | ELOVL2 | Cell membrane / wall |
| H. sapiens (human) | UBE2R2 | --- not yet entered --- |
| H. sapiens (human) | IWS1 | Chromatin organization |
| H. sapiens (human) | GPATCH1 | --- not yet entered --- |
| H. sapiens (human) | TSR1 | --- not yet entered --- |
| H. sapiens (human) | PNPO | --- not yet entered --- |
| H. sapiens (human) | DHX32 | --- not yet entered --- |
| H. sapiens (human) | DNAJC11 | --- not yet entered --- |
| H. sapiens (human) | SMU1 | --- not yet entered --- |
| H. sapiens (human) | LSG1 | --- not yet entered --- |
| H. sapiens (human) | FOXJ2 | --- not yet entered --- |
| H. sapiens (human) | ARHGAP15 | --- not yet entered --- |
| H. sapiens (human) | LRRC59 | --- not yet entered --- |
| H. sapiens (human) | WDR5B | --- not yet entered --- |
| H. sapiens (human) | RNF20 | Chromatin organization |
| H. sapiens (human) | SLC7A10 | --- not yet entered --- |
| H. sapiens (human) | ZMIZ1 | --- not yet entered --- |
| H. sapiens (human) | CAMK1G | --- not yet entered --- |
| H. sapiens (human) | HHATL | Cell membrane / wall |
| H. sapiens (human) | AARS2 | Mitochondria |
| H. sapiens (human) | ARHGAP21 | --- not yet entered --- |
| H. sapiens (human) | VPS18 | Cell membrane / wall |
| H. sapiens (human) | RRAGD | --- not yet entered --- |
| H. sapiens (human) | TSPYL4 | --- not yet entered --- |
| H. sapiens (human) | TSPYL2 | --- not yet entered --- |
| H. sapiens (human) | RAB38 | --- not yet entered --- |
| H. sapiens (human) | SUDS3 | Chromatin organization |
| H. sapiens (human) | RPS18 | --- not yet entered --- |
| H. sapiens (human) | TSPY2 | --- not yet entered --- |
| H. sapiens (human) | GIGYF1 | --- not yet entered --- |
| H. sapiens (human) | ELOVL4 | Cell membrane / wall |
| H. sapiens (human) | ARV1 | Cell membrane / wall |
| H. sapiens (human) | UPF3B | --- not yet entered --- |
| H. sapiens (human) | UPF3A | RNA transcription and processing |
| H. sapiens (human) | MRPL34 | Mitochondria |
| H. sapiens (human) | DDX50 | --- not yet entered --- |
| H. sapiens (human) | NOC4L | --- not yet entered --- |
| H. sapiens (human) | DSCC1 | DNA replication and repair |
| H. sapiens (human) | ARHGAP10 | --- not yet entered --- |
| H. sapiens (human) | LRRK1 | --- not yet entered --- |
| H. sapiens (human) | ERMP1 | --- not yet entered --- |
| H. sapiens (human) | ARL14 | --- not yet entered --- |
| H. sapiens (human) | ILKAP | --- not yet entered --- |
| H. sapiens (human) | TUBB1 | --- not yet entered --- |
| H. sapiens (human) | ISCA1 | Mitochondria |
| H. sapiens (human) | ARPC5L | Cellular structure |
| H. sapiens (human) | SYDE2 | --- not yet entered --- |
| H. sapiens (human) | PRPF38A | --- not yet entered --- |
| H. sapiens (human) | BRMS1L | Chromatin organization |
| H. sapiens (human) | VPS25 | RNA transcription and processing |
| H. sapiens (human) | EFCAB7 | --- not yet entered --- |
| H. sapiens (human) | ABCC11 | --- not yet entered --- |
| H. sapiens (human) | LRCH3 | --- not yet entered --- |
| H. sapiens (human) | RHPN2 | --- not yet entered --- |
| H. sapiens (human) | PSKH2 | --- not yet entered --- |
| H. sapiens (human) | ABCC12 | --- not yet entered --- |
| H. sapiens (human) | TSPYL5 | --- not yet entered --- |
| H. sapiens (human) | HELB | DNA replication and repair |
| H. sapiens (human) | RHPN1 | --- not yet entered --- |
| H. sapiens (human) | MED12L | RNA transcription and processing |
| H. sapiens (human) | TADA1 | --- not yet entered --- |
| H. sapiens (human) | EXOSC6 | Chromatin organization |
| H. sapiens (human) | DQX1 | --- not yet entered --- |
| H. sapiens (human) | ARL11 | --- not yet entered --- |
| H. sapiens (human) | SLC7A13 | --- not yet entered --- |
| H. sapiens (human) | FLCN | --- not yet entered --- |
| H. sapiens (human) | ELOVL3 | Cell membrane / wall |
| H. sapiens (human) | LRR1 | --- not yet entered --- |
| H. sapiens (human) | ARHGAP42 | --- not yet entered --- |
| H. sapiens (human) | PPM1K | Mitochondria |
| H. sapiens (human) | NAGS | Mitochondria |
| H. sapiens (human) | PCSK9 | --- not yet entered --- |
| H. sapiens (human) | PRPS1L1 | --- not yet entered --- |
| H. sapiens (human) | TUBB2B | --- not yet entered --- |
| H. sapiens (human) | ARL5B | --- not yet entered --- |
| H. sapiens (human) | ARRDC4 | --- not yet entered --- |
| H. sapiens (human) | PHLPP1 | Cell membrane / wall |
| H. sapiens (human) | NAT16 | --- not yet entered --- |
| H. sapiens (human) | USP50 | --- not yet entered --- |
| H. sapiens (human) | TSEN54 | --- not yet entered --- |
| H. sapiens (human) | ATG16L1 | Mitochondria |
| H. sapiens (human) | CALM1P1 | --- not yet entered --- |
| H. sapiens (human) | HSP90AA4P | --- not yet entered --- |
| H. sapiens (human) | HSP90AA5P | --- not yet entered --- |
| H. sapiens (human) | HSP90AB2P | --- not yet entered --- |
| H. sapiens (human) | HSP90AB3P | --- not yet entered --- |
| H. sapiens (human) | HSP90AB4P | --- not yet entered --- |
| H. sapiens (human) | RPS10P5 | --- not yet entered --- |
| H. sapiens (human) | RPS4XP21 | --- not yet entered --- |
| H. sapiens (human) | TSPY26P | --- not yet entered --- |
| H. sapiens (human) | TSPY6P | --- not yet entered --- |
| H. sapiens (human) | TRAPPC2B | --- not yet entered --- |
| H. sapiens (human) | UBTFL6 | RNA transcription and processing |
| H. sapiens (human) | ARHGAP45 | --- not yet entered --- |
| H. sapiens (human) | FAM197Y5 | --- not yet entered --- |
| H. sapiens (human) | FAM197Y1 | --- not yet entered --- |
| H. sapiens (human) | APAF1 | --- not yet entered --- |
| H. sapiens (human) | TEX13A | --- not yet entered --- |
| H. sapiens (human) | TEX13B | --- not yet entered --- |
| H. sapiens (human) | PABPC1L2A | --- not yet entered --- |
| H. sapiens (human) | PABPC1L2B | --- not yet entered --- |
| H. sapiens (human) | SART3 | DNA replication and repair |
| H. sapiens (human) | PPM1N | --- not yet entered --- |
| H. sapiens (human) | AMPD1 | --- not yet entered --- |
| H. sapiens (human) | AMPD3 | --- not yet entered --- |
| H. sapiens (human) | GMDS | --- not yet entered --- |
| H. sapiens (human) | UAP1L1 | --- not yet entered --- |
| H. sapiens (human) | STX10 | --- not yet entered --- |
| H. sapiens (human) | STX6 | --- not yet entered --- |
| H. sapiens (human) | STX8 | Cell membrane / wall |
| H. sapiens (human) | PSMA1 | --- not yet entered --- |
| H. sapiens (human) | CNOT2 | --- not yet entered --- |
| H. sapiens (human) | FXN | Mitochondria |
| H. sapiens (human) | TRMT5 | Mitochondria |
| H. sapiens (human) | DMAP1 | DNA replication and repair |
| H. sapiens (human) | MED13 | RNA transcription and processing |
| H. sapiens (human) | MED13L | RNA transcription and processing |
| H. sapiens (human) | BET1 | Cell membrane / wall |
| H. sapiens (human) | BET1L | Cell membrane / wall |
| H. sapiens (human) | SLC25A33 | DNA replication and repair |
| H. sapiens (human) | SLC25A36 | Mitochondria |
| H. sapiens (human) | DHDDS | --- not yet entered --- |
| H. sapiens (human) | PLCB1 | --- not yet entered --- |
| H. sapiens (human) | PLCB2 | --- not yet entered --- |
| H. sapiens (human) | PLCB3 | --- not yet entered --- |
| H. sapiens (human) | PLCB4 | --- not yet entered --- |
| H. sapiens (human) | PLCD1 | --- not yet entered --- |
| H. sapiens (human) | PLCD3 | --- not yet entered --- |
| H. sapiens (human) | PLCD4 | --- not yet entered --- |
| H. sapiens (human) | PLCE1 | --- not yet entered --- |
| H. sapiens (human) | PLCG1 | --- not yet entered --- |
| H. sapiens (human) | PLCG2 | --- not yet entered --- |
| H. sapiens (human) | PLCH1 | --- not yet entered --- |
| H. sapiens (human) | PLCH2 | --- not yet entered --- |
| H. sapiens (human) | PLCL1 | --- not yet entered --- |
| H. sapiens (human) | PLCL2 | --- not yet entered --- |
| H. sapiens (human) | PLCZ1 | Cell membrane / wall |
| H. sapiens (human) | PGS1 | Mitochondria |
| H. sapiens (human) | PDS5A | DNA replication and repair |
| H. sapiens (human) | PDS5B | DNA replication and repair |
| H. sapiens (human) | MRPS11 | Mitochondria |
| H. sapiens (human) | RIDA | --- not yet entered --- |
| H. sapiens (human) | CSNK1A1 | --- not yet entered --- |
| H. sapiens (human) | CSNK1A1L | --- not yet entered --- |
| H. sapiens (human) | CSNK1D | DNA replication and repair |
| H. sapiens (human) | CSNK1E | --- not yet entered --- |
| H. sapiens (human) | CSNK1G1 | --- not yet entered --- |
| H. sapiens (human) | CSNK1G2 | --- not yet entered --- |
| H. sapiens (human) | CSNK1G3 | --- not yet entered --- |
| H. sapiens (human) | TTBK1 | --- not yet entered --- |
| H. sapiens (human) | TTBK2 | --- not yet entered --- |
| H. sapiens (human) | VRK1 | Chromatin organization |
| H. sapiens (human) | VRK2 | Chromatin organization |
| H. sapiens (human) | VRK3 | --- not yet entered --- |
| H. sapiens (human) | ANAPC1 | --- not yet entered --- |
| H. sapiens (human) | ARHGEF28 | --- not yet entered --- |
| H. sapiens (human) | CENPO | --- not yet entered --- |
| H. sapiens (human) | CMTR1 | --- not yet entered --- |
| H. sapiens (human) | EHMT1 | Chromatin organization |
| H. sapiens (human) | KCTD16 | --- not yet entered --- |
| H. sapiens (human) | MASTL | Chromatin organization |
| H. sapiens (human) | NCAPD3 | --- not yet entered --- |
| H. sapiens (human) | PHAX | --- not yet entered --- |
| H. sapiens (human) | TBX15 | --- not yet entered --- |
| H. sapiens (human) | TENM3 | --- not yet entered --- |
| H. sapiens (human) | AHR | RNA transcription and processing |
| H. sapiens (human) | ATF1 | RNA transcription and processing |
| H. sapiens (human) | FOXL2 | RNA transcription and processing |
| H. sapiens (human) | CEBPA | RNA transcription and processing |
| H. sapiens (human) | FOXD2 | --- not yet entered --- |
| H. sapiens (human) | FOSB | RNA transcription and processing |
| H. sapiens (human) | FOSL2 | RNA transcription and processing |
| H. sapiens (human) | GTF2B | RNA transcription and processing |
| H. sapiens (human) | HNF4A | RNA transcription and processing |
| H. sapiens (human) | MAX | RNA transcription and processing |
| H. sapiens (human) | MXI1 | RNA transcription and processing |
| H. sapiens (human) | MYF5 | RNA transcription and processing |
| H. sapiens (human) | MYF6 | Cell differentiation |
| H. sapiens (human) | MYOG | RNA transcription and processing |
| H. sapiens (human) | NFKB2 | RNA transcription and processing |
| H. sapiens (human) | REL | RNA transcription and processing |
| H. sapiens (human) | RELB | RNA transcription and processing |
| H. sapiens (human) | REST | RNA transcription and processing |
| H. sapiens (human) | TAF9 | RNA transcription and processing |
| H. sapiens (human) | TBX2 | RNA transcription and processing |
| H. sapiens (human) | TFAP2C | RNA transcription and processing |
| H. sapiens (human) | USF2 | RNA transcription and processing |
| H. sapiens (human) | XBP1 | RNA transcription and processing |
| H. sapiens (human) | ZNF32 | RNA transcription and processing |
| H. sapiens (human) | FOSL1 | RNA transcription and processing |
| H. sapiens (human) | HMGA2 | RNA transcription and processing |
| H. sapiens (human) | ATF6 | RNA transcription and processing |
| H. sapiens (human) | BCL11A | RNA transcription and processing |
| H. sapiens (human) | MYOCD | RNA transcription and processing |
| H. sapiens (human) | ZBTB9 | RNA transcription and processing |
| H. sapiens (human) | KLF9 | RNA transcription and processing |
| H. sapiens (human) | NFATC1 | RNA transcription and processing |
| H. sapiens (human) | POU5F1 | RNA transcription and processing |
| H. sapiens (human) | PPARG | RNA transcription and processing |
| H. sapiens (human) | ZBTB16 | RNA transcription and processing |
| H. sapiens (human) | ZEB2 | RNA transcription and processing |
| H. sapiens (human) | NR1H3 | Metabolism |
| H. sapiens (human) | TCEAL7 | RNA transcription and processing |
| H. sapiens (human) | ANKLE1 | DNA replication and repair |
| H. sapiens (human) | CCDC155 | Cellular structure |
| H. sapiens (human) | CCNE1 | Cell cycle |
| H. sapiens (human) | CCNE2 | Cell cycle |
| H. sapiens (human) | CCT3 | Protein synthesis and degradation |
| H. sapiens (human) | CCT6A | Protein synthesis and degradation |
| H. sapiens (human) | CCT8 | Protein synthesis and degradation |
| H. sapiens (human) | CDC45 | DNA replication and repair |
| H. sapiens (human) | CERS1 | Metabolism |
| H. sapiens (human) | DHX36 | RNA transcription and processing |
| H. sapiens (human) | DMC1 | DNA replication and repair |
| H. sapiens (human) | DOT1L | Chromatin organization |
| H. sapiens (human) | DPY30 | Chromatin organization |
| H. sapiens (human) | DYDC1 | Metabolism |
| H. sapiens (human) | DYDC2 | --- unknown --- |
| H. sapiens (human) | GNL3L | Telomere biology |
| H. sapiens (human) | GREM1 | --- not yet entered --- |
| H. sapiens (human) | HDAC8 | Chromatin organization |
| H. sapiens (human) | HIST1H3A | Chromatin organization |
| H. sapiens (human) | HIST1H4A | Chromatin organization |
| H. sapiens (human) | HNRNPU | RNA transcription and processing |
| H. sapiens (human) | LEMD3 | Nuclear organization |
| H. sapiens (human) | LSM11 | RNA transcription and processing |
| H. sapiens (human) | MAJIN | Nuclear organization |
| H. sapiens (human) | MEI1 | Cell cycle |
| H. sapiens (human) | NLRP2 | Signaling |
| H. sapiens (human) | NSMCE1 | DNA replication and repair |
| H. sapiens (human) | NUP98 | Nuclear organization |
| H. sapiens (human) | NVL | Telomere biology |
| H. sapiens (human) | PARM1 | RNA transcription and processing |
| H. sapiens (human) | PARP3 | DNA replication and repair |
| H. sapiens (human) | PTGES3 | Protein synthesis and degradation |
| H. sapiens (human) | PURA | RNA transcription and processing |
| H. sapiens (human) | RAD21L1 | Cell cycle |
| H. sapiens (human) | SETX | RNA transcription and processing |
| H. sapiens (human) | SHQ1 | RNA transcription and processing |
| H. sapiens (human) | SIRT2 | Protein modification |
| H. sapiens (human) | SLX1A | DNA replication and repair |
| H. sapiens (human) | SMARCAL1 | DNA replication and repair |
| H. sapiens (human) | SNRPB | RNA transcription and processing |
| H. sapiens (human) | SNRPD3 | RNA transcription and processing |
| H. sapiens (human) | SNRPE | RNA transcription and processing |
| H. sapiens (human) | SPO11 | DNA replication and repair |
| H. sapiens (human) | SUN1 | Nuclear organization |
| H. sapiens (human) | SUN2 | Nuclear organization |
| H. sapiens (human) | TCP1 | Protein synthesis and degradation |
| H. sapiens (human) | TERB1 | Telomere biology |
| H. sapiens (human) | TERB2 | Telomere biology |
| H. sapiens (human) | TFIP11 | RNA transcription and processing |
| H. sapiens (human) | WRAP53 | Telomere biology |
| H. sapiens (human) | TNFRSF6B | Cell death |
| H. sapiens (human) | RMRP | RNA transcription and processing |
| H. sapiens (human) | MLX | --- not yet entered --- |
| H. sapiens (human) | TFE3 | --- not yet entered --- |
| H. sapiens (human) | TFEB | --- not yet entered --- |
| H. sapiens (human) | TFEC | --- not yet entered --- |
| H. sapiens (human) | RBBP4 | DNA replication and repair |
| H. sapiens (human) | RFWD2 | --- not yet entered --- |
| H. sapiens (human) | KLF1 | --- not yet entered --- |
| H. sapiens (human) | KLF12 | --- not yet entered --- |
| H. sapiens (human) | KLF15 | --- not yet entered --- |
| H. sapiens (human) | KLF17 | RNA transcription and processing |
| H. sapiens (human) | KLF3 | --- not yet entered --- |
| H. sapiens (human) | KLF5 | --- not yet entered --- |
| H. sapiens (human) | KLF6 | --- not yet entered --- |
| H. sapiens (human) | KLF7 | --- not yet entered --- |
| H. sapiens (human) | KLF8 | --- not yet entered --- |
| H. sapiens (human) | FOXN1 | --- not yet entered --- |
| H. sapiens (human) | FOXN2 | --- not yet entered --- |
| H. sapiens (human) | FOXN3 | --- not yet entered --- |
| H. sapiens (human) | FOXN4 | --- not yet entered --- |
| H. sapiens (human) | FOXR1 | --- not yet entered --- |
| H. sapiens (human) | GATA1 | --- not yet entered --- |
| H. sapiens (human) | GATA2 | --- not yet entered --- |
| H. sapiens (human) | GATA3 | --- not yet entered --- |
| H. sapiens (human) | GATA4 | --- not yet entered --- |
| H. sapiens (human) | GATA5 | --- not yet entered --- |
| H. sapiens (human) | GATA6 | --- not yet entered --- |
| H. sapiens (human) | TRPS1 | --- not yet entered --- |
| H. sapiens (human) | MECOM | --- not yet entered --- |
| H. sapiens (human) | PRDM16 | --- not yet entered --- |
| H. sapiens (human) | ZNF497 | --- not yet entered --- |
| H. sapiens (human) | ZNF837 | --- not yet entered --- |
| H. sapiens (human) | YY1 | DNA replication and repair |
| H. sapiens (human) | YY2 | --- not yet entered --- |
| H. sapiens (human) | ZFP42 | RNA transcription and processing |
| H. sapiens (human) | JAZF1 | --- not yet entered --- |
| H. sapiens (human) | TEAD1 | --- not yet entered --- |
| H. sapiens (human) | TEAD2 | --- not yet entered --- |
| H. sapiens (human) | TEAD3 | --- not yet entered --- |
| H. sapiens (human) | TEAD4 | --- not yet entered --- |
| H. sapiens (human) | HIST1H3B | Chromatin organization |
| H. sapiens (human) | HIST1H3C | Chromatin organization |
| H. sapiens (human) | HIST1H3D | Chromatin organization |
| H. sapiens (human) | HIST1H3E | Chromatin organization |
| H. sapiens (human) | HIST1H3F | Chromatin organization |
| H. sapiens (human) | HIST1H3G | Chromatin organization |
| H. sapiens (human) | HIST1H3H | Chromatin organization |
| H. sapiens (human) | HIST1H3I | Chromatin organization |
| H. sapiens (human) | HIST1H3J | Chromatin organization |
| H. sapiens (human) | HIST1H4C | Chromatin organization |
| H. sapiens (human) | HIST1H4D | Chromatin organization |
| H. sapiens (human) | HIST1H4E | Chromatin organization |
| H. sapiens (human) | HIST1H4F | Chromatin organization |
| H. sapiens (human) | HIST1H4I | Chromatin organization |
| H. sapiens (human) | HIST1H4J | Chromatin organization |
| H. sapiens (human) | HIST1H4K | Chromatin organization |
| H. sapiens (human) | HIST1H4L | Chromatin organization |
| H. sapiens (human) | HIST2H4A | Chromatin organization |
| H. sapiens (human) | HIST2H4B | Chromatin organization |
| H. sapiens (human) | ALPL | --- not yet entered --- |
| H. sapiens (human) | CCNB1 | Cell cycle |
| H. sapiens (human) | CTSC | Signaling |
| H. sapiens (human) | COCH | Cell differentiation |
| H. sapiens (human) | DHFR | Metabolism |
| H. sapiens (human) | DSG2 | Cell membrane / wall |
| H. sapiens (human) | GABRB3 | Cell membrane / wall |
| H. sapiens (human) | GLDC | Metabolism |
| H. sapiens (human) | HMMR | Cell membrane / wall |
| H. sapiens (human) | KCNS3 | Cell membrane / wall |
| H. sapiens (human) | LCK | Cell differentiation |
| H. sapiens (human) | PDCD2 | Cell death |
| H. sapiens (human) | RARRES2 | Metabolism |
| H. sapiens (human) | ST6GAL1 | Metabolism |
| H. sapiens (human) | SORL1 | Cell membrane / wall |
| H. sapiens (human) | IFITM1 | Cell membrane / wall |
| H. sapiens (human) | NFE2L3 | RNA transcription and processing |
| H. sapiens (human) | TRIM22 | RNA transcription and processing |
| H. sapiens (human) | LYPLA1 | Metabolism |
| H. sapiens (human) | PIM2 | Cell cycle |
| H. sapiens (human) | RRAS2 | Cell membrane / wall |
| H. sapiens (human) | PASK | Metabolism |
| H. sapiens (human) | FRAT2 | Signaling |
| H. sapiens (human) | CECR1 | Metabolism |
| H. sapiens (human) | NUP107 | Nuclear organization |
| H. sapiens (human) | SLC39A10 | Protein transport |
| H. sapiens (human) | NLGN4X | Cell membrane / wall |
| H. sapiens (human) | LRRN1 | --- unknown --- |
| H. sapiens (human) | ANOS1 | Cell membrane / wall |
| H. sapiens (human) | TMX1 | Metabolism |
| H. sapiens (human) | AKIRIN1 | --- unknown --- |
| H. sapiens (human) | THEMIS2 | Signaling |
| H. sapiens (human) | MIS18BP1 | Cell cycle |
| H. sapiens (human) | ZNF148 | RNA transcription and processing |
| H. sapiens (human) | UBE2D3 | Protein modification |
| H. sapiens (human) | ZNF703 | --- not yet entered --- |
| H. sapiens (human) | ACLY | --- not yet entered --- |
| H. sapiens (human) | ACTN1 | --- not yet entered --- |
| H. sapiens (human) | AP2A2 | --- not yet entered --- |
| H. sapiens (human) | ABCD1 | --- not yet entered --- |
| H. sapiens (human) | ATP2B1 | --- not yet entered --- |
| H. sapiens (human) | ATP2B3 | --- not yet entered --- |
| H. sapiens (human) | DST | --- not yet entered --- |
| H. sapiens (human) | CENPF | --- not yet entered --- |
| H. sapiens (human) | CSNK2A2 | Signaling |
| H. sapiens (human) | CSNK2B | Signaling |
| H. sapiens (human) | CYLC2 | --- not yet entered --- |
| H. sapiens (human) | DMD | --- not yet entered --- |
| H. sapiens (human) | DOCK2 | --- not yet entered --- |
| H. sapiens (human) | ATN1 | --- not yet entered --- |
| H. sapiens (human) | ERH | --- not yet entered --- |
| H. sapiens (human) | FHL1 | --- not yet entered --- |
| H. sapiens (human) | GTF2A1 | --- not yet entered --- |
| H. sapiens (human) | GTF2A2 | --- not yet entered --- |
| H. sapiens (human) | HK1 | --- not yet entered --- |
| H. sapiens (human) | IVD | --- not yet entered --- |
| H. sapiens (human) | KPNA2 | --- not yet entered --- |
| H. sapiens (human) | MARK3 | --- not yet entered --- |
| H. sapiens (human) | MECP2 | --- not yet entered --- |
| H. sapiens (human) | MYH9 | --- not yet entered --- |
| H. sapiens (human) | MYH10 | --- not yet entered --- |
| H. sapiens (human) | MYO1C | --- not yet entered --- |
| H. sapiens (human) | MYO10 | --- not yet entered --- |
| H. sapiens (human) | NME4 | --- not yet entered --- |
| H. sapiens (human) | OPA1 | --- not yet entered --- |
| H. sapiens (human) | PCBP1 | --- not yet entered --- |
| H. sapiens (human) | PCMT1 | --- not yet entered --- |
| H. sapiens (human) | PFKL | --- not yet entered --- |
| H. sapiens (human) | PLAT | --- not yet entered --- |
| H. sapiens (human) | PLOD1 | --- not yet entered --- |
| H. sapiens (human) | PLOD2 | --- not yet entered --- |
| H. sapiens (human) | PPP2CA | --- not yet entered --- |
| H. sapiens (human) | PPP2CB | --- not yet entered --- |
| H. sapiens (human) | PSMA2 | --- not yet entered --- |
| H. sapiens (human) | PSMB5 | --- not yet entered --- |
| H. sapiens (human) | REV3L | --- not yet entered --- |
| H. sapiens (human) | RING1 | --- not yet entered --- |
| H. sapiens (human) | RNF2 | --- not yet entered --- |
| H. sapiens (human) | BRD2 | --- not yet entered --- |
| H. sapiens (human) | RPS2 | --- not yet entered --- |
| H. sapiens (human) | RPS6 | --- not yet entered --- |
| H. sapiens (human) | RPS8 | --- not yet entered --- |
| H. sapiens (human) | RYR2 | --- not yet entered --- |
| H. sapiens (human) | S100A7 | --- not yet entered --- |
| H. sapiens (human) | S100A8 | --- not yet entered --- |
| H. sapiens (human) | SFPQ | --- not yet entered --- |
| H. sapiens (human) | SNRNP70 | --- not yet entered --- |
| H. sapiens (human) | SNRPD1 | --- not yet entered --- |
| H. sapiens (human) | TAF2 | --- not yet entered --- |
| H. sapiens (human) | TBX1 | --- not yet entered --- |
| H. sapiens (human) | TF | --- not yet entered --- |
| H. sapiens (human) | TRPC5 | --- not yet entered --- |
| H. sapiens (human) | UGDH | --- not yet entered --- |
| H. sapiens (human) | UTRN | --- not yet entered --- |
| H. sapiens (human) | SF1 | --- not yet entered --- |
| H. sapiens (human) | USP9X | --- not yet entered --- |
| H. sapiens (human) | DUSP11 | --- not yet entered --- |
| H. sapiens (human) | IRS4 | --- not yet entered --- |
| H. sapiens (human) | DDX3Y | --- not yet entered --- |
| H. sapiens (human) | PABPC4 | --- not yet entered --- |
| H. sapiens (human) | MCM3AP | --- not yet entered --- |
| H. sapiens (human) | MYOM2 | --- not yet entered --- |
| H. sapiens (human) | SRSF11 | --- not yet entered --- |
| H. sapiens (human) | FXR2 | --- not yet entered --- |
| H. sapiens (human) | TBPL1 | --- not yet entered --- |
| H. sapiens (human) | KIAA0430 | --- not yet entered --- |
| H. sapiens (human) | G3BP2 | --- not yet entered --- |
| H. sapiens (human) | SEC16A | --- not yet entered --- |
| H. sapiens (human) | USP15 | --- not yet entered --- |
| H. sapiens (human) | THRAP3 | --- not yet entered --- |
| H. sapiens (human) | AASS | --- not yet entered --- |
| H. sapiens (human) | SF3B4 | --- not yet entered --- |
| H. sapiens (human) | BCKDK | --- not yet entered --- |
| H. sapiens (human) | PRMT5 | --- not yet entered --- |
| H. sapiens (human) | SEC24B | --- not yet entered --- |
| H. sapiens (human) | PITRM1 | --- not yet entered --- |
| H. sapiens (human) | PRDX4 | --- not yet entered --- |
| H. sapiens (human) | PRDX3 | --- not yet entered --- |
| H. sapiens (human) | CLASRP | --- not yet entered --- |
| H. sapiens (human) | ATXN2L | --- not yet entered --- |
| H. sapiens (human) | CEP131 | --- not yet entered --- |
| H. sapiens (human) | FBXO21 | --- not yet entered --- |
| H. sapiens (human) | ZC3H13 | --- not yet entered --- |
| H. sapiens (human) | GPATCH8 | --- not yet entered --- |
| H. sapiens (human) | CYFIP1 | --- not yet entered --- |
| H. sapiens (human) | GANAB | --- not yet entered --- |
| H. sapiens (human) | SYNE2 | --- not yet entered --- |
| H. sapiens (human) | KIF13B | --- not yet entered --- |
| H. sapiens (human) | SYNE1 | --- not yet entered --- |
| H. sapiens (human) | ARHGEF18 | --- not yet entered --- |
| H. sapiens (human) | MACF1 | --- not yet entered --- |
| H. sapiens (human) | SKIV2L2 | --- not yet entered --- |
| H. sapiens (human) | SRRM2 | --- not yet entered --- |
| H. sapiens (human) | ZNF281 | --- not yet entered --- |
| H. sapiens (human) | SDF2L1 | --- not yet entered --- |
| H. sapiens (human) | KIAA1429 | --- not yet entered --- |
| H. sapiens (human) | CHD5 | --- not yet entered --- |
| H. sapiens (human) | SAP30BP | --- not yet entered --- |
| H. sapiens (human) | REPIN1 | --- not yet entered --- |
| H. sapiens (human) | RBM15B | --- not yet entered --- |
| H. sapiens (human) | YTHDF2 | --- not yet entered --- |
| H. sapiens (human) | LARP7 | --- not yet entered --- |
| H. sapiens (human) | YTHDF1 | --- not yet entered --- |
| H. sapiens (human) | C20orf27 | --- not yet entered --- |
| H. sapiens (human) | DARS2 | --- not yet entered --- |
| H. sapiens (human) | CHD7 | --- not yet entered --- |
| H. sapiens (human) | BAIAP2L1 | --- not yet entered --- |
| H. sapiens (human) | ARHGAP20 | --- not yet entered --- |
| H. sapiens (human) | DHX37 | --- not yet entered --- |
| H. sapiens (human) | CHD8 | --- not yet entered --- |
| H. sapiens (human) | EEFSEC | --- not yet entered --- |
| H. sapiens (human) | MCCC2 | --- not yet entered --- |
| H. sapiens (human) | TBC1D15 | --- not yet entered --- |
| H. sapiens (human) | NT5DC2 | --- not yet entered --- |
| H. sapiens (human) | PYCRL | --- not yet entered --- |
| H. sapiens (human) | KCTD15 | RNA transcription and processing |
| H. sapiens (human) | DHX40 | --- not yet entered --- |
| H. sapiens (human) | SLTM | --- not yet entered --- |
| H. sapiens (human) | SNX22 | Signaling |
| H. sapiens (human) | EDC3 | --- not yet entered --- |
| H. sapiens (human) | GRPEL1 | --- not yet entered --- |
| H. sapiens (human) | TMPRSS13 | --- not yet entered --- |
| H. sapiens (human) | LDOC1L | --- not yet entered --- |
| H. sapiens (human) | POLDIP3 | --- not yet entered --- |
| H. sapiens (human) | ZGPAT | --- not yet entered --- |
| H. sapiens (human) | TRIM47 | --- not yet entered --- |
| H. sapiens (human) | ZC3HAV1L | --- not yet entered --- |
| H. sapiens (human) | ARMC6 | --- not yet entered --- |
| H. sapiens (human) | PAXBP1 | --- not yet entered --- |
| H. sapiens (human) | OSBPL9 | --- not yet entered --- |
| H. sapiens (human) | OSBPL11 | --- not yet entered --- |
| H. sapiens (human) | RAVER1 | --- not yet entered --- |
| H. sapiens (human) | MACROD2 | --- not yet entered --- |
| H. sapiens (human) | RBM33 | --- not yet entered --- |
| H. sapiens (human) | CFAP58 | --- not yet entered --- |
| H. sapiens (human) | U2AF1L4 | --- not yet entered --- |
| H. sapiens (human) | PRPS1L1 | --- not yet entered --- |
| H. sapiens (human) | RSBN1L | --- not yet entered --- |
| H. sapiens (human) | YTHDF3 | --- not yet entered --- |
| H. sapiens (human) | RALGAPA1 | --- not yet entered --- |
| H. sapiens (human) | RASGEF1C | --- not yet entered --- |
| H. sapiens (human) | GCOM2 | --- not yet entered --- |
| H. sapiens (human) | KIAA2022 | --- not yet entered --- |
| H. sapiens (human) | ZKSCAN4 | --- not yet entered --- |
| H. sapiens (human) | HSP90AB2P | --- not yet entered --- |
| H. sapiens (human) | TMEM173 | Signaling |
| H. sapiens (human) | CEBPB | RNA transcription and processing |
| H. sapiens (human) | SETDB1 | Chromatin organization |
| H. sapiens (human) | EP300 | --- not yet entered --- |
| H. sapiens (human) | HDAC9 | --- not yet entered --- |
| H. sapiens (human) | USP22 | --- not yet entered --- |
| H. sapiens (human) | BMP7 | Signaling |
| H. sapiens (human) | CACNA1B | --- not yet entered --- |
| H. sapiens (human) | CYP2E1 | --- not yet entered --- |
| H. sapiens (human) | FOXD4 | --- not yet entered --- |
| H. sapiens (human) | FRG1 | --- not yet entered --- |
| H. sapiens (human) | GAS8 | --- not yet entered --- |
| H. sapiens (human) | PCNT | --- not yet entered --- |
| H. sapiens (human) | PSMD13 | --- not yet entered --- |
| H. sapiens (human) | SDHA | --- not yet entered --- |
| H. sapiens (human) | SLC6A12 | --- not yet entered --- |
| H. sapiens (human) | ZNF140 | --- not yet entered --- |
| H. sapiens (human) | DOC2B | --- not yet entered --- |
| H. sapiens (human) | UBE2M | --- not yet entered --- |
| H. sapiens (human) | ZMYND11 | --- not yet entered --- |
| H. sapiens (human) | TMED10 | --- not yet entered --- |
| H. sapiens (human) | DIP2C | --- not yet entered --- |
| H. sapiens (human) | SIRT3 | --- not yet entered --- |
| H. sapiens (human) | SH3YL1 | --- not yet entered --- |
| H. sapiens (human) | DUSP22 | --- not yet entered --- |
| H. sapiens (human) | RIC8A | --- not yet entered --- |
| H. sapiens (human) | DBNDD1 | --- not yet entered --- |
| H. sapiens (human) | RBFA | --- not yet entered --- |
| H. sapiens (human) | TRIM7 | --- not yet entered --- |
| H. sapiens (human) | SCGB1C1 | --- not yet entered --- |
| H. sapiens (human) | CHAMP1 | --- not yet entered --- |
| H. sapiens (human) | IQSEC3 | --- not yet entered --- |
|  | CTAG1A | --- not yet entered --- |
|  | CTAG1B | --- not yet entered --- |
|  | CTAG2 | --- not yet entered --- |
|  | LAGE3 | --- not yet entered --- |

Supplementary Table 2 | Results of 471 telomere-related DEGs.

| id | logFC | AveExpr | t | P.Value | adj.P.Val | B |
| --- | --- | --- | --- | --- | --- | --- |
| ABCB6 | 0.583061664 | 8.528354394 | 7.980521566 | 1.48E-11 | 7.94E-10 | 16.03050142 |
| ABCC5 | 0.941100538 | 9.212637056 | 8.197255789 | 5.77E-12 | 3.57E-10 | 16.95172596 |
| ABCC6 | -0.605414034 | 8.249335803 | -6.227076747 | 2.67E-08 | 4.27E-07 | 8.699946982 |
| ACTR2 | 0.74361804 | 10.29630132 | 5.159632368 | 2.03E-06 | 1.56E-05 | 4.497378307 |
| ACTR3 | 0.721905757 | 11.63403406 | 5.654748198 | 2.82E-07 | 3.02E-06 | 6.409203409 |
| ADPRH | -0.572245442 | 6.374296958 | -4.617674215 | 1.62E-05 | 8.69E-05 | 2.500344065 |
| ADSS | 0.632080137 | 9.220333479 | 4.436979941 | 3.15E-05 | 0.000150072 | 1.860792016 |
| AHSA1 | 0.577291106 | 10.18339627 | 7.102531184 | 6.55E-10 | 1.98E-08 | 12.32043605 |
| AIFM1 | 0.560404736 | 9.460644831 | 6.373367593 | 1.45E-08 | 2.58E-07 | 9.296617238 |
| AK1 | -0.754605126 | 10.15580176 | -6.584750554 | 5.95E-09 | 1.26E-07 | 10.16538793 |
| AKR1B10 | 1.27240554 | 4.068317831 | 6.112122889 | 4.31E-08 | 6.33E-07 | 8.233990895 |
| AKT1 | -0.791329661 | 8.99189707 | -5.124555843 | 2.33E-06 | 1.75E-05 | 4.364866419 |
| ALDH1A1 | 0.553563715 | 11.31412255 | 3.443701587 | 0.000951394 | 0.002551481 | -1.36551277 |
| ALDH3A1 | 1.107453276 | 7.067546352 | 4.578486852 | 1.87E-05 | 9.77E-05 | 2.360442315 |
| ALPL | -0.657998289 | 8.871195831 | -3.593308315 | 0.000587634 | 0.001702216 | -0.914534472 |
| AMPD1 | 1.214308455 | 7.399807042 | 5.552857842 | 4.26E-07 | 4.27E-06 | 6.009860239 |
| ANAPC1 | 0.504307985 | 8.577655014 | 5.51760189 | 4.90E-07 | 4.78E-06 | 5.872352473 |
| ANAPC4 | 0.646453421 | 10.04794742 | 3.849447148 | 0.000250575 | 0.000838404 | -0.111892066 |
| ANGPT4 | -0.537904542 | 5.260243817 | -4.880459804 | 5.99E-06 | 3.86E-05 | 3.454758803 |
| ANP32E | 0.597487458 | 7.881822465 | 3.778401989 | 0.000318472 | 0.001019851 | -0.33825765 |
| APAF1 | 0.577416091 | 7.769574676 | 4.007091057 | 0.00014589 | 0.000537401 | 0.400214721 |
| ARF5 | -0.554029639 | 10.12300592 | -4.138373743 | 9.22E-05 | 0.000367411 | 0.83662928 |
| ARHGAP10 | 0.539019286 | 8.630794211 | 4.560378882 | 2.00E-05 | 0.000103208 | 2.296016863 |
| ARHGAP12 | 0.552839481 | 10.05557403 | 3.359597919 | 0.00124063 | 0.003198607 | -1.612986214 |
| ARHGAP15 | 0.581950706 | 8.074454535 | 3.896295049 | 0.000213636 | 0.000735274 | 0.038897323 |
| ARHGAP27 | -0.59043936 | 7.976524986 | -5.330188435 | 1.04E-06 | 8.93E-06 | 5.147506415 |
| ARID4A | 0.658267629 | 7.629581493 | 3.184990127 | 0.002125102 | 0.005026378 | -2.112331794 |
| ARL4A | 0.508097244 | 7.433059465 | 4.420735122 | 3.34E-05 | 0.000157715 | 1.804001666 |
| ARL4D | -0.547542355 | 8.04307269 | -3.102524587 | 0.002723148 | 0.006248677 | -2.341187148 |
| ARRB1 | -1.169487384 | 9.419246563 | -7.307806171 | 2.71E-10 | 9.39E-09 | 13.18307521 |
| ARRDC1 | -0.599771221 | 9.008184465 | -4.701542438 | 1.18E-05 | 6.70E-05 | 2.80192226 |
| ARRDC2 | -0.857979425 | 10.08379649 | -6.608871223 | 5.37E-09 | 1.16E-07 | 10.2649784 |
| ARRDC4 | 0.589439739 | 8.995532606 | 3.651741596 | 0.000485241 | 0.001453134 | -0.734751497 |
| ASPG | -1.293687521 | 7.535113859 | -7.261833571 | 3.30E-10 | 1.12E-08 | 12.98955285 |
| ATAD2 | 0.955359426 | 6.817891803 | 5.53557575 | 4.56E-07 | 4.50E-06 | 5.94241135 |
| ATF7 | -0.533065119 | 7.605578634 | -6.279811078 | 2.14E-08 | 3.56E-07 | 8.914571943 |
| ATM | 0.77574634 | 8.236573831 | 4.124059024 | 9.69E-05 | 0.000382907 | 0.788617915 |
| ATN1 | -0.560553394 | 9.222682718 | -6.061044658 | 5.33E-08 | 7.54E-07 | 8.027821749 |
| ATP2A2 | 0.772728757 | 9.957313817 | 7.978404825 | 1.49E-11 | 7.98E-10 | 16.02150962 |
| ATP5A1 | 0.583182883 | 11.92023892 | 6.321372012 | 1.80E-08 | 3.07E-07 | 9.084091596 |
| ATP6V1A | 0.621975769 | 10.49527723 | 5.418657047 | 7.29E-07 | 6.67E-06 | 5.488359646 |
| ATR | 0.896600013 | 8.865262268 | 5.371635835 | 8.79E-07 | 7.84E-06 | 5.306897045 |
| ATRX | 0.782121638 | 8.293707282 | 4.41934746 | 3.36E-05 | 0.000158122 | 1.799156068 |
| BAZ2A | -0.625572938 | 9.388063648 | -8.643807298 | 8.30E-13 | 7.23E-11 | 18.85101165 |
| BCL7B | -0.637679878 | 8.800157197 | -5.950668269 | 8.41E-08 | 1.09E-06 | 7.584239063 |
| BDKRB2 | 0.694770049 | 9.260006099 | 3.767377223 | 0.00033047 | 0.00105094 | -0.373132383 |
| BHLHE40 | -0.595827321 | 10.87179104 | -4.080713803 | 0.000112864 | 0.000434884 | 0.643869151 |
| BLM | 0.532423348 | 6.728396225 | 5.188881703 | 1.81E-06 | 1.42E-05 | 4.608194086 |
| BRCA1 | 0.673094574 | 6.55938793 | 5.827423972 | 1.40E-07 | 1.68E-06 | 7.092239336 |
| BRMS1L | 0.734759549 | 7.208013056 | 3.896520801 | 0.000213471 | 0.000735041 | 0.039626842 |
| BUB3 | 0.595111962 | 9.728592676 | 6.847849645 | 1.95E-09 | 4.92E-08 | 11.2561923 |
| BZW1 | 0.858082595 | 10.7899927 | 5.12081914 | 2.36E-06 | 1.77E-05 | 4.350774669 |
| C19orf66 | -0.512715415 | 9.747214141 | -7.081736026 | 7.16E-10 | 2.12E-08 | 12.23327151 |
| CACYBP | 0.579073488 | 8.968687831 | 5.535658361 | 4.56E-07 | 4.50E-06 | 5.942733565 |
| CALD1 | 0.947973975 | 10.86549785 | 5.437289922 | 6.77E-07 | 6.25E-06 | 5.560451266 |
| CAMK1 | -0.589289962 | 8.053091324 | -3.798028366 | 0.000298136 | 0.000966222 | -0.276004533 |
| CAMK1D | 0.583985846 | 9.270963028 | 5.302752682 | 1.16E-06 | 9.76E-06 | 5.042293887 |
| CAMK2A | -0.796025744 | 5.071890141 | -5.7439911 | 1.96E-07 | 2.21E-06 | 6.761261076 |
| CAND1 | 0.759888576 | 8.597474366 | 5.370560978 | 8.83E-07 | 7.86E-06 | 5.302756863 |
| CAPRIN1 | 0.568787482 | 10.48383349 | 6.269611258 | 2.24E-08 | 3.69E-07 | 8.873018046 |
| CARS | 0.559250967 | 7.849105338 | 5.114899048 | 2.42E-06 | 1.81E-05 | 4.328458796 |
| CBX3 | 0.578952411 | 10.87036217 | 4.113055858 | 0.00010075 | 0.000395044 | 0.751783473 |
| CCDC155 | -0.761126267 | 5.587542028 | -3.564650197 | 0.000645057 | 0.001840399 | -1.001968827 |
| CCNA2 | 0.591886687 | 5.991043296 | 6.430816586 | 1.14E-08 | 2.13E-07 | 9.531989837 |
| CCNB1 | 1.128704581 | 7.248487451 | 11.01690784 | 3.23E-17 | 1.63E-14 | 28.79587242 |
| CCT2 | 0.584385119 | 11.41440913 | 4.899340278 | 5.57E-06 | 3.63E-05 | 3.524375173 |
| CCT4 | 0.531040177 | 11.81351018 | 5.695146261 | 2.39E-07 | 2.62E-06 | 6.568313323 |
| CCT5 | 0.624933426 | 10.25057787 | 4.736779651 | 1.03E-05 | 6.03E-05 | 2.929491973 |
| CCT6A | 0.797848175 | 10.46902569 | 5.082759667 | 2.74E-06 | 1.99E-05 | 4.20752026 |
| CCT8 | 0.616215412 | 9.930569141 | 4.516707952 | 2.35E-05 | 0.000118483 | 2.141225077 |
| CDK2 | -0.637036309 | 7.581884746 | -5.618485667 | 3.27E-07 | 3.43E-06 | 6.2667527 |
| CDKL2 | -0.622648485 | 7.748735521 | -3.611145091 | 0.000554381 | 0.001623906 | -0.859869018 |
| CDKN1A | -0.517710303 | 10.54545152 | -2.635499086 | 0.010243744 | 0.019346881 | -3.547517215 |
| CDKN2B | -0.718007512 | 7.755375239 | -5.263663259 | 1.35E-06 | 1.11E-05 | 4.892803378 |
| CEBPA | -0.686822511 | 9.626919958 | -3.800556814 | 0.000295608 | 0.000959 | -0.267968854 |
| CEBPB | -0.575619254 | 12.37714455 | -5.857009051 | 1.24E-07 | 1.52E-06 | 7.210015702 |
| CEBPB | -0.575619254 | 12.37714455 | -5.857009051 | 1.24E-07 | 1.52E-06 | 7.210015702 |
| CENPF | 1.037518937 | 7.000061732 | 7.881574899 | 2.27E-11 | 1.14E-09 | 15.61032836 |
| CHD8 | 0.530600999 | 8.960319831 | 5.224512654 | 1.57E-06 | 1.26E-05 | 4.743572418 |
| CHEK1 | 1.074961683 | 6.217604704 | 8.637702748 | 8.53E-13 | 7.34E-11 | 18.82505514 |
| CHEK2 | 0.616999356 | 6.734677507 | 7.916677482 | 1.95E-11 | 9.97E-10 | 15.75935401 |
| CHMP2B | 0.624592549 | 8.644007901 | 4.179765815 | 7.96E-05 | 0.00032584 | 0.976032987 |
| CHN1 | 0.656763625 | 10.46444637 | 4.510151979 | 2.41E-05 | 0.000120741 | 2.11805915 |
| CKB | -0.577354029 | 10.74559177 | -4.022475389 | 0.0001383 | 0.000513944 | 0.450897247 |
| CLIC3 | -1.382298156 | 9.082166958 | -7.059004009 | 7.89E-10 | 2.31E-08 | 12.1380395 |
| COCH | 0.654819364 | 5.929088127 | 5.735638589 | 2.03E-07 | 2.28E-06 | 6.728223242 |
| CPNE2 | -0.716465649 | 10.52251817 | -6.167701588 | 3.42E-08 | 5.24E-07 | 8.458942313 |
| CPSF4 | -0.585184534 | 9.265831817 | -7.037332842 | 8.66E-10 | 2.49E-08 | 12.04730237 |
| CREB5 | -1.098605122 | 7.874707479 | -7.634018453 | 6.63E-11 | 2.79E-09 | 14.56077381 |
| CRYM | 0.554999831 | 9.456807225 | 3.234274768 | 0.001828875 | 0.004435578 | -1.97339757 |
| CSNK1A1 | 0.661183697 | 10.97701086 | 4.102665363 | 0.000104498 | 0.000407212 | 0.717056121 |
| CSNK1G2 | -0.744805551 | 9.637971028 | -6.269034218 | 2.24E-08 | 3.70E-07 | 8.870667784 |
| CSTF3 | 0.644676958 | 8.552992803 | 5.437708589 | 6.76E-07 | 6.25E-06 | 5.5620723 |
| CTNNB1 | 0.697782926 | 11.98835317 | 4.183048758 | 7.87E-05 | 0.000322761 | 0.987125908 |
| DARS | 0.608291932 | 11.45620356 | 4.744755226 | 1.00E-05 | 5.90E-05 | 2.958435647 |
| DARS2 | 0.68212451 | 7.253975859 | 7.12634003 | 5.91E-10 | 1.81E-08 | 12.42028632 |
| DDX1 | 0.787351213 | 11.53804107 | 4.688172569 | 1.24E-05 | 7.02E-05 | 2.753651372 |
| DDX21 | 0.664047394 | 10.18855307 | 2.997811613 | 0.003708925 | 0.008105481 | -2.625127516 |
| DDX3X | 0.629674303 | 10.82829815 | 3.425430764 | 0.001008209 | 0.002674491 | -1.419650385 |
| DDX3Y | 2.473068394 | 8.359400676 | 4.1677266 | 8.31E-05 | 0.000337765 | 0.935398572 |
| DDX46 | 0.633164009 | 8.701057479 | 4.158291711 | 8.59E-05 | 0.000347172 | 0.903604389 |
| DDX5 | 0.532402662 | 13.37624042 | 3.613576043 | 0.00054999 | 0.001614165 | -0.85240416 |
| DDX50 | 0.750105497 | 9.894755746 | 5.235020623 | 1.51E-06 | 1.22E-05 | 4.783576817 |
| DEK | 0.867950056 | 10.13773069 | 4.463553651 | 2.86E-05 | 0.000138502 | 1.953947344 |
| DHX15 | 0.626030697 | 10.90866321 | 3.887315723 | 0.000220286 | 0.000753197 | 0.009902934 |
| DHX32 | 0.555260746 | 8.459372479 | 6.355802723 | 1.56E-08 | 2.72E-07 | 9.22476853 |
| DHX36 | 0.659545894 | 9.621116507 | 3.959454085 | 0.000172012 | 0.000614788 | 0.244065682 |
| DHX40 | 0.792358012 | 6.194830817 | 5.30498902 | 1.15E-06 | 9.69E-06 | 5.05086107 |
| DHX9 | 0.564312153 | 10.3595879 | 4.38763238 | 3.77E-05 | 0.000173771 | 1.688648756 |
| DLG2 | 0.859463311 | 6.437704268 | 6.835819905 | 2.05E-09 | 5.15E-08 | 11.20611259 |
| DMD | 0.613920645 | 7.748189211 | 4.555507667 | 2.04E-05 | 0.000104738 | 2.278709872 |
| DNAJA4 | 1.213356921 | 9.488218958 | 5.731603613 | 2.06E-07 | 2.31E-06 | 6.712269546 |
| DNAJC3 | 0.835551438 | 8.950440817 | 5.796430272 | 1.58E-07 | 1.86E-06 | 6.969084723 |
| DOC2B | -0.576223031 | 5.498136451 | -3.283684965 | 0.001571098 | 0.003899939 | -1.832511394 |
| DOK2 | -0.606982019 | 8.618253394 | -3.73141418 | 0.00037268 | 0.001163674 | -0.486417086 |
| DOT1L | -1.026286291 | 8.060683704 | -9.895876972 | 3.74E-15 | 8.32E-13 | 24.14640953 |
| DQX1 | 0.63710193 | 5.73620869 | 4.263041961 | 5.91E-05 | 0.000254548 | 1.259047172 |
| DSG2 | 0.705746109 | 7.697427141 | 5.05291605 | 3.08E-06 | 2.20E-05 | 4.095543256 |
| DST | 0.670578765 | 9.743069056 | 3.416348847 | 0.001037628 | 0.002742722 | -1.446483421 |
| DYDC1 | 0.875527673 | 3.985558944 | 3.78350885 | 0.000313056 | 0.001005911 | -0.322079918 |
| DYDC2 | 1.224354274 | 6.48545638 | 3.19832232 | 0.00204082 | 0.004861188 | -2.074906359 |
| E2F3 | 0.560420785 | 8.590927887 | 4.103949497 | 0.000104028 | 0.000405692 | 0.721345028 |
| ECT2 | 0.787328783 | 7.584658662 | 5.027077921 | 3.40E-06 | 2.39E-05 | 3.998849513 |
| EFCAB7 | 0.697143025 | 7.871851944 | 4.01376157 | 0.000142551 | 0.000526897 | 0.422175071 |
| EIF2AK4 | 0.676501039 | 9.186031803 | 5.539165466 | 4.50E-07 | 4.46E-06 | 5.956414422 |
| EIF4A2 | 0.768968984 | 10.97144675 | 4.656401613 | 1.40E-05 | 7.72E-05 | 2.639239605 |
| EIF5B | 0.88032482 | 8.56110262 | 4.819731301 | 7.55E-06 | 4.68E-05 | 3.231763395 |
| ELOVL1 | -0.779291892 | 9.911877296 | -5.482919478 | 5.64E-07 | 5.35E-06 | 5.737428564 |
| EMD | -0.851962223 | 9.322939366 | -5.741000381 | 1.99E-07 | 2.24E-06 | 6.749429425 |
| EPAS1 | -0.706266275 | 13.40593768 | -7.243914665 | 3.57E-10 | 1.19E-08 | 12.91417192 |
| EPHA4 | 0.612627709 | 8.641699014 | 4.869127425 | 6.25E-06 | 4.00E-05 | 3.413038858 |
| ERCC4 | 0.684197511 | 6.31817031 | 5.722335464 | 2.14E-07 | 2.39E-06 | 6.675640521 |
| ERH | -0.69845675 | 11.26884061 | -4.94820636 | 4.62E-06 | 3.09E-05 | 3.705176437 |
| ESR2 | 0.991251317 | 5.740811915 | 6.15192199 | 3.66E-08 | 5.52E-07 | 8.395011153 |
| ETAA1 | 0.714841038 | 7.696928648 | 4.495933986 | 2.54E-05 | 0.000125778 | 2.06788389 |
| ETHE1 | -0.683204197 | 9.543092986 | -5.210779902 | 1.66E-06 | 1.32E-05 | 4.691345713 |
| ETS2 | -0.557498868 | 12.24823607 | -6.406206409 | 1.26E-08 | 2.31E-07 | 9.431089933 |
| ETV1 | -0.595649526 | 8.551532845 | -3.372980624 | 0.001189635 | 0.003084986 | -1.573905192 |
| EVL | -0.502771292 | 10.11779083 | -5.549744954 | 4.31E-07 | 4.32E-06 | 5.997704978 |
| EWSR1 | 0.537346544 | 9.762016254 | 4.143205068 | 9.06E-05 | 0.000361864 | 0.85285665 |
| EXO1 | 0.908281632 | 5.358457803 | 8.307488267 | 3.58E-12 | 2.40E-10 | 17.42054809 |
| EXOSC6 | -0.523395059 | 9.31787093 | -6.07150753 | 5.11E-08 | 7.30E-07 | 8.070008412 |
| FANCI | 0.708792885 | 7.018633042 | 6.956577514 | 1.22E-09 | 3.35E-08 | 11.70963161 |
| FANCM | 0.703906091 | 6.613942394 | 4.164330656 | 8.41E-05 | 0.000341303 | 0.923949652 |
| FES | -0.50455814 | 9.015625014 | -4.470832938 | 2.78E-05 | 0.000135588 | 1.97952035 |
| FGFR3 | -0.620900181 | 9.019470408 | -4.151535825 | 8.80E-05 | 0.000354074 | 0.880865233 |
| FGFR4 | -1.077247571 | 8.06380107 | -6.407996215 | 1.25E-08 | 2.30E-07 | 9.438424484 |
| FHL1 | -0.604482617 | 10.86521268 | -4.901587038 | 5.52E-06 | 3.60E-05 | 3.532668432 |
| FKBP3 | 0.613894824 | 9.564076423 | 4.516530586 | 2.35E-05 | 0.000118522 | 2.140598093 |
| FKBP8 | -0.585123352 | 8.286933479 | -4.592432771 | 1.77E-05 | 9.37E-05 | 2.410155476 |
| FOSL1 | -0.925204678 | 6.543901169 | -3.968824891 | 0.000166543 | 0.00059877 | 0.274687615 |
| FOXK2 | -0.504661132 | 9.56069393 | -5.241515685 | 1.47E-06 | 1.20E-05 | 4.808321896 |
| FOXP4 | -0.586794269 | 7.011281408 | -4.991862281 | 3.90E-06 | 2.68E-05 | 3.867447184 |
| FOXR1 | -0.665460201 | 4.248155873 | -4.259043739 | 6.00E-05 | 0.000257354 | 1.245382426 |
| FUBP1 | 0.602575601 | 9.62476569 | 6.041195058 | 5.79E-08 | 8.04E-07 | 7.947852152 |
| G3BP1 | 0.516673544 | 10.79891765 | 4.542757615 | 2.13E-05 | 0.000109024 | 2.233458601 |
| GAP43 | 0.909067651 | 4.805703254 | 5.526420089 | 4.73E-07 | 4.64E-06 | 5.906712732 |
| GARS | 0.556391604 | 8.807701451 | 6.086324256 | 4.80E-08 | 6.92E-07 | 8.129789872 |
| GATA1 | -0.518622697 | 5.672420282 | -3.315199799 | 0.00142495 | 0.003592495 | -1.741825053 |
| GATA2 | -1.210804688 | 8.087083155 | -7.518945114 | 1.09E-10 | 4.30E-09 | 14.07397046 |
| GATA3 | -0.861656332 | 6.398269366 | -5.359505194 | 9.23E-07 | 8.13E-06 | 5.260192321 |
| GATA6 | -0.844219879 | 10.30958541 | -8.158353658 | 6.83E-12 | 4.11E-10 | 16.78630758 |
| GATAD2B | -0.544824248 | 7.770776549 | -4.984325068 | 4.02E-06 | 2.74E-05 | 3.839381332 |
| GBE1 | 0.728415657 | 10.06380355 | 4.771625877 | 9.06E-06 | 5.42E-05 | 3.05613765 |
| GEN1 | 0.831683431 | 5.22439707 | 4.581266522 | 1.85E-05 | 9.69E-05 | 2.370344411 |
| GLG1 | 0.571793538 | 11.54226282 | 8.397070772 | 2.42E-12 | 1.74E-10 | 17.80160211 |
| GLI1 | 0.615825352 | 7.278758535 | 3.488396657 | 0.000824899 | 0.002263542 | -1.232208935 |
| GMNN | 0.728577847 | 8.444453423 | 5.745624454 | 1.95E-07 | 2.21E-06 | 6.767723789 |
| GNL3 | 0.71847238 | 9.918485549 | 4.383156037 | 3.83E-05 | 0.000175981 | 1.67308854 |
| GPA33 | -1.105343681 | 7.186076254 | -3.641758888 | 0.000501439 | 0.001496611 | -0.765607598 |
| GREM1 | 2.197131712 | 6.450238845 | 7.053783481 | 8.07E-10 | 2.35E-08 | 12.11617661 |
| GTF2A2 | -0.592864387 | 8.816190535 | -5.245395846 | 1.45E-06 | 1.18E-05 | 4.823111205 |
| GTF3C3 | 0.768919588 | 8.876601324 | 5.208310125 | 1.68E-06 | 1.33E-05 | 4.681959555 |
| H2AFY2 | 0.518106074 | 7.719815282 | 6.358630225 | 1.54E-08 | 2.70E-07 | 9.236330643 |
| HAGH | -0.564790701 | 10.23685399 | -5.022255822 | 3.47E-06 | 2.42E-05 | 3.980830171 |
| HCLS1 | 0.631011462 | 11.61950596 | 5.518898184 | 4.88E-07 | 4.76E-06 | 5.877402109 |
| HDAC2 | 0.586298609 | 10.14117948 | 4.479174617 | 2.70E-05 | 0.000132262 | 2.00885467 |
| HDAC7 | -0.552142931 | 10.72312799 | -6.626873608 | 4.98E-09 | 1.09E-07 | 10.3393648 |
| HIC1 | -0.727601353 | 7.884620944 | -4.466063899 | 2.83E-05 | 0.000137673 | 1.962763477 |
| HIF1A | 0.859486032 | 10.48576501 | 4.459179589 | 2.90E-05 | 0.000140383 | 1.938592116 |
| HINT2 | -0.983607749 | 9.601561254 | -6.460650354 | 1.00E-08 | 1.93E-07 | 9.654444594 |
| HIP1 | -0.681153444 | 7.985796028 | -5.642198473 | 2.97E-07 | 3.16E-06 | 6.359864024 |
| HIST1H3A | -0.632253137 | 9.095848873 | -3.941035904 | 0.00018327 | 0.000648445 | 0.184014696 |
| HIST1H3B | -0.771306314 | 8.288379155 | -4.239384788 | 6.44E-05 | 0.000272563 | 1.178305799 |
| HIST1H3F | -0.522659222 | 6.247220113 | -3.806124291 | 0.000290114 | 0.000945225 | -0.250262242 |
| HIST1H3H | -0.554871103 | 6.156274324 | -3.892706758 | 0.00021627 | 0.000742315 | 0.027305427 |
| HIST1H3I | -1.091087168 | 7.984028225 | -4.632908196 | 1.53E-05 | 8.30E-05 | 2.554905727 |
| HIST1H4C | -0.98093373 | 10.90650075 | -4.211762826 | 7.10E-05 | 0.000296898 | 1.084375302 |
| HIST1H4D | -0.85831794 | 6.215217056 | -4.103573042 | 0.000104165 | 0.000406124 | 0.720087611 |
| HIST1H4E | -0.988462862 | 6.980459338 | -3.595333907 | 0.000583764 | 0.001693598 | -0.908336005 |
| HIST1H4L | -0.903980021 | 8.133683493 | -4.330236743 | 4.64E-05 | 0.000207328 | 1.489837908 |
| HIST2H4B | -0.5254708 | 8.229034127 | -2.536647535 | 0.013316583 | 0.024331277 | -3.782286834 |
| HLTF | 0.755782306 | 8.435418493 | 5.559547426 | 4.14E-07 | 4.18E-06 | 6.035991061 |
| HMGB3 | 1.243589826 | 8.983296042 | 7.475917381 | 1.31E-10 | 5.03E-09 | 13.89215828 |
| HMMR | 0.819542736 | 5.835230225 | 5.27997317 | 1.26E-06 | 1.05E-05 | 4.955118355 |
| HMOX1 | -1.032830553 | 11.01878182 | -4.843748335 | 6.89E-06 | 4.34E-05 | 3.319784356 |
| HNMT | 0.530473811 | 8.629277296 | 4.758589103 | 9.52E-06 | 5.64E-05 | 3.008699799 |
| HNRNPA1 | 0.53104726 | 10.24361463 | 3.855492818 | 0.000245485 | 0.000825202 | -0.092500277 |
| HNRNPA2B1 | 0.692448632 | 11.50600782 | 4.40591651 | 3.53E-05 | 0.000164226 | 1.752301555 |
| HNRNPM | 0.576468988 | 11.80526859 | 4.532752172 | 2.21E-05 | 0.00011249 | 2.197997569 |
| HNRNPU | 0.531195686 | 11.09189659 | 3.990310494 | 0.000154624 | 0.000564525 | 0.345073506 |
| HSP90AA1 | 1.19435581 | 12.14022 | 6.025200226 | 6.18E-08 | 8.45E-07 | 7.883474726 |
| HSP90AB1 | 0.528102899 | 11.51682503 | 5.361318188 | 9.16E-07 | 8.10E-06 | 5.267169732 |
| HSP90B1 | 0.729222679 | 13.21149704 | 5.187334052 | 1.82E-06 | 1.43E-05 | 4.602323384 |
| HSPA4 | 0.627102828 | 10.40390758 | 4.806371435 | 7.94E-06 | 4.87E-05 | 3.182897386 |
| HSPA4L | 1.899417385 | 6.046099704 | 9.129557089 | 1.01E-13 | 1.26E-11 | 20.91361448 |
| HSPH1 | 1.208625257 | 10.78804275 | 5.061995536 | 2.97E-06 | 2.13E-05 | 4.129577492 |
| IDH1 | 0.904644822 | 10.62715844 | 8.425768239 | 2.14E-12 | 1.60E-10 | 17.92367403 |
| IDO1 | -1.17303312 | 8.875366535 | -3.920182545 | 0.000196869 | 0.000687725 | 0.116242713 |
| IKBKAP | 0.577262813 | 9.333599451 | 4.923087705 | 5.08E-06 | 3.36E-05 | 3.612127749 |
| IPO8 | 0.616309492 | 7.304776 | 4.627775704 | 1.56E-05 | 8.43E-05 | 2.536512425 |
| IQSEC3 | -0.576942592 | 5.466797352 | -6.347294867 | 1.62E-08 | 2.80E-07 | 9.189987191 |
| IRF1 | -0.619843069 | 10.10376199 | -3.598903455 | 0.000577004 | 0.00167752 | -0.897406989 |
| ISG15 | -0.511628521 | 9.835082479 | -3.872459544 | 0.000231725 | 0.000783818 | -0.037971687 |
| IVL | 0.593975926 | 5.660539 | 2.524036452 | 0.013763334 | 0.025026796 | -3.811698996 |
| IWS1 | 0.558758913 | 7.968943127 | 3.892129421 | 0.000216696 | 0.000743275 | 0.025441003 |
| JAK2 | 0.532540012 | 8.266212437 | 2.804170513 | 0.006450543 | 0.013029254 | -3.129970118 |
| JUND | -1.002183572 | 11.82530227 | -6.405243115 | 1.27E-08 | 2.31E-07 | 9.427142627 |
| KCNS3 | -0.587487129 | 9.689819887 | -4.815174991 | 7.68E-06 | 4.75E-05 | 3.215090078 |
| KCTD16 | -1.490137315 | 5.611065887 | -7.098417046 | 6.66E-10 | 1.99E-08 | 12.30318782 |
| KIAA0430 | 0.596411945 | 10.73580265 | 4.623525689 | 1.58E-05 | 8.54E-05 | 2.521290026 |
| KIAA1429 | 0.519023418 | 9.95589462 | 4.442859528 | 3.08E-05 | 0.000147761 | 1.881375853 |
| KIAA2022 | 0.600156544 | 4.786732831 | 3.871323083 | 0.000232623 | 0.000786153 | -0.041629026 |
| KIF4A | 0.851531055 | 5.565599704 | 8.023547676 | 1.23E-11 | 6.85E-10 | 16.21330045 |
| KLF15 | -0.783621432 | 7.619953183 | -3.8321424 | 0.000265707 | 0.000880284 | -0.16728673 |
| KLF2 | -0.546144774 | 12.01329921 | -5.067609584 | 2.91E-06 | 2.10E-05 | 4.150636145 |
| KLF5 | -0.659445783 | 9.476255944 | -5.438388318 | 6.74E-07 | 6.24E-06 | 5.564704248 |
| KLF6 | -0.50363495 | 12.11907079 | -4.826739834 | 7.35E-06 | 4.58E-05 | 3.257426126 |
| KLF7 | -0.635506934 | 8.828228338 | -5.165438599 | 1.98E-06 | 1.53E-05 | 4.519353287 |
| KPNB1 | 0.704597724 | 11.07229768 | 5.198899415 | 1.74E-06 | 1.37E-05 | 4.646213507 |
| LDHB | 0.740663979 | 12.68654707 | 5.868644737 | 1.18E-07 | 1.45E-06 | 7.256394495 |
| LEO1 | 0.908410032 | 9.117341761 | 5.987151494 | 7.24E-08 | 9.62E-07 | 7.730558948 |
| LMNB1 | 0.6273224 | 7.938897704 | 4.178115169 | 8.01E-05 | 0.000327221 | 0.970457534 |
| LRRN1 | 1.465045575 | 6.437241282 | 9.197651321 | 7.54E-14 | 9.85E-12 | 21.20213093 |
| LSM8 | 0.635211891 | 9.387822817 | 4.970792389 | 4.23E-06 | 2.86E-05 | 3.789042471 |
| MAGEA4 | -0.572660239 | 4.160002789 | -2.727772974 | 0.007972072 | 0.015579019 | -3.321705438 |
| MAP2K3 | -0.558211999 | 10.86748524 | -5.852616376 | 1.26E-07 | 1.54E-06 | 7.19251535 |
| MAP2K7 | -0.605011054 | 6.191042944 | -7.250808086 | 3.46E-10 | 1.16E-08 | 12.94316772 |
| MAP3K5 | 0.637151862 | 10.11636776 | 5.00025203 | 3.78E-06 | 2.61E-05 | 3.898711804 |
| MAP4K2 | -0.92388994 | 7.766376338 | -6.595594748 | 5.68E-09 | 1.21E-07 | 10.21015094 |
| MAPK3 | -0.79060034 | 8.964578662 | -6.140805988 | 3.83E-08 | 5.71E-07 | 8.350004977 |
| MAT2B | 0.50566622 | 10.18597594 | 4.365096237 | 4.09E-05 | 0.000186108 | 1.610404562 |
| MCM4 | 0.770138937 | 7.113590535 | 8.751741634 | 5.20E-13 | 5.02E-11 | 19.30984568 |
| MCM6 | 0.851166925 | 9.213468225 | 7.993144271 | 1.40E-11 | 7.65E-10 | 16.08412459 |
| MDH1 | 0.622847261 | 10.25876731 | 5.013864978 | 3.58E-06 | 2.49E-05 | 3.949494914 |
| MDN1 | 0.588625564 | 7.38438531 | 4.501807153 | 2.48E-05 | 0.000123734 | 2.088599496 |
| MEI1 | 0.501797049 | 8.667319268 | 3.524565328 | 0.000734367 | 0.002053055 | -1.123439941 |
| MET | 0.967011384 | 10.51284158 | 4.997534934 | 3.82E-06 | 2.63E-05 | 3.888583682 |
| MICA | -1.070285829 | 8.273875211 | -7.675932785 | 5.53E-11 | 2.43E-09 | 14.7382735 |
| MKI67 | 0.940470333 | 5.122812746 | 6.251528727 | 2.41E-08 | 3.92E-07 | 8.799398621 |
| MLX | -0.522603875 | 9.280068423 | -5.495101228 | 5.37E-07 | 5.14E-06 | 5.78477928 |
| MORC3 | 0.52260799 | 9.394175493 | 3.425222085 | 0.001008876 | 0.002675323 | -1.420267516 |
| MPHOSPH6 | 0.778830303 | 7.751758648 | 4.582839417 | 1.84E-05 | 9.64E-05 | 2.375949038 |
| MRPS11 | -0.625630001 | 9.133513366 | -4.403159146 | 3.56E-05 | 0.000165529 | 1.742692521 |
| MSH2 | 0.803475117 | 8.324282831 | 5.122217295 | 2.35E-06 | 1.76E-05 | 4.356046791 |
| MSH3 | 0.723336071 | 8.459200324 | 5.624075031 | 3.19E-07 | 3.36E-06 | 6.288686327 |
| MSH6 | 0.534380215 | 9.79003507 | 4.568653873 | 1.94E-05 | 0.000100643 | 2.325440581 |
| MT1X | -0.778980609 | 12.42602513 | -3.479542251 | 0.00084862 | 0.002316463 | -1.258714913 |
| MTF2 | 0.634714679 | 8.799525028 | 4.172599985 | 8.16E-05 | 0.00033286 | 0.951838446 |
| MYEF2 | 0.745931503 | 7.173154718 | 3.574957218 | 0.000623817 | 0.001791076 | -0.970579067 |
| MYO1C | -0.502508562 | 12.24961362 | -6.316456426 | 1.84E-08 | 3.13E-07 | 9.06402512 |
| NACA | 0.524399131 | 12.25150192 | 4.427065596 | 3.27E-05 | 0.000154591 | 1.826118212 |
| NAP1L1 | 0.702250886 | 11.02161786 | 4.553400897 | 2.05E-05 | 0.000105413 | 2.271227876 |
| NASP | 0.572237452 | 9.554757901 | 4.741022592 | 1.02E-05 | 5.96E-05 | 2.94488659 |
| NBN | 0.602846906 | 8.405992606 | 3.672535817 | 0.000453085 | 0.001369564 | -0.670290949 |
| NCAPG | 1.063925888 | 6.435435648 | 6.029604559 | 6.07E-08 | 8.32E-07 | 7.901196111 |
| NCBP2 | 0.65379285 | 8.916040789 | 5.658273606 | 2.78E-07 | 2.98E-06 | 6.423071174 |
| NCL | 0.778522629 | 12.00751745 | 4.944954154 | 4.67E-06 | 3.12E-05 | 3.693115884 |
| NDC80 | 0.547437445 | 5.960599972 | 5.099889696 | 2.56E-06 | 1.89E-05 | 4.271934968 |
| NEK2 | 0.85482612 | 4.856048972 | 5.953634862 | 8.31E-08 | 1.08E-06 | 7.596125629 |
| NFATC1 | -0.602810753 | 9.576895577 | -5.251856445 | 1.41E-06 | 1.16E-05 | 4.847746748 |
| NFKB2 | -0.649398384 | 8.154775437 | -4.556831143 | 2.03E-05 | 0.000104296 | 2.283411051 |
| NIPBL | 0.59627885 | 9.377517254 | 3.629164077 | 0.00052261 | 0.001547601 | -0.804454352 |
| NOP10 | -0.8414631 | 11.13497182 | -5.983490085 | 7.35E-08 | 9.74E-07 | 7.715860928 |
| NOS3 | -0.528181702 | 7.712710296 | -3.99452315 | 0.000152385 | 0.000556994 | 0.358902482 |
| NPM1 | 0.601241016 | 11.48022221 | 4.59409974 | 1.76E-05 | 9.33E-05 | 2.416103283 |
| NR2F6 | -0.542856622 | 9.032269944 | -3.611324151 | 0.000554057 | 0.001623269 | -0.859319286 |
| NUP107 | 0.783148307 | 9.589730718 | 4.251942523 | 6.15E-05 | 0.000262497 | 1.221131493 |
| OPA1 | 0.876110883 | 9.036064507 | 4.799951406 | 8.14E-06 | 4.97E-05 | 3.159439861 |
| OXCT1 | 0.871125864 | 8.386130296 | 7.02885095 | 8.98E-10 | 2.56E-08 | 12.01180236 |
| PABPC1 | 0.626125483 | 12.94318006 | 6.287864168 | 2.07E-08 | 3.46E-07 | 8.947393935 |
| PABPC4 | 0.656724874 | 9.33394169 | 6.624840162 | 5.02E-09 | 1.09E-07 | 10.33096011 |
| PACSIN3 | -0.543694203 | 8.170583775 | -4.360673839 | 4.16E-05 | 0.000188777 | 1.59507777 |
| PAK4 | -0.570213429 | 8.144308507 | -6.669143924 | 4.16E-09 | 9.38E-08 | 10.51421586 |
| PARP1 | 0.574368818 | 10.20506869 | 7.038403407 | 8.62E-10 | 2.49E-08 | 12.05178364 |
| PBRM1 | 0.753202377 | 8.022722211 | 6.457972347 | 1.02E-08 | 1.95E-07 | 9.643446398 |
| PC | 0.586270974 | 8.587122887 | 6.29572148 | 2.01E-08 | 3.37E-07 | 8.979429698 |
| PCNA | 0.689931573 | 9.199648113 | 7.23265058 | 3.74E-10 | 1.24E-08 | 12.8668008 |
| PCP4 | 1.346801847 | 6.348743535 | 5.790093913 | 1.63E-07 | 1.90E-06 | 6.943936211 |
| PCSK9 | -0.871248193 | 7.953146056 | -3.966959979 | 0.000167618 | 0.000601779 | 0.268589744 |
| PDCD4 | 1.091841019 | 10.37871261 | 5.61805576 | 3.27E-07 | 3.43E-06 | 6.265066022 |
| PDE1B | -0.570106144 | 7.62660062 | -4.5672587 | 1.95E-05 | 0.000101096 | 2.320477643 |
| PDGFRA | 0.878111375 | 9.811267732 | 4.721088616 | 1.10E-05 | 6.34E-05 | 2.872623397 |
| PDK1 | 0.859216062 | 8.518091676 | 4.678960353 | 1.28E-05 | 7.23E-05 | 2.720434013 |
| PDLIM2 | -0.882150895 | 9.399255239 | -7.013817321 | 9.57E-10 | 2.71E-08 | 11.94889996 |
| PEA15 | -0.574852436 | 11.74940668 | -6.297490494 | 1.99E-08 | 3.36E-07 | 8.986643898 |
| PEBP1 | -0.743021276 | 11.89651368 | -6.003985222 | 6.75E-08 | 9.08E-07 | 7.798173231 |
| PFDN1 | -0.523118925 | 9.832037521 | -7.290334064 | 2.92E-10 | 1.00E-08 | 13.10950515 |
| PGM2 | 0.601305873 | 9.056366394 | 4.790777083 | 8.42E-06 | 5.10E-05 | 3.12594677 |
| PGS1 | -0.643631114 | 8.585259056 | -5.412752948 | 7.46E-07 | 6.80E-06 | 5.465538044 |
| PHF1 | -0.632315303 | 9.676391549 | -5.496511547 | 5.34E-07 | 5.11E-06 | 5.790263996 |
| PHYHD1 | -0.601820843 | 9.131519408 | -4.657846935 | 1.39E-05 | 7.69E-05 | 2.644435355 |
| PI4K2B | 0.767813594 | 8.87699138 | 4.185281283 | 7.81E-05 | 0.000320386 | 0.994672546 |
| PIGN | 0.80910674 | 7.083507746 | 5.497901328 | 5.31E-07 | 5.09E-06 | 5.795669403 |
| PIK3C2B | -0.638077251 | 10.34149723 | -6.551784102 | 6.84E-09 | 1.40E-07 | 10.0294195 |
| PIK3CA | 0.655406699 | 8.906216535 | 3.806844081 | 0.00028941 | 0.000943137 | -0.247971784 |
| PIK3CB | 0.808805465 | 7.949387028 | 4.861610946 | 6.43E-06 | 4.09E-05 | 3.385394101 |
| PIK3CG | 0.757539781 | 6.565775155 | 4.564309462 | 1.97E-05 | 0.000102063 | 2.309989311 |
| PKIB | 0.954412342 | 6.213506 | 5.23852538 | 1.49E-06 | 1.21E-05 | 4.796927636 |
| PLCB4 | 0.756975456 | 7.532077254 | 4.614479717 | 1.63E-05 | 8.79E-05 | 2.488915112 |
| PLCD3 | -0.514398822 | 7.019946577 | -3.588426155 | 0.00059706 | 0.001725892 | -0.929464244 |
| PLCH1 | 0.582759003 | 6.718174028 | 3.275987023 | 0.001608879 | 0.003980024 | -1.854565373 |
| PLCL2 | 0.548555193 | 7.357572211 | 4.078623929 | 0.000113693 | 0.000437196 | 0.636914256 |
| PLOD2 | 0.975219139 | 9.219078676 | 5.366834272 | 8.96E-07 | 7.96E-06 | 5.288404924 |
| PML | -0.560296967 | 9.315442704 | -7.553745465 | 9.38E-11 | 3.78E-09 | 14.22110524 |
| PNMT | -0.697513038 | 7.718550887 | -3.295343819 | 0.001515462 | 0.003786655 | -1.799036748 |
| POLA1 | 0.54475992 | 8.676748141 | 4.290927786 | 5.35E-05 | 0.000233957 | 1.354565331 |
| POLR1C | 0.565529465 | 9.253290014 | 4.536652201 | 2.18E-05 | 0.000111252 | 2.211814778 |
| POLR2B | 1.007470835 | 10.28636951 | 6.526699164 | 7.60E-09 | 1.52E-07 | 9.926072287 |
| POT1 | 0.543596624 | 7.877959225 | 4.13175891 | 9.43E-05 | 0.00037451 | 0.814430449 |
| PPM1F | -0.512323769 | 8.038514521 | -4.50072694 | 2.49E-05 | 0.000124146 | 2.084788271 |
| PPP1CC | 0.669908721 | 11.64897138 | 5.744671481 | 1.96E-07 | 2.21E-06 | 6.763953065 |
| PPP1R1B | -0.977376868 | 7.521962634 | -5.97734712 | 7.54E-08 | 9.93E-07 | 7.691207933 |
| PRDM16 | -0.607314709 | 7.933825958 | -6.167643321 | 3.42E-08 | 5.24E-07 | 8.458706153 |
| PRDX3 | 0.807186303 | 10.25596676 | 5.954262959 | 8.29E-08 | 1.08E-06 | 7.598642551 |
| PRDX4 | 0.787230376 | 10.68893068 | 7.814508808 | 3.03E-11 | 1.46E-09 | 15.3257281 |
| PRKAR2B | 0.657745411 | 8.028654394 | 3.985089778 | 0.000157443 | 0.000573127 | 0.327948292 |
| PRKCQ | -0.718518632 | 7.544562944 | -5.458862426 | 6.21E-07 | 5.81E-06 | 5.644045345 |
| PRKD2 | -0.619037903 | 9.532696803 | -4.946122706 | 4.65E-06 | 3.11E-05 | 3.697448921 |
| PRKDC | 0.823591285 | 8.996540592 | 6.370232396 | 1.47E-08 | 2.60E-07 | 9.283788755 |
| PRPF4B | 0.713969256 | 9.033751803 | 3.543452369 | 0.000690917 | 0.001948123 | -1.066326161 |
| PRX | -1.427671044 | 9.47785107 | -7.55465797 | 9.34E-11 | 3.77E-09 | 14.2249643 |
| PSAT1 | 1.954138315 | 7.343226254 | 13.15088977 | 5.49E-21 | 1.39E-17 | 37.26852413 |
| PSMB6 | -0.620669838 | 11.84439575 | -4.90903801 | 5.36E-06 | 3.52E-05 | 3.560185108 |
| PSMD8 | -0.508214169 | 10.6490142 | -4.01805483 | 0.000140441 | 0.000520496 | 0.436321418 |
| PTPN5 | -0.791319904 | 6.286575831 | -5.746711092 | 1.94E-07 | 2.20E-06 | 6.772023681 |
| PURA | -0.529678528 | 10.97193672 | -4.60558114 | 1.69E-05 | 9.05E-05 | 2.457101405 |
| RAB1B | -0.548551057 | 10.39264814 | -4.646460862 | 1.45E-05 | 7.96E-05 | 2.603527385 |
| RAB5C | -0.522935169 | 10.76350999 | -4.513298992 | 2.38E-05 | 0.000119704 | 2.12917696 |
| RAB7A | -0.680866217 | 9.242660535 | -6.381303155 | 1.40E-08 | 2.51E-07 | 9.32909545 |
| RAB9B | 0.516814725 | 5.569281859 | 4.711260414 | 1.14E-05 | 6.51E-05 | 2.837054063 |
| RACGAP1 | 0.532575614 | 8.078251155 | 5.854506237 | 1.25E-07 | 1.53E-06 | 7.20004396 |
| RAD17 | 0.611228771 | 8.389774901 | 4.052115373 | 0.000124734 | 0.000471984 | 0.548890454 |
| RAD21 | 0.729604162 | 10.99767721 | 3.928656911 | 0.00019123 | 0.0006719 | 0.143755711 |
| RAD50 | 1.10019487 | 8.666426915 | 5.543031371 | 4.43E-07 | 4.41E-06 | 5.971498951 |
| RAD51AP1 | 1.050408885 | 5.891504225 | 6.241492273 | 2.52E-08 | 4.07E-07 | 8.758564179 |
| RAD54B | 0.677296307 | 6.015012423 | 4.578264845 | 1.87E-05 | 9.78E-05 | 2.359651598 |
| RAP1GDS1 | 0.588747283 | 8.493278648 | 4.854686969 | 6.60E-06 | 4.19E-05 | 3.359947641 |
| RARRES2 | -0.535854417 | 11.48693442 | -4.427622514 | 3.26E-05 | 0.00015447 | 1.828064764 |
| RB1CC1 | 0.70849665 | 9.611383634 | 3.724895373 | 0.000380862 | 0.001185618 | -0.506872854 |
| RBBP5 | 0.518253366 | 6.496408366 | 5.510491337 | 5.05E-07 | 4.89E-06 | 5.844662277 |
| RBL1 | 0.52566294 | 6.138612268 | 5.311491182 | 1.12E-06 | 9.50E-06 | 5.075779176 |
| RECQL | 0.834676962 | 8.736256648 | 4.886656548 | 5.85E-06 | 3.78E-05 | 3.477592651 |
| RELA | -0.681028603 | 9.297716423 | -5.336431708 | 1.01E-06 | 8.76E-06 | 5.171481521 |
| RELB | -0.601361011 | 10.4814002 | -4.362456011 | 4.13E-05 | 0.000187786 | 1.601253189 |
| REV3L | 0.538354068 | 9.725232479 | 3.442656626 | 0.00095456 | 0.002559066 | -1.368614642 |
| RHBDL2 | 1.074881144 | 6.22568362 | 7.544008422 | 9.78E-11 | 3.92E-09 | 14.17992972 |
| RIF1 | 0.502326206 | 8.049985803 | 3.075278552 | 0.002952993 | 0.006684947 | -2.415790903 |
| RMI1 | 0.761881375 | 7.898779831 | 5.08910287 | 2.67E-06 | 1.96E-05 | 4.231360949 |
| RNF20 | 0.71754226 | 8.79913169 | 6.032055422 | 6.01E-08 | 8.26E-07 | 7.911059298 |
| ROCK1 | 0.783306194 | 9.047517634 | 4.262487444 | 5.93E-05 | 0.000254774 | 1.257151537 |
| ROCK2 | 0.769379761 | 9.113803521 | 4.496662454 | 2.53E-05 | 0.000125564 | 2.070452486 |
| RPAP3 | 0.772190805 | 7.182265169 | 4.614149617 | 1.64E-05 | 8.80E-05 | 2.487734358 |
| RPL36 | -0.500280143 | 11.18287469 | -3.557611114 | 0.000659955 | 0.001875486 | -1.023369633 |
| RPRD1A | 0.572799395 | 9.098828408 | 4.267947546 | 5.81E-05 | 0.000250546 | 1.275823489 |
| RPS14 | -0.614441715 | 12.9759628 | -6.392821427 | 1.34E-08 | 2.42E-07 | 9.376256528 |
| RPS28 | -0.605986651 | 13.45801534 | -4.914523529 | 5.25E-06 | 3.45E-05 | 3.580456635 |
| RPS4Y1 | 2.853367809 | 9.828894901 | 3.671117845 | 0.000455212 | 0.001374347 | -0.674694542 |
| RPS4Y2 | 1.682029968 | 8.111687408 | 2.937841685 | 0.004413416 | 0.009389048 | -2.784320091 |
| RRM1 | 0.715580709 | 9.811808338 | 5.477668216 | 5.76E-07 | 5.44E-06 | 5.717030099 |
| RSBN1L | 0.557846053 | 6.965613169 | 3.92291133 | 0.000195036 | 0.000682897 | 0.125097816 |
| RUNX2 | 0.526230268 | 7.447608803 | 4.016249577 | 0.000141325 | 0.000523268 | 0.430371913 |
| RYR2 | 0.59947908 | 7.310115761 | 3.43642206 | 0.000973658 | 0.002601303 | -1.387107313 |
| S100A8 | -1.060817137 | 11.4224868 | -4.144610753 | 9.01E-05 | 0.000360357 | 0.85758023 |
| SART3 | 0.565391442 | 8.895966958 | 5.67676267 | 2.58E-07 | 2.79E-06 | 6.495855068 |
| SEC63 | 0.632508592 | 10.59041454 | 4.799866231 | 8.14E-06 | 4.97E-05 | 3.159128757 |
| SERBP1 | 0.644653452 | 9.967890366 | 6.714761271 | 3.43E-09 | 7.94E-08 | 10.70320154 |
| SETX | 0.563689591 | 9.853073704 | 4.131160616 | 9.45E-05 | 0.000375104 | 0.812423705 |
| SF3B4 | -0.612358123 | 5.83771562 | -3.87212232 | 0.000231991 | 0.000784368 | -0.039057007 |
| SH3GL2 | -1.373190718 | 5.754945239 | -9.332691658 | 4.21E-14 | 6.25E-12 | 21.77368814 |
| SIRPD | -0.614990894 | 5.186653577 | -3.860264413 | 0.000241538 | 0.000813741 | -0.07718099 |
| SKIV2L2 | 0.908909323 | 10.55869237 | 5.118043456 | 2.39E-06 | 1.79E-05 | 4.340310189 |
| SLC25A19 | -0.593824341 | 7.870737268 | -3.970651367 | 0.000165496 | 0.00059614 | 0.280661594 |
| SLC39A10 | 0.843427785 | 8.305153521 | 4.552615874 | 2.06E-05 | 0.000105651 | 2.268440432 |
| SLTM | 0.699192105 | 10.12914289 | 3.398476325 | 0.001097906 | 0.002874673 | -1.499138338 |
| SMARCA1 | 0.649369318 | 9.846385718 | 3.879673352 | 0.000226101 | 0.000769261 | -0.014739913 |
| SMARCA5 | 0.911068625 | 8.777524592 | 5.050005948 | 3.11E-06 | 2.22E-05 | 4.084640972 |
| SMC3 | 0.792030385 | 9.609353704 | 4.400023966 | 3.60E-05 | 0.000167174 | 1.731771065 |
| SMC5 | 0.601197046 | 8.684724535 | 3.101791093 | 0.002729111 | 0.006259515 | -2.343202172 |
| SMC6 | 0.925276457 | 8.149473479 | 5.180194694 | 1.87E-06 | 1.46E-05 | 4.575252035 |
| SMCHD1 | 0.557929109 | 9.529881887 | 3.389169146 | 0.00113059 | 0.002951576 | -1.526479301 |
| SND1 | 0.589810077 | 11.61406221 | 7.866161942 | 2.43E-11 | 1.20E-09 | 15.54490731 |
| SNRPA1 | 0.524929962 | 9.049664127 | 4.192451134 | 7.61E-05 | 0.000313988 | 1.018925503 |
| SNRPB | -0.789915074 | 10.12675799 | -5.627553881 | 3.15E-07 | 3.32E-06 | 6.302342212 |
| SNW1 | 0.573981022 | 10.26065379 | 4.307074315 | 5.05E-05 | 0.000222737 | 1.410041596 |
| SNX22 | -0.973099147 | 7.263576789 | -8.697384289 | 6.58E-13 | 6.08E-11 | 19.07879578 |
| SOAT1 | 0.796523033 | 8.893260042 | 6.548312002 | 6.94E-09 | 1.41E-07 | 10.01510889 |
| SSB | 0.661149644 | 10.76887893 | 3.943586147 | 0.00018167 | 0.000643686 | 0.192318775 |
| ST6GAL1 | 0.556642523 | 10.89399468 | 4.239139505 | 6.44E-05 | 0.000272726 | 1.177470066 |
| STAG1 | 0.78981771 | 7.668671113 | 4.420180764 | 3.35E-05 | 0.000157838 | 1.802065789 |
| SUPT16H | 0.719560385 | 9.001785014 | 5.699362031 | 2.35E-07 | 2.58E-06 | 6.584942126 |
| SYAP1 | 0.528754201 | 9.372121479 | 3.464611559 | 0.000890088 | 0.002413318 | -1.303301511 |
| SYDE1 | -0.572006832 | 7.669426535 | -4.409774345 | 3.48E-05 | 0.000162343 | 1.765751398 |
| SYK | 0.568588321 | 9.355125225 | 5.840792449 | 1.32E-07 | 1.60E-06 | 7.145432135 |
| SYNE2 | 0.528193833 | 8.844850239 | 3.440512219 | 0.000961089 | 0.002572087 | -1.374977999 |
| TAF15 | 0.684055303 | 11.21796541 | 5.298286092 | 1.18E-06 | 9.89E-06 | 5.025187574 |
| TAGLN | 0.762287044 | 12.4007071 | 6.03589847 | 5.92E-08 | 8.18E-07 | 7.9265278 |
| TBCA | 0.546992351 | 11.53919841 | 3.914463171 | 0.000200765 | 0.000698114 | 0.097695921 |
| TBX2 | -0.638813721 | 11.03771214 | -4.626720577 | 1.56E-05 | 8.46E-05 | 2.532732539 |
| TCEA1 | 0.69087957 | 10.72779923 | 4.710274265 | 1.14E-05 | 6.53E-05 | 2.833487249 |
| TCP1 | 0.63067475 | 11.54620497 | 5.10440753 | 2.52E-06 | 1.87E-05 | 4.288940524 |
| TDRD10 | -0.902515183 | 7.775787873 | -10.59907851 | 1.87E-16 | 7.47E-14 | 27.0763195 |
| TEAD4 | -0.950254325 | 8.690437901 | -6.918595571 | 1.44E-09 | 3.84E-08 | 11.55107026 |
| TF | 0.804551589 | 6.921991563 | 3.644786837 | 0.000496473 | 0.001483249 | -0.756254499 |
| TFAM | 0.557983652 | 8.106251803 | 3.971868071 | 0.000164802 | 0.000594513 | 0.284642127 |
| TFE3 | -0.60497475 | 8.991019338 | -5.391169372 | 8.13E-07 | 7.33E-06 | 5.38219871 |
| TFEC | 0.579387721 | 6.635838915 | 3.615257616 | 0.000546972 | 0.0016078 | -0.847238419 |
| TFRC | 0.766689652 | 11.34056525 | 3.715407116 | 0.000393078 | 0.001215093 | -0.536603269 |
| THG1L | 0.537720542 | 8.211876563 | 6.623563117 | 5.05E-09 | 1.10E-07 | 10.32568211 |
| THOC1 | 0.627211491 | 8.102930563 | 3.917157932 | 0.00019892 | 0.000692585 | 0.1064323 |
| THOC6 | -0.743142461 | 8.504057254 | -5.382253349 | 8.43E-07 | 7.55E-06 | 5.347813049 |
| TNKS2 | 0.521505283 | 8.854820479 | 3.180047542 | 0.002157167 | 0.005093471 | -2.126176404 |
| TNNC1 | -1.911948509 | 8.929490803 | -8.535802162 | 1.33E-12 | 1.07E-10 | 18.39170614 |
| TNPO2 | -0.678356915 | 7.467557592 | -5.556773441 | 4.19E-07 | 4.21E-06 | 6.025153807 |
| TOP2A | 1.539564882 | 6.169958944 | 9.939592682 | 3.10E-15 | 7.22E-13 | 24.32971365 |
| TOP2B | 0.969549792 | 10.8712512 | 4.794964067 | 8.29E-06 | 5.03E-05 | 3.141228265 |
| TOPBP1 | 0.698584183 | 7.924928085 | 4.571623181 | 1.92E-05 | 9.97E-05 | 2.336005843 |
| TP53BP1 | 0.889853688 | 9.331284197 | 8.795331632 | 4.30E-13 | 4.26E-11 | 19.49508434 |
| TPR | 0.768137516 | 10.03443218 | 4.286210806 | 5.44E-05 | 0.000237343 | 1.338382068 |
| TRIM22 | 0.707506985 | 10.14221054 | 4.432032976 | 3.21E-05 | 0.000152238 | 1.843485263 |
| TRIM23 | 0.882959031 | 6.42732469 | 5.35327923 | 9.46E-07 | 8.29E-06 | 5.236239066 |
| TRIP10 | -0.673230035 | 9.992696254 | -6.949404097 | 1.26E-09 | 3.42E-08 | 11.67967221 |
| TRPS1 | 0.984551685 | 8.349822324 | 6.515408594 | 7.97E-09 | 1.58E-07 | 9.879589124 |
| TUBB1 | -0.57779192 | 4.497323085 | -3.064914162 | 0.003045074 | 0.006855491 | -2.444037332 |
| TUBB2B | 0.603843868 | 6.164588521 | 2.544904869 | 0.013031195 | 0.023879804 | -3.762962226 |
| TUBB6 | -0.547651004 | 11.70423037 | -5.869670181 | 1.17E-07 | 1.45E-06 | 7.260483377 |
| UAP1 | 0.512720794 | 9.645982845 | 2.923515979 | 0.004599103 | 0.009738936 | -2.82197409 |
| UBR5 | 0.752957947 | 8.779698099 | 4.645464811 | 1.46E-05 | 7.98E-05 | 2.599951334 |
| UBXN2B | 0.613912663 | 7.736370268 | 4.087608988 | 0.000110169 | 0.000426132 | 0.666831446 |
| UCHL1 | 0.824124342 | 8.945437099 | 6.163993201 | 3.48E-08 | 5.30E-07 | 8.443913228 |
| UEVLD | 0.708278831 | 7.456662887 | 5.039036814 | 3.25E-06 | 2.30E-05 | 4.043573643 |
| UGP2 | 0.827301179 | 10.39368439 | 5.182611123 | 1.86E-06 | 1.45E-05 | 4.584412858 |
| UPF2 | 0.720308072 | 8.026885746 | 4.258019003 | 6.02E-05 | 0.000258008 | 1.241881414 |
| UPF3A | 0.523902973 | 9.668423718 | 3.863763785 | 0.000238682 | 0.000805015 | -0.065938269 |
| UPF3B | 0.515971063 | 7.355974606 | 3.474071046 | 0.000863599 | 0.002353956 | -1.275069132 |
| USP33 | 0.58249554 | 9.34132393 | 4.401377843 | 3.59E-05 | 0.000166454 | 1.736486771 |
| USP7 | 0.503300725 | 10.28008465 | 4.482842358 | 2.66E-05 | 0.000130914 | 2.021762421 |
| USP8 | 0.545965392 | 8.468097211 | 3.473318472 | 0.000865679 | 0.002357504 | -1.277317245 |
| USP9X | 0.748814462 | 11.20896483 | 5.205222083 | 1.70E-06 | 1.35E-05 | 4.670226574 |
| UTY | 1.074590138 | 6.020261141 | 2.946689296 | 0.004302225 | 0.009191228 | -2.760992466 |
| VAMP2 | -0.86922878 | 8.70096762 | -7.251861063 | 3.45E-10 | 1.16E-08 | 12.94759722 |
| VAMP5 | -0.631185982 | 10.76797966 | -5.321664517 | 1.07E-06 | 9.18E-06 | 5.11479297 |
| VPS28 | -0.604235781 | 11.64126035 | -6.431627403 | 1.13E-08 | 2.12E-07 | 9.535315894 |
| VPS36 | 0.509348162 | 9.173055915 | 5.046741208 | 3.15E-06 | 2.24E-05 | 4.072413656 |
| VPS54 | 0.586271405 | 8.48252593 | 3.653064021 | 0.000483133 | 0.00144854 | -0.730659573 |
| VRK1 | 0.591049067 | 7.865689563 | 3.848022364 | 0.000251789 | 0.000841659 | -0.116459207 |
| WARS | -0.645330658 | 11.61804738 | -4.831587596 | 7.21E-06 | 4.51E-05 | 3.275188053 |
| WDHD1 | 0.820431409 | 6.865464507 | 5.870768294 | 1.17E-07 | 1.44E-06 | 7.2648623 |
| WDR31 | 0.78589336 | 5.83587631 | 4.975770651 | 4.15E-06 | 2.81E-05 | 3.807552838 |
| WDR5B | 1.033926333 | 6.251894423 | 6.794039737 | 2.45E-09 | 5.99E-08 | 11.03232625 |
| WFS1 | -1.170682181 | 9.815009028 | -9.627896299 | 1.18E-14 | 2.10E-12 | 23.01983716 |
| WRN | 0.518587742 | 8.616217141 | 3.724563004 | 0.000381284 | 0.001186625 | -0.507915167 |
| XPO1 | 0.820369832 | 9.957126056 | 6.076600182 | 5.00E-08 | 7.18E-07 | 8.090550617 |
| XRCC4 | 0.668347074 | 7.818375451 | 5.269913524 | 1.32E-06 | 1.09E-05 | 4.916673556 |
| XRCC5 | 0.778380962 | 10.69205308 | 6.649465035 | 4.52E-09 | 1.01E-07 | 10.43278155 |
| XRN2 | 0.642245891 | 9.612001986 | 5.229900394 | 1.54E-06 | 1.24E-05 | 4.764079317 |
| ZC3H13 | 0.67364975 | 7.45656007 | 4.375205301 | 3.95E-05 | 0.000180321 | 1.645473704 |
| ZNF451 | 0.623200464 | 7.955859479 | 3.546923703 | 0.000683201 | 0.001929959 | -1.055805558 |
| ZNF790 | 0.822344637 | 6.747418155 | 6.559482467 | 6.62E-09 | 1.37E-07 | 10.06115586 |
| ZNHIT6 | 0.698206518 | 8.353539873 | 4.871977042 | 6.18E-06 | 3.96E-05 | 3.423525065 |
| ZSCAN4 | 1.009587941 | 4.060996873 | 5.542699406 | 4.43E-07 | 4.41E-06 | 5.970203476 |

**Script**

library(limma)

library(sva)

outFile="merge.txt"

files=dir()

files=grep("txt$", files, value=T)

geneList=list()

for(file in files){

if(file==outFile){next}

rt=read.table(file, header=T, sep="\t", check.names=F)

geneNames=as.vector(rt[,1])

uniqGene=unique(geneNames)

header=unlist(strsplit(file, "\\.|\\-"))

geneList[[header[1]]]=uniqGene

}

interGenes=Reduce(intersect, geneList)

allTab=data.frame()

batchType=c()

for(i in 1:length(files)){

inputFile=files[i]

header=unlist(strsplit(inputFile, "\\.|\\-"))

rt=read.table(inputFile, header=T, sep="\t", check.names=F)

rt=as.matrix(rt)

rownames(rt)=rt[,1]

exp=rt[,2:ncol(rt)]

dimnames=list(rownames(exp),colnames(exp))

data=matrix(as.numeric(as.matrix(exp)),nrow=nrow(exp),dimnames=dimnames)

rt=avereps(data)

qx=as.numeric(quantile(rt, c(0, 0.25, 0.5, 0.75, 0.99, 1.0), na.rm=T))

LogC=( (qx[5]>100) || ( (qx[6]-qx[1])>50 && qx[2]>0) )

if(LogC){

rt[rt<0]=0

rt=log2(rt+1)}

rt=normalizeBetweenArrays(rt)

if(i==1){

allTab=rt[interGenes,]

}else{

allTab=cbind(allTab, rt[interGenes,])

}

batchType=c(batchType, rep(i,ncol(rt)))

}

outTab=ComBat(allTab, batchType, par.prior=TRUE)

outTab=rbind(geneNames=colnames(outTab), outTab)

write.table(outTab, file="merge.txt", sep="\t", quote=F, col.names=F)

library(limma)

library(sva)

mergeFile="merge.preNorm.txt"

normalizeFile="merge.normalzie.txt"

files=c("GSE10667.txt", "GSE110147.txt")

geneList=list()

for(i in 1:length(files)){

fileName=files[i]

rt=read.table(fileName, header=T, sep="\t", check.names=F)

header=unlist(strsplit(fileName, "\\.|\\-"))

geneList[[header[1]]]=as.vector(rt[,1])

}

intersectGenes=Reduce(intersect, geneList)

allTab=data.frame()

batchType=c()

for(i in 1:length(files)){

fileName=files[i]

header=unlist(strsplit(fileName, "\\.|\\-"))

rt=read.table(fileName, header=T, sep="\t", check.names=F)

rt=as.matrix(rt)

rownames(rt)=rt[,1]

exp=rt[,2:ncol(rt)]

dimnames=list(rownames(exp),colnames(exp))

data=matrix(as.numeric(as.matrix(exp)), nrow=nrow(exp), dimnames=dimnames)

rt=avereps(data)

colnames(rt)=paste0(header[1], "_", colnames(rt))

qx=as.numeric(quantile(rt, c(0, 0.25, 0.5, 0.75, 0.99, 1.0), na.rm=T))

LogC=( (qx[5]>100) || ( (qx[6]-qx[1])>50 && qx[2]>0) )

if(LogC){

rt[rt<0]=0

rt=log2(rt+1)}

rt=normalizeBetweenArrays(rt)

#??????

if(i==1){

allTab=rt[intersectGenes,]

}else{

allTab=cbind(allTab, rt[intersectGenes,])

}

batchType=c(batchType, rep(header[1],ncol(rt)))

}

allTabOut=rbind(geneNames=colnames(allTab), allTab)

write.table(allTabOut, file=mergeFile, sep="\t", quote=F, col.names=F)

normalizeTab=ComBat(allTab, batchType, par.prior=TRUE)

normalizeTab=rbind(geneNames=colnames(normalizeTab), normalizeTab)

write.table(normalizeTab, file=normalizeFile, sep="\t", quote=F, col.names=F)

library(ggplot2)

bioPCA=function(inputFile=null, outFile=null){

rt=read.table(inputFile, header=T, sep="\t", check.names=F, row.names=1)

data=t(rt)

Project=gsub("(.*?)\\_.*", "\\1", rownames(data))

rownames(data)=gsub("(.*?)\\_(.*?)", "\\2", rownames(data))

data.pca=prcomp(data)

pcaPredict=predict(data.pca)

PCA=data.frame(PC1=pcaPredict[,1], PC2=pcaPredict[,2], Type=Project)

pdf(file=outFile, height=5, width=6)

p=ggplot(data = PCA, aes(PC1, PC2,PC3,PC4,PC5)) + geom_point(aes(color = Type)) +

scale_colour_manual(name="", values=c("blue", "red","green","gray","yellow"))+

theme_bw()+

theme(plot.margin=unit(rep(1.5,4),'lines'))+

theme(panel.grid.major = element_blank(), panel.grid.minor = element_blank())

print(p)

dev.off()

}

bioPCA(inputFile="merge.preNorm.txt", outFile="PCA.preNorm.pdf")

bioPCA(inputFile="merge.normalzie.txt", outFile="PCA.normalzie.pdf")

library(limma)

library(pheatmap)

inputFile="merge.txt"

logFCfilter=0.5

adj.P.Val.Filter=0.05

rt=read.table(inputFile, header=T, sep="\t", check.names=F)

rt=as.matrix(rt)

rownames(rt)=rt[,1]

exp=rt[,2:ncol(rt)]

dimnames=list(rownames(exp),colnames(exp))

data=matrix(as.numeric(as.matrix(exp)),nrow=nrow(exp),dimnames=dimnames)

data=avereps(data)

data=data[rowMeans(data)>0,]

sampleName1=c()

files=dir()

files=grep("s1.txt$", files, value=T)

for(file in files){

rt=read.table(file, header=F, sep="\t", check.names=F)

geneNames=as.vector(rt[,1])

uniqGene=unique(geneNames)

sampleName1=c(sampleName1, uniqGene)

}

sampleName2=c()

files=dir()

files=grep("s2.txt$", files, value=T)

for(file in files){

rt=read.table(file, header=F, sep="\t", check.names=F)

geneNames=as.vector(rt[,1])

uniqGene=unique(geneNames)

sampleName2=c(sampleName2, uniqGene)

}

ControlData=data[,sampleName1]

TreatData=data[,sampleName2]

data=cbind(ControlData,TreatData)

ControlNum=ncol(ControlData)

TreatNum=ncol(TreatData)

Type=c(rep("Control",ControlNum),rep("Treat",TreatNum))

design <- model.matrix(~0+factor(Type))

colnames(design) <- c("Control","Treat")

fit <- lmFit(data,design)

cont.matrix<-makeContrasts(Treat-Control,levels=design)

fit2 <- contrasts.fit(fit, cont.matrix)

fit2 <- eBayes(fit2)

allDiff=topTable(fit2,adjust='fdr',number=200000)

allDiffOut=rbind(id=colnames(allDiff),allDiff)

write.table(allDiffOut, file="all.txt", sep="\t", quote=F, col.names=F)

outData=rbind(id=paste0(colnames(data),"_",Type),data)

write.table(outData, file="normalize.txt", sep="\t", quote=F, col.names=F)

diffSig=allDiff[with(allDiff, (abs(logFC)>logFCfilter & adj.P.Val < adj.P.Val.Filter )), ]

diffSigOut=rbind(id=colnames(diffSig),diffSig)

write.table(diffSigOut, file="diff.txt", sep="\t", quote=F, col.names=F)

diffGeneExp=data[row.names(diffSig),]

diffGeneExpOut=rbind(id=paste0(colnames(diffGeneExp),"_",Type), diffGeneExp)

write.table(diffGeneExpOut, file="diffGeneExp.txt", sep="\t", quote=F, col.names=F)

geneNum=50

diffSig=diffSig[order(as.numeric(as.vector(diffSig$logFC))),]

diffGeneName=as.vector(rownames(diffSig))

diffLength=length(diffGeneName)

hmGene=c()

if(diffLength>(2*geneNum)){

hmGene=diffGeneName[c(1:geneNum,(diffLength-geneNum+1):diffLength)]

}else{

hmGene=diffGeneName

}

hmExp=data[hmGene,]

Type=c(rep("Control",ControlNum),rep("Treat",TreatNum))

names(Type)=colnames(data)

Type=as.data.frame(Type)

pdf(file="heatmap.pdf", width=10, height=8)

pheatmap(hmExp,

annotation=Type,

color = colorRampPalette(c("#1f77b4", "white", "#ff7f0e"))(50),

cluster_cols =F,

show_colnames = F,

scale="row",

fontsize = 8,

fontsize_row=7,

fontsize_col=8)

dev.off()

library(ggplot2)

logFCfilter=0.5

adj.P.Val.Filter=0.05

inputFile="all.txt"

rt=read.table(inputFile, header=T, sep="\t", check.names=F)

Sig=ifelse((rt$adj.P.Val<adj.P.Val.Filter) & (abs(rt$logFC)>logFCfilter), ifelse(rt$logFC>logFCfilter,"Up","Down"), "Not")

rt=cbind(rt, Sig=Sig)

p=ggplot(rt, aes(logFC, -log10(adj.P.Val)))+

geom_point(aes(col=Sig))+

scale_color_manual(values=c("#1f77b4", "gray", "#ff7f0e"))+

xlim(-5,5)+

labs(title = " ")+

geom_vline(xintercept=c(-logFCfilter,logFCfilter), col="black", cex=0.5, linetype=2)+

geom_hline(yintercept= -log10(adj.P.Val.Filter), col="black", cex=0.5, linetype=2)+

theme(plot.title=element_text(size=16, hjust=0.5, face="bold"))

p=p+theme_bw()

pdf(file="volcano.pdf", width=6, height=5.1)

print(p)

dev.off()

BMs=read.table("telomere.txt",header = T,sep = "\t")

nontumor=read.table("diffGeneExp.txt",header = T,sep = "\t")

BMs_gene=merge(BMs,nontumor,by="id")

write.table(BMs_gene,"telExp.txt",quote = F,row.names = F,sep = "\t")

library(clusterProfiler)

library(org.Hs.eg.db)

library(enrichplot)

library(ggplot2)

library(circlize)

library(RColorBrewer)

library(dplyr)

library(ComplexHeatmap)

library(stringr)

library(GOplot)

library(DOSE)

library(ggnewscale)

library(topGO)

pvalueFilter=0.05

qvalueFilter=0.05

colorSel="qvalue"

if(qvalueFilter>0.05){

colorSel="pvalue"

}

ontology.col=c("#00AFBB", "#E7B800", "#90EE90")

rt=read.table("telExp.txt", header=T, sep="\t", check.names=F)

genes=unique(as.vector(rt[,1]))

entrezIDs=mget(genes, org.Hs.egSYMBOL2EG, ifnotfound=NA)

entrezIDs=as.character(entrezIDs)

gene=entrezIDs[entrezIDs!="NA"]

kk=enrichGO(gene=gene, OrgDb=org.Hs.eg.db, pvalueCutoff=1, qvalueCutoff=1, ont="ALL", readable=T)

GO=as.data.frame(kk)

GO=GO[(GO$pvalue<pvalueFilter & GO$qvalue<qvalueFilter),]

write.table(GO, file="GO.txt", sep="\t", quote=F, row.names = F)

showNum=10

if(nrow(GO)<10){

showNum=nrow(GO)

}

pdf(file="barplot.pdf", width=9, height=10)

bar=barplot(kk, drop=TRUE, showCategory=showNum, label_format=130, split="ONTOLOGY", color=colorSel) + facet_grid(ONTOLOGY~., scale='free')

print(bar)

dev.off()

pdf(file="bubble.pdf", width=9, height=10)

bub=dotplot(kk, showCategory=showNum, orderBy="GeneRatio", label_format=130, split="ONTOLOGY", color=colorSel) + facet_grid(ONTOLOGY~., scale='free')

print(bub)

dev.off()

library(clusterProfiler)

library(org.Hs.eg.db)

library(enrichplot)

library(ggplot2)

library(circlize)

library(RColorBrewer)

library(dplyr)

pvalueFilter=0.05

qvalueFilter=0.05

colorSel="qvalue"

if(qvalueFilter>0.05){

colorSel="pvalue"

}

rt=read.table("telExp.txt", header=T, sep="\t", check.names=F)

genes=unique(as.vector(rt[,1]))

entrezIDs=mget(genes, org.Hs.egSYMBOL2EG, ifnotfound=NA)

entrezIDs=as.character(entrezIDs)

rt=data.frame(genes, entrezID=entrezIDs)

gene=entrezIDs[entrezIDs!="NA"]

kk <- enrichKEGG(gene=gene, organism="hsa", pvalueCutoff=1, qvalueCutoff=1)

KEGG=as.data.frame(kk)

KEGG$geneID=as.character(sapply(KEGG$geneID,function(x)paste(rt$genes[match(strsplit(x,"/")[[1]],as.character(rt$entrezID))],collapse="/")))

KEGG=KEGG[(KEGG$pvalue<pvalueFilter & KEGG$qvalue<qvalueFilter),]

write.table(KEGG, file="KEGG.txt", sep="\t", quote=F, row.names = F)

showNum=30

if(nrow(KEGG)<showNum){

showNum=nrow(KEGG)

}

pdf(file="barplot.pdf", width=8, height=10)

barplot(kk, drop=TRUE, showCategory=showNum, label_format=130, color=colorSel)

dev.off()

pdf(file="bubble.pdf", width = 8, height = 10)

dotplot(kk, showCategory=showNum, orderBy="GeneRatio", label_format=130, color=colorSel)

dev.off()

kk_read <- DOSE::setReadable(kk,OrgDb="org.Hs.eg.db",keyType='ENTREZID')

genelist <- as.numeric(rt[,2])

names(genelist) <- rt[,1]

pdf(file="cnetplot.pdf", width = 16, height = 12)

cnetplot(kk_read, foldChange=genelist, circular = TRUE, colorEdge = TRUE)

dev.off()

library("clusterProfiler")

library("org.Hs.eg.db")

library("enrichplot")

library("ggplot2")

library("GSEABase")

library("DOSE")

pvalueFilter=0.05

qvalueFilter=0.05

colorSel="qvalue"

if(qvalueFilter>0.05){

colorSel="pvalue"

}

rt=read.table("telExp.txt", header=T, sep="\t", check.names=F)

genes=as.vector(rt[,1])

entrezIDs=mget(genes, org.Hs.egSYMBOL2EG, ifnotfound=NA)

entrezIDs=as.character(entrezIDs)

rt=cbind(rt,entrezID=entrezIDs)

gene=entrezIDs[entrezIDs!="NA"]

kk=enrichDO(gene=gene, ont="DO", pvalueCutoff=1, qvalueCutoff=1, readable=TRUE)

DO=as.data.frame(kk)

DO=DO[(DO$pvalue<pvalueFilter & DO$qvalue<qvalueFilter),]

write.table(DO, file="DO.txt", sep="\t", quote=F, row.names = F)

showNum=30

if(nrow(DO)<showNum){

showNum=nrow(DO)

}

pdf(file="barplot.pdf", width=6, height=10)

barplot(kk, drop=TRUE, showCategory=showNum, color=colorSel)

dev.off()

pdf(file="bubble.pdf", width = 6, height = 10)

dotplot(kk, showCategory=showNum, orderBy="GeneRatio", color=colorSel)

dev.off()

library(limma)

library(gplots)

library(WGCNA)

expFile="normalize.txt"

rt=read.table(expFile, header=T, sep="\t", check.names=F)

rt=as.matrix(rt)

rownames(rt)=rt[,1]

exp=rt[,2:ncol(rt)]

dimnames=list(rownames(exp),colnames(exp))

data=matrix(as.numeric(as.matrix(exp)),nrow=nrow(exp),dimnames=dimnames)

data=avereps(data)

selectGenes=names(tail(sort(apply(data,1,sd)), n=round(nrow(data)*0.25)))

data=data[selectGenes,]

Type=gsub("(.*)\\_(.*)", "\\2", colnames(data))

conCount=length(Type[Type=="Control"])

treatCount=length(Type[Type=="Treat"])

datExpr0=t(data)

gsg = goodSamplesGenes(datExpr0, verbose = 3)

if (!gsg$allOK){

# Optionally, print the gene and sample names that were removed:

if (sum(!gsg$goodGenes)>0)

printFlush(paste("Removing genes:", paste(names(datExpr0)[!gsg$goodGenes], collapse = ", ")))

if (sum(!gsg$goodSamples)>0)

printFlush(paste("Removing samples:", paste(rownames(datExpr0)[!gsg$goodSamples], collapse = ", ")))

# Remove the offending genes and samples from the data:

datExpr0 = datExpr0[gsg$goodSamples, gsg$goodGenes]

}

sampleTree = hclust(dist(datExpr0), method = "average")

pdf(file = "01.sample_cluster.pdf", width = 12, height = 9)

par(cex = 0.6)

par(mar = c(0,4,2,0))

plot(sampleTree, main = "Sample clustering to detect outliers", sub="", xlab="", cex.lab = 1.5, cex.axis = 1.5, cex.main = 2)

abline(h = 100, col="red")

dev.off()

clust=cutreeStatic(sampleTree, cutHeight=100, minSize=10)

table(clust)

keepSamples=(clust==1)

datExpr0=datExpr0[keepSamples,]

traitData=data.frame(Con=c(rep(1,conCount),rep(0,treatCount)),

Treat=c(rep(0,conCount),rep(1,treatCount)))

row.names(traitData)=colnames(data)

fpkmSamples=rownames(datExpr0)

traitSamples=rownames(traitData)

sameSample=intersect(fpkmSamples,traitSamples)

datExpr0=datExpr0[sameSample,]

datTraits=traitData[sameSample,]

sampleTree2 = hclust(dist(datExpr0), method="average")

traitColors = numbers2colors(datTraits, signed = FALSE)

pdf(file="02.sample_heatmap.pdf", width=12, height=12)

plotDendroAndColors(sampleTree2, traitColors,

groupLabels = names(datTraits),

main = "Sample dendrogram and trait heatmap")

dev.off()

enableWGCNAThreads()

powers = c(1:20)

sft = pickSoftThreshold(datExpr0, powerVector = powers, verbose = 5)

pdf(file="03.scale_independence.pdf",width=10,height=6)

par(mfrow = c(1,2))

cex1 = 0.9

plot(sft$fitIndices[,1], -sign(sft$fitIndices[,3])*sft$fitIndices[,2],

xlab="Soft Threshold (power)",ylab="Scale Free Topology Model Fit,signed R^2",type="n",

main = paste("Scale independence"));

text(sft$fitIndices[,1], -sign(sft$fitIndices[,3])*sft$fitIndices[,2],

labels=powers,cex=cex1,col="red");

abline(h=0.85,col="red")

plot(sft$fitIndices[,1], sft$fitIndices[,5],

xlab="Soft Threshold (power)",ylab="Mean Connectivity", type="n",

main = paste("Mean connectivity"))

text(sft$fitIndices[,1], sft$fitIndices[,5], labels=powers, cex=cex1,col="red")

dev.off()

sft

softPower =sft$powerEstimate

adjacency = adjacency(datExpr0, power = softPower)

softPower

TOM = TOMsimilarity(adjacency)

dissTOM = 1-TOM

geneTree = hclust(as.dist(dissTOM), method = "average");

pdf(file="04.gene_clustering.pdf",width=12,height=9)

plot(geneTree, xlab="", sub="", main = "Gene clustering on TOM-based dissimilarity",

labels = FALSE, hang = 0.04)

dev.off()

minModuleSize = 100

dynamicMods = cutreeDynamic(dendro = geneTree, distM = dissTOM,

deepSplit = 2, pamRespectsDendro = FALSE,

minClusterSize = minModuleSize);

table(dynamicMods)

dynamicColors = labels2colors(dynamicMods)

table(dynamicColors)

pdf(file="05.Dynamic_Tree.pdf",width=8,height=6)

plotDendroAndColors(geneTree, dynamicColors, "Dynamic Tree Cut",

dendroLabels = FALSE, hang = 0.03,

addGuide = TRUE, guideHang = 0.05,

main = "Gene dendrogram and module colors")

dev.off()

MEList = moduleEigengenes(datExpr0, colors = dynamicColors)

MEs = MEList$eigengenes

MEDiss = 1-cor(MEs);

METree = hclust(as.dist(MEDiss), method = "average")

pdf(file="06.Clustering_module.pdf",width=7,height=6)

plot(METree, main = "Clustering of module eigengenes",

xlab = "", sub = "")

dev.off()

moduleColors=dynamicColors

nGenes = ncol(datExpr0)

nSamples = nrow(datExpr0)

select = sample(nGenes, size=1000)

selectTOM = dissTOM[select, select];

selectTree = hclust(as.dist(selectTOM), method="average")

selectColors = moduleColors[select]

#sizeGrWindow(9,9)

plotDiss=selectTOM^softPower

diag(plotDiss)=NA

myheatcol = colorpanel(250, "red", "orange", "lemonchiffon")

pdf(file="07.TOMplot.pdf", width=7, height=7)

TOMplot(plotDiss, selectTree, selectColors, main = "Network heatmap plot, selected genes", col=myheatcol)

dev.off()

moduleTraitCor = cor(MEs, datTraits, use = "p")

moduleTraitPvalue = corPvalueStudent(moduleTraitCor, nSamples)

pdf(file="08.Module_trait.pdf", width=4.5, height=5.5)

textMatrix = paste(signif(moduleTraitCor, 2), "\n(",

signif(moduleTraitPvalue, 1), ")", sep = "")

dim(textMatrix) = dim(moduleTraitCor)

par(mar = c(3.5, 8, 3, 3))

labeledHeatmap(Matrix = moduleTraitCor,

xLabels = names(datTraits),

yLabels = names(MEs),

ySymbols = names(MEs),

colorLabels = FALSE,

colors = blueWhiteRed(50),

textMatrix = textMatrix,

setStdMargins = FALSE,

cex.text = 0.7,

zlim = c(-1,1),

main = paste("Module-trait relationships"))

dev.off()

modNames = substring(names(MEs), 3)

geneModuleMembership = as.data.frame(cor(datExpr0, MEs, use = "p"))

MMPvalue = as.data.frame(corPvalueStudent(as.matrix(geneModuleMembership), nSamples))

names(geneModuleMembership) = paste("MM", modNames, sep="")

names(MMPvalue) = paste("p.MM", modNames, sep="")

traitNames=names(datTraits)

geneTraitSignificance = as.data.frame(cor(datExpr0, datTraits, use = "p"))

GSPvalue = as.data.frame(corPvalueStudent(as.matrix(geneTraitSignificance), nSamples))

names(geneTraitSignificance) = paste("GS.", traitNames, sep="")

names(GSPvalue) = paste("p.GS.", traitNames, sep="")

trait="Treat"

traitColumn=match(trait,traitNames)

for (module in modNames){

column = match(module, modNames)

moduleGenes = moduleColors==module

if (nrow(geneModuleMembership[moduleGenes,]) > 1){

outPdf=paste("09.", trait, "_", module,".pdf",sep="")

pdf(file=outPdf,width=7,height=7)

par(mfrow = c(1,1))

verboseScatterplot(abs(geneModuleMembership[moduleGenes, column]),

abs(geneTraitSignificance[moduleGenes, traitColumn]),

xlab = paste("Module Membership in", module, "module"),

ylab = paste("Gene significance for ",trait),

main = paste("Module membership vs. gene significance\n"),

cex.main = 1.2, cex.lab = 1.2, cex.axis = 1.2, col = module)

abline(v=0.8,h=0.5,col="red")

dev.off()

}

}

probes = colnames(datExpr0)

geneInfo0 = data.frame(probes= probes,

moduleColor = moduleColors)

for (Tra in 1:ncol(geneTraitSignificance))

{

oldNames = names(geneInfo0)

geneInfo0 = data.frame(geneInfo0, geneTraitSignificance[,Tra],

GSPvalue[, Tra])

names(geneInfo0) = c(oldNames,names(geneTraitSignificance)[Tra],

names(GSPvalue)[Tra])

}

for (mod in 1:ncol(geneModuleMembership))

{

oldNames = names(geneInfo0)

geneInfo0 = data.frame(geneInfo0, geneModuleMembership[,mod],

MMPvalue[, mod])

names(geneInfo0) = c(oldNames,names(geneModuleMembership)[mod],

names(MMPvalue)[mod])

}

geneOrder =order(geneInfo0$moduleColor)

geneInfo = geneInfo0[geneOrder, ]

write.table(geneInfo, file = "GS_MM.xls",sep="\t",row.names=F)

for (mod in 1:nrow(table(moduleColors))){

modules = names(table(moduleColors))[mod]

probes = colnames(datExpr0)

inModule = (moduleColors == modules)

modGenes = probes[inModule]

write.table(modGenes, file =paste0("module_",modules,".txt"),sep="\t",row.names=F,col.names=F,quote=F)

}

geneSigFilter=0.5

moduleSigFilter=0.8

datMM=cbind(geneModuleMembership, geneTraitSignificance)

datMM=datMM[abs(datMM[,ncol(datMM)])>geneSigFilter,]

for(mmi in colnames(datMM)[1:(ncol(datMM)-2)]){

dataMM2=datMM[abs(datMM[,mmi])>moduleSigFilter,]

write.table(row.names(dataMM2), file =paste0("hubGenes_",mmi,".txt"),sep="\t",row.names=F,col.names=F,quote=F)

}

library(ConsensusClusterPlus)

expFile="GeneExp.txt"

workDir="C:\\Users\\HASEE\\Desktop\\18.cluster\\26.Cluster"

setwd(workDir)

data=read.table(expFile, header=T, sep="\t", check.names=F, row.names=1)

data=as.matrix(data)

group=sapply(strsplit(colnames(data),"\\_"), "[", 2)

data=data[,group=="Treat"]

maxK=9

results=ConsensusClusterPlus(data,

maxK=maxK,

reps=50,

pItem=0.8,

pFeature=1,

title=workDir,

clusterAlg="km",

distance="euclidean",

seed=123456,

plot="png")

clusterNum=2

cluster=results[[clusterNum]][["consensusClass"]]

cluster=as.data.frame(cluster)

colnames(cluster)=c("m6Acluster")

letter=c("A","B","C","D","E","F","G")

uniqClu=levels(factor(cluster$m6Acluster))

cluster$m6Acluster=letter[match(cluster$m6Acluster, uniqClu)]

outTab=cbind(t(data), cluster)

outTab=rbind(ID=colnames(outTab), outTab)

write.table(outTab, file="m6Acluster.txt", sep="\t", quote=F, col.names=F)

library(limma)

library(ggplot2)

clusterFile="m6Acluster.txt"

rt=read.table(clusterFile, header=T, sep="\t", check.names=F, row.names=1)

data=rt[,1:(ncol(rt)-1),drop=F]

m6Acluster=as.vector(rt[,ncol(rt)])

data.pca=prcomp(data)

pcaPredict=predict(data.pca)

PCA=data.frame(PC1=pcaPredict[,1], PC2=pcaPredict[,2], m6Acluster=m6Acluster)

PCA.mean=aggregate(PCA[,1:2], list(m6Acluster=PCA$m6Acluster), mean)

bioCol=c("#ff7f0e","#1f77b4")

m6aCluCol=bioCol[1:length(levels(factor(m6Acluster)))]

veganCovEllipse<-function (cov, center = c(0, 0), scale = 1, npoints = 100) {

theta <- (0:npoints) * 2 * pi/npoints

Circle <- cbind(cos(theta), sin(theta))

t(center + scale * t(Circle %*% chol(cov)))

}

df_ell <- data.frame()

for(g in levels(factor(PCA$m6Acluster))){

df_ell <- rbind(df_ell, cbind(as.data.frame(with(PCA[PCA$m6Acluster==g,],

veganCovEllipse(cov.wt(cbind(PC1,PC2),

wt=rep(1/length(PC1),length(PC1)))$cov,

center=c(mean(PC1),mean(PC2))))), m6Acluster=g))

}

pdf(file="PCA.pdf", height=5, width=6.5)

ggplot(data = PCA, aes(PC1, PC2)) + geom_point(aes(color = m6Acluster)) +

scale_colour_manual(name="m6Acluster", values =m6aCluCol)+

theme_bw()+

theme(plot.margin=unit(rep(1.5,4),'lines'))+

geom_path(data=df_ell, aes(x=PC1, y=PC2, colour=m6Acluster), size=1, linetype=2)+

annotate("text",x=PCA.mean$PC1, y=PCA.mean$PC2, label=PCA.mean$m6Acluster, cex=7)+

theme(panel.grid.major = element_blank(), panel.grid.minor = element_blank())

dev.off()

library(limma)

library(pheatmap)

library(reshape2)

library(ggpubr)

clusterFile="m6Acluster.txt"

rt=read.table(clusterFile, header=T, sep="\t", check.names=F, row.names=1)

rt=rt[order(rt$m6Acluster),]

data=t(rt[,1:(ncol(rt)-1),drop=F])

Type=rt[,ncol(rt),drop=F]

bioCol=c("blue","red")

ann_colors=list()

m6aCluCol=bioCol[1:length(levels(factor(Type$m6Acluster)))]

names(m6aCluCol)=levels(factor(Type$m6Acluster))

ann_colors[["m6Acluster"]]=m6aCluCol

pdf("heatmap.pdf", width=4, height=5)

pheatmap(data,

annotation=Type,

annotation_colors = ann_colors,

color = colorRampPalette(c(rep("#1f77b4",2), "white", rep("#ff7f0e",2)))(100),

cluster_cols =F,

cluster_rows =T,

scale="row",

show_colnames=F,

show_rownames=T,

fontsize=6,

fontsize_row=7,

fontsize_col=6)

dev.off()

data=melt(rt, id.vars=c("m6Acluster"))

colnames(data)=c("m6Acluster", "Gene", "Expression")

p=ggboxplot(data, x="Gene", y="Expression", color = "m6Acluster",

ylab="Gene expression",

xlab="",

legend.title="m6Acluster",

palette = m6aCluCol,

width=1)

p=p+rotate_x_text(60)

p1=p+stat_compare_means(aes(group=m6Acluster),

symnum.args=list(cutpoints = c(0, 0.001, 0.01, 0.05, 1), symbols = c("***", "**", "*", " ")),

label = "p.signif")

pdf(file="boxplot.pdf", width=10, height=5)

print(p1)

dev.off()

library(reshape2)

library(ggpubr)

library(limma)

library(GSEABase)

library(GSVA)

expFile="normalize.txt"

gmtFile="immune.gmt"

clusterFile="m6Acluster.txt"

rt=read.table(expFile, header=T, sep="\t", check.names=F)

rt=as.matrix(rt)

rownames(rt)=rt[,1]

exp=rt[,2:ncol(rt)]

dimnames=list(rownames(exp),colnames(exp))

data=matrix(as.numeric(as.matrix(exp)),nrow=nrow(exp),dimnames=dimnames)

data=avereps(data)

geneSets=getGmt(gmtFile, geneIdType=SymbolIdentifier())

ssgseaScore=gsva(data, geneSets, method='ssgsea', kcdf='Gaussian', abs.ranking=TRUE)

normalize=function(x){

return((x-min(x))/(max(x)-min(x)))}

ssgseaScore=normalize(ssgseaScore)

ssgseaOut=rbind(id=colnames(ssgseaScore), ssgseaScore)

write.table(ssgseaOut,file="ssGSEA.result.txt",sep="\t",quote=F,col.names=F)

cluster=read.table(clusterFile, header=T, sep="\t", check.names=F, row.names=1)

ssgseaScore=t(ssgseaScore)

sameSample=intersect(row.names(ssgseaScore), row.names(cluster))

ssgseaScore=ssgseaScore[sameSample,,drop=F]

cluster=cluster[sameSample,"m6Acluster",drop=F]

scoreCluster=cbind(ssgseaScore, cluster)

data=melt(scoreCluster, id.vars=c("m6Acluster"))

colnames(data)=c("m6Acluster", "Immune", "Fraction")

bioCol=c("#1f77b4","#ff7f0e")

bioCol=bioCol[1:length(levels(factor(data[,"m6Acluster"])))]

p=ggboxplot(data, x="Immune", y="Fraction", color="m6Acluster",

xlab="",

ylab="Immune infiltration",

legend.title="Cluster",

palette=bioCol)

p=p+rotate_x_text(50)

pdf(file="boxplot.pdf", width=10, height=6)

p+stat_compare_means(aes(group=m6Acluster),symnum.args=list(cutpoints = c(0, 0.001, 0.01, 0.05, 1), symbols = c("***", "**", "*", "")),label = "p.signif")

dev.off()

library(limma)

library(pheatmap)

library(reshape2)

library(ggpubr)

clusterFile="m6Acluster.txt"

ssgseaFile="ssGSEA.result.txt"

cluster=read.table(clusterFile, header=T, sep="\t", check.names=F, row.names=1)

cluster=cluster[,-ncol(cluster)]

ssgsea=read.table(ssgseaFile, header=T, sep="\t", check.names=F, row.names=1)

ssgsea=t(ssgsea)

sameSample=intersect(row.names(cluster), row.names(ssgsea))

cluster=cluster[sameSample,,drop=F]

ssgsea=ssgsea[sameSample,,drop=F]

cor=cor(ssgsea, cluster, method="spearman")

pdf(file="heatmap.pdf", width=12, height=6)

pheatmap(cor,

color = colorRampPalette(c(rep("#1f77b4",1), "white", rep("#ff7f0e",1)))(100),

cluster_cols =F,

cluster_rows =T,

display_numbers = T,

show_colnames=T,

show_rownames=T,

angle_col =45,

fontsize=9,

fontsize_row=8,

fontsize_col=8)

dev.off()

smr-1.3.1.exe --beqtl-summary ./eQTL/GTEx_V8/Lung.lite --query 1 --genes gene.list --out multiple --make-besd

smr-1.3.1.exe --bfile ./g1000_eur/g1000_eur --gwas-summary ./outcome/IPF1/gwas_IPF.txt --beqtl-summary ./results/03.GTEx_eQTL_IPF1_GWAS/multiple --out mysmr --thread-num 10

smr-1.3.1.exe --bfile ./g1000_eur/g1000_eur --gwas-summary ./outcome/IPF1/gwas_IPF.txt --beqtl-summary ./eQTL/GTEx_V8/Lung.lite --out myplot --plot --probe ENSG00000143167 --probe-wind 500 --gene-list glist-hg19

source("./plot_SMR.r")

smrdata=ReadSMRData("./myplot.ENSG00000139567(blood).txt")

SMRLocusPlot(data=smrdata,smr_thresh = 0.05,heidi_thresh = 0.05,plotWindow = 1000,anno_selfdef=FALSE)

SMREffectPlot(data=smrdata, trait_name="IPF",)

library(limma)

library(ggpubr)

expFile="normalize.txt"

geneFile="hubgene.csv"

cliFile="clinical.txt"

rt=read.table(expFile, header=T, sep="\t", check.names=F)

rt=as.matrix(rt)

rownames(rt)=rt[,1]

exp=rt[,2:ncol(rt)]

dimnames=list(rownames(exp), colnames(exp))

data=matrix(as.numeric(as.matrix(exp)), nrow=nrow(exp), dimnames=dimnames)

rt=avereps(data)

qx=as.numeric(quantile(rt, c(0, 0.25, 0.5, 0.75, 0.99, 1.0), na.rm=T))

LogC=( (qx[5]>100) || ( (qx[6]-qx[1])>50 && qx[2]>0) )

if(LogC){

rt[rt<0]=0

rt=log2(rt+1)}

data=normalizeBetweenArrays(rt)

geneRT=read.csv("hubgene.csv", header=T, sep=",", check.names=F)

sameGene=intersect(as.vector(geneRT[,1]),row.names(data))

data=data[sameGene,,drop=F]

data=t(data)

clinical=read.table(cliFile, header=T, sep="\t", check.names=F, row.names=1)

colnames(clinical)=c("Type")

sameSample=intersect(row.names(data), row.names(clinical))

data=cbind(data[sameSample,,drop=F], clinical[sameSample,,drop=F])

group=levels(factor(data[,"Type"]))

comp=combn(group,2)

my_comparisons=list()

for(i in 1:ncol(comp)){my_comparisons[[i]]<-comp[,i]}

for(i in colnames(data)[1:(ncol(data)-1)]){

rt1=data[,c(i,"Type")]

boxplot=ggviolin(rt1, x="Type", y=i, fill="Type",

xlab="",

ylab=paste0(i, " expression"),

legend.title="",

palette = c("#ff7f0e","#1f77b4"),

width=1, add = "boxplot", add.params = list(fill="white"))+

stat_compare_means(comparisons = my_comparisons,symnum.args=list(cutpoints=c(0, 0.001, 0.01, 0.05, 1), symbols=c("***", "**", "*", "ns")), label="p.signif")

#stat_compare_means(comparisons = my_comparisons)

pdf(file=paste0("violin.",i,".pdf"), width=5, height=4.5)

print(boxplot)

dev.off()

}

library(pROC)

expFile="normalize.txt"

geneFile="hubgene.txt"

rt=read.table(expFile, header=T, sep="\t", check.names=F, row.names=1)

y=gsub("(.*)\\_(.*)", "\\2", colnames(rt))

y=ifelse(y=="Control", 0, 1)

geneRT=read.table(geneFile, header=F, sep="\t", check.names=F)

bioCol=rainbow(nrow(geneRT), s=0.9, v=0.9)

aucText=c()

k=0

for(x in as.vector(geneRT[,1])){

k=k+1

roc1=roc(y, as.numeric(rt[x,]))

if(k==1){

pdf(file="ROC.genes.pdf", width=5.5, height=5.5)

plot(roc1, print.auc=F, col=bioCol[k], legacy.axes=T, main="")

aucText=c(aucText, paste0(x,", AUC=",sprintf("%.3f",roc1$auc[1])))

}else{

plot(roc1, print.auc=F, col=bioCol[k], legacy.axes=T, main="", add=TRUE)

aucText=c(aucText, paste0(x,", AUC=",sprintf("%.3f",roc1$auc[1])))

}

}

legend("bottomright", aucText, lwd=2, bty="n", col=bioCol[1:(ncol(rt)-1)])

dev.off()

library(limma)

library(survival)

library(survminer)

expFile="GeneExp.txt"

cliFile="FREIBURG.txt"

rt=read.table(expFile, header=T, sep="\t", check.names=F)

rt=as.matrix(rt)

rownames(rt)=rt[,1]

exp=rt[,2:ncol(rt)]

dimnames=list(rownames(exp), colnames(exp))

data=matrix(as.numeric(as.matrix(exp)), nrow=nrow(exp), dimnames=dimnames)

data=avereps(data)

data=data[rowMeans(data)>0,]

data=t(data)

rownames(data)=gsub("(.*?)\\_(.*?)", "\\2", rownames(data))

cli=read.table(cliFile, header=T, sep="\t", check.names=F, row.names=1)

cli$futime=cli$futime/365

sameSample=intersect(row.names(data), row.names(cli))

data=data[sameSample,]

cli=cli[sameSample,]

rt=cbind(cli, data)

outTab=data.frame()

km=c()

for(i in colnames(rt[,3:ncol(rt)])){

cox <- coxph(Surv(futime, fustat) ~ rt[,i], data = rt)

coxSummary = summary(cox)

coxP=coxSummary$coefficients[,"Pr(>|z|)"]

outTab=rbind(outTab,

cbind(id=i,

HR=coxSummary$conf.int[,"exp(coef)"],

HR.95L=coxSummary$conf.int[,"lower .95"],

HR.95H=coxSummary$conf.int[,"upper .95"],

pvalue=coxSummary$coefficients[,"Pr(>|z|)"])

)

data=rt[,c("futime", "fustat", i)]

colnames(data)=c("futime", "fustat", "gene")

res.cut=surv_cutpoint(data, time = "futime", event = "fustat", variables =c("gene"))

res.cat=surv_categorize(res.cut)

fit=survfit(Surv(futime, fustat) ~gene, data = res.cat)

#print(paste0(i, " ", res.cut$cutpoint[1]))

diff=survdiff(Surv(futime, fustat) ~gene,data =res.cat)

pValue=1-pchisq(diff$chisq, df=1)

km=c(km, pValue)

if(pValue<1){

if(pValue<0.001){

pValue="p<0.001"

}else{

pValue=paste0("p=",sprintf("%.03f",pValue))

}

surPlot=ggsurvplot(fit,

data=res.cat,

pval=pValue,

pval.size=6,

legend.title=i,

legend.labs=c("high","low"),

xlab="Time(years)",

ylab="Overall survival",

palette=c("#ff7f0e", "#1f77b4"),

break.time.by=1,

conf.int=F,

risk.table=T,

risk.table.title="",

risk.table.height=.25)

pdf(file=paste0("sur.", i, ".pdf"),onefile = FALSE,

width = 5,

height =5)

print(surPlot)

dev.off()

}

}

outTab=cbind(outTab, km)

write.table(outTab,file="uniCox.txt",sep="\t",row.names=F,quote=F)

inputFile="normalize.txt"

geneFile="hubgene.txt"

cliFile="clinical(FVC).txt"

data=read.table(inputFile, header=T, sep="\t", check.names=F, row.names=1)

row.names(data)=gsub("-", "_", row.names(data))

geneRT=read.table(geneFile, header=F, sep="\t", check.names=F)

data=data[as.vector(geneRT[,1]),]

group=sapply(strsplit(colnames(data),"\\_"), "[", 2)

data=data[,group=="Treat"]

colnames(data)=gsub("_Treat", "", colnames(data))

clinical=read.table(cliFile, header=T, sep="\t", check.names=F, row.names=1)

cliName=colnames(clinical)[1]

sameSample=intersect(colnames(data), row.names(clinical))

clinical=clinical[sameSample,,drop=F]

data=data[,sameSample,drop=F]

data1<-t(data)

data1<-as.data.frame(data1)

shapiro.test(data1$GPA33)

shapiro.test(clinical$Dlco)

shapiro.test(clinical$FEV1)

shapiro.test(clinical$FVC)

shapiro.test(clinical$age)

library(limma)

library(ggplot2)

library(ggpubr)

library(ggExtra)

inputFile="normalize.txt"

geneFile="hubgene.txt"

cliFile="clinical(DLco).txt"

data=read.table(inputFile, header=T, sep="\t", check.names=F, row.names=1)

row.names(data)=gsub("-", "_", row.names(data))

geneRT=read.table(geneFile, header=F, sep="\t", check.names=F)

data=data[as.vector(geneRT[,1]),]

group=sapply(strsplit(colnames(data),"\\_"), "[", 2)

data=data[,group=="Treat"]

colnames(data)=gsub("_Treat", "", colnames(data))

clinical=read.table(cliFile, header=T, sep="\t", check.names=F, row.names=1)

cliName=colnames(clinical)[1]

sameSample=intersect(colnames(data), row.names(clinical))

clinical=clinical[sameSample,,drop=F]

data=data[,sameSample,drop=F]

y=as.numeric(clinical[,cliName])

outTab=data.frame()

for(i in row.names(data)){

x=as.numeric(data[i,])

corT=cor.test(x, y, method = 'spearman')

cor=corT$estimate

pvalue=corT$p.value

outTab=rbind(outTab, cbind(Gene=i, Clinical=cliName, cor, pvalue))

df1=as.data.frame(cbind(x,y))

p1=ggplot(df1, aes(x, y)) +

xlab(i)+ylab(cliName)+

geom_point()+ geom_smooth(method="lm",formula = y ~ x) + theme_bw()+

stat_cor(method = 'spearman', aes(x =x, y =y))

p2=ggMarginal(p1, type = "density", xparams = list(fill = "#ff7f0e"),yparams = list(fill = "#1f77b4"))

pdf(file=paste0(cliName,"_",i,".pdf"), width=5, height=4.5)

print(p2)

dev.off()

}

write.table(file="corResult.txt",outTab,sep="\t",quote=F,row.names=F)

library(reshape2)

library(ggpubr)

inputFile="normalize.txt"

source("CIBERSORT.R")

outTab=CIBERSORT("ref.txt", inputFile, perm=1000, QN=T)

outTab=outTab[outTab[,"P-value"]<0.05,]

outTab=as.matrix(outTab[,1:(ncol(outTab)-3)])

outTab=rbind(id=colnames(outTab),outTab)

write.table(outTab, file="CIBERSORT-Results.txt", sep="\t", quote=F, col.names=F)

rt=read.table("CIBERSORT-Results.txt", header=T, sep="\t", check.names=F, row.names=1)

con=grepl("_Control", rownames(rt), ignore.case=T)

treat=grepl("_Treat", rownames(rt), ignore.case=T)

conData=rt[con,]

treatData=rt[treat,]

conNum=nrow(conData)

treatNum=nrow(treatData)

data=t(rbind(conData,treatData))

pdf(file="barplot.pdf", width=20, height=8)

col=rainbow(nrow(data), s=0.7, v=0.7)

par(las=1,mar=c(8,5,4,16),mgp=c(3,0.1,0),cex.axis=1.5)

a1=barplot(data,col=col,xaxt="n",yaxt="n",ylab="Relative Percent",cex.lab=1.8)

a2=axis(2,tick=F,labels=F)

axis(2,a2,paste0(a2*100,"%"))

par(srt=0,xpd=T)

rect(xleft = a1[1]-0.5, ybottom = -0.01, xright = a1[conNum]+0.5, ytop = -0.06,col="green")

text(a1[conNum]/2,-0.035,"Control",cex=1.8)

rect(xleft = a1[conNum]+0.5, ybottom = -0.01, xright =a1[length(a1)]+0.5, ytop = -0.06,col="red")

text((a1[length(a1)]+a1[conNum])/2,-0.035,"Treat",cex=1.8)

ytick2 = cumsum(data[,ncol(data)])

ytick1 = c(0,ytick2[-length(ytick2)])

legend(par('usr')[2]*0.98,par('usr')[4],legend=rownames(data),col=col,pch=15,bty="n",cex=1.2)

dev.off()

Type=gsub("(.*)\\_(.*)", "\\2", rownames(rt))

data=cbind(as.data.frame(t(data)), Type)

data=melt(data, id.vars=c("Type"))

colnames(data)=c("Type", "Immune", "Expression")

group=levels(factor(data$Type))

bioCol=c("#1f77b4","#ff7f0e")

bioCol=bioCol[1:length(group)]

boxplot=ggboxplot(data, x="Immune", y="Expression", fill="Type",

xlab="",

ylab="CIBERSORT Fraction",

legend.title="Type",

width=0.8,

palette=bioCol,add.params = list(size=0.1))

boxplot=boxplot+

stat_compare_means(aes(group=Type),symnum.args=list(cutpoints=c(0, 0.001, 0.01, 0.05, 1), symbols=c("***", "**", "*", "ns")), label="p.signif")+

theme_bw()+

rotate_x_text(50)

pdf(file="immune.diff.pdf", width=9, height=4.5)

print(boxplot)

dev.off()

inputFile="corResult.txt"

data0 = read.table(inputFile, header=T, sep="\t", check.names=F)

data = data0[,-1]

p.col = c('gold','pink','orange','LimeGreen','darkgreen')

fcolor = function(x,p.col){

color = ifelse(x>0.8,p.col[1],ifelse(x>0.6,p.col[2],ifelse(x>0.4,p.col[3],

ifelse(x>0.2,p.col[4], p.col[5])

)))

return(color)

}

p.cex = seq(2.5, 5.5, length=5)

fcex = function(x){

x=abs(x)

cex = ifelse(x<0.1,p.cex[1],ifelse(x<0.2,p.cex[2],ifelse(x<0.3,p.cex[3],

ifelse(x<0.4,p.cex[4],p.cex[5]))))

return(cex)

}

points.color = fcolor(x=data$pvalue,p.col=p.col)

data$points.color = points.color

points.cex = fcex(x=data$cor)

data$points.cex = points.cex

data=data[order(data$cor),]

xlim = ceiling(max(abs(data$cor))*10)/10

pdf(file="Lollipop.pdf", width=9, height=10)

layout(mat=matrix(c(1,1,1,1,1,0,2,0,3,0),nc=2),width=c(8,2.2),heights=c(1,2,1,2,1))

par(bg="white",las=1,mar=c(5,18,2,4),cex.axis=1.5,cex.lab=2)

plot(1,type="n",xlim=c(-xlim,xlim),ylim=c(0.5,nrow(data)+0.5),xlab="Correlation Coefficient",ylab="",yaxt="n",yaxs="i",axes=F)

rect(par('usr')[1],par('usr')[3],par('usr')[2],par('usr')[4],col="#F5F5F5",border="#F5F5F5")

grid(ny=nrow(data),col="white",lty=1,lwd=2)

segments(x0=data$cor,y0=1:nrow(data),x1=0,y1=1:nrow(data),lwd=4)

points(x=data$cor,y = 1:nrow(data),col = data$points.color,pch=16,cex=data$points.cex)

text(par('usr')[1],1:nrow(data),data$Cell,adj=1,xpd=T,cex=1.5)

pvalue.text=ifelse(data$pvalue<0.001,'<0.001',sprintf("%.03f",data$pvalue))

redcutoff_cor=0

redcutoff_pvalue=0.05

text(par('usr')[2],1:nrow(data),pvalue.text,adj=0,xpd=T,col=ifelse(abs(data$cor)>redcutoff_cor & data$pvalue<redcutoff_pvalue,"red","black"),cex=1.5)

axis(1,tick=F)

par(mar=c(0,4,3,4))

plot(1,type="n",axes=F,xlab="",ylab="")

legend("left",legend=c(0.1,0.2,0.3,0.4,0.5),col="black",pt.cex=p.cex,pch=16,bty="n",cex=2,title="abs(cor)")

par(mar=c(0,6,4,6),cex.axis=1.5,cex.main=2)

barplot(rep(1,5),horiz=T,space=0,border=NA,col=p.col,xaxt="n",yaxt="n",xlab="",ylab="",main="pvalue")

axis(4,at=0:5,c(1,0.8,0.6,0.4,0.2,0),tick=F)

dev.off()
